# Mouse lemur cell atlas informs primate genes, physiology and disease

**DOI:** 10.1038/s41586-025-09114-8

**Published:** 2025-07-30

**Authors:** Camille Ezran, Shixuan Liu, Stephen Chang, Jingsi Ming, Lisbeth A. Guethlein, Michael F. Z. Wang, Roozbeh Dehghannasiri, Julia Olivieri, Hannah K. Frank, Alexander Tarashansky, Winston Koh, Qiuyu Jing, Olga Botvinnik, Jane Antony, The Tabula Microcebus Consortium, Camille Ezran, Shixuan Liu, Stephen Chang, Jingsi Ming, Lisbeth A. Guethlein, Michael F. Z. Wang, Roozbeh Dehghannasiri, Julia Olivieri, Hannah K. Frank, Alexander Tarashansky, Winston Koh, Qiuyu Jing, Olga Botvinnik, Jane Antony, Iwijn De Vlaminck, Megan A. Albertelli, Caitlin J. Karanewsky, Jozeph L. Pendleton, Fabienne Aujard, Martine Perret, Liza Shapiro, Andriamahery Razafindrakoto, Hajanirina Noëline Ravelonjanahary, Patricia Wright, Anne D. Yoder, Cathy V. Williams, Robert Schopler, Ute Radespiel, Jean-Michel Verdier, Corinne Lautier, E. Christopher Kirk, Rebecca Lewis, Kerriann M. Casey, Kyle J. Travaglini, Astrid Gillich, Zicheng Zhao, Elias Godoy, Jérémy Terrien, Jacques Epelbaum, Dita Gratzinger, Katherine Lucot, Thomas Montine, Jessica D’Addabbo, Isaac Bakerman, Patricia Nguyen, Aaron Kershner, Karim Mrouj, Philip Beachy, Rahul Sinha, Yue Zhang, Irving L. Weissman, Thomas H. Ambrosi, Malachia Hoover, Alina Alam, Charles Chan, SoRi Jang, Avin Veerakumar, Peng Li, Andrea R. Yung, Connor V. Duffy, Song-Lin Ding, Ed S. Lein, Silvana Konermann, Liqun Luo, Trygve E. Bakken, Justus M. Kebschull, Rebecca D. Hodge, Taichi Isobe, Michael F. Clarke, Antoine de Morree, Biter Bilen, Jean Farup, Andoni Urtasun, Jengmin Kang, Thomas A. Rando, Ming Chen, BaoXiang Li, Varun Ramanan Subramaniam, Shravani Mukherjee, Aditi Swarup, Lily Kim, Bronwyn Scott, Ahmad Al-Moujahed, Albert Y. Wu, Douglas Vollrath, Lubert Stryer, Nicholas Schaum, Amanda L. Wiggenhorn, Tony Wyss-Coray, Yin Liu, Lolita Penland, Gabriel Loeb, Shengda Lin, Honor Paine, Deviana Burhan, Aris Taychameekiatchai, Steven Artandi, Bruce Wang, F. Hernán Espinoza, Christin Kuo, Ross Metzger, Norma Neff, Zhen Qi, Rebecca Culver, Kerwyn C. Huang, Patrick Neuhöfer, Charles A. Chang, Yan Hang, Seung K. Kim, Hannah N. W. Weinstein, Paul Allegakoen, Franklin W. Huang, Sivakamasundari V., Song Eun Lee, Kazuteru Hasegawa, Hosu Sin, Margaret T. Fuller, Wan-Jin Lu, Ankit Baghel, William Kong, Carly Israel, Rene Sit, Jennifer Okamoto, Ashley Maynard, Michelle Tan, Youcef Ouadah, Jalal Baruni, Timothy Ting-Hsuan Wu, Robert C. Jones, Maurizio Morri, Spyros Darmanis, Sheela Crasta, Jia Yan, Aditi Agrawal, Shelly Huynh, Brian Yu, James Webber, Jia Zhao, Gefei Wang, Weilun Tan, Saba Nafees, Zhengda Li, Stephen R. Quake, Geoff Stanley, Jinxurong Yang, Sheng Wang, Snigdha Agarwal, Kyle Awayan, Erin McGeever, Venkata N. P. Vemuri, Pranav V. Lalgudi, Angela Oliveira Pisco, Jim Karkanias, Can Yang, James E. Ferrell, Scott D. Boyd, Peter Parham, Jonathan Z. Long, Bo Wang, Julia Salzman, Angela Ruohao Wu, Stephen R. Quake, Mark A. Krasnow, Angela Oliveira Pisco, Jim Karkanias, Can Yang, James E. Ferrell, Scott D. Boyd, Peter Parham, Jonathan Z. Long, Bo Wang, Julia Salzman, Iwijn De Vlaminck, Angela Ruohao Wu, Stephen R. Quake, Mark A. Krasnow

**Affiliations:** 1 Department of Biochemistry, Stanford University School of Medicine, Stanford, CA USA; 2 Howard Hughes Medical Institute, Stanford University School of Medicine, Stanford, CA USA; 3 Department of Chemical and Systems Biology, Stanford University School of Medicine, Stanford, CA USA; 4 Division of Cardiovascular Medicine, Stanford University School of Medicine, Stanford, CA USA; 5 KLATASDS-MOE, School of Statistics and Academy of Statistics and Interdisciplinary Sciences, East China Normal University, Shanghai, China; 6 Department of Structural Biology, Stanford University School of Medicine, Stanford, CA USA; 7 Department of Microbiology and Immunology, Stanford University School of Medicine, Stanford, CA USA; 8 Nancy E. and Peter C. Meinig School of Biomedical Engineering, Cornell University, Ithaca, NY USA; 9 Department of Computational Biology, Cornell University, Ithaca, NY USA; 10 Department of Biomedical Data Science, Stanford University, Stanford, CA USA; 11 Institute for Computational and Mathematical Engineering, Stanford University, Stanford, CA USA; 12 Department of Pathology, Stanford University School of Medicine, Stanford, CA USA; 13 Department of Ecology and Evolutionary Biology, Tulane University, New Orleans, LA USA; 14 Department of Bioengineering, Stanford University, Stanford, CA USA; 15 Chan Zuckerberg Biohub, San Francisco, CA USA; 16 Institute of Bioengineering and Bioimaging, Agency of Science Technology and Research, Singapore, Singapore; 17 Bioinformatics Institute, Agency of Science Technology and Research, Singapore, Singapore; 18 Division of Life Science, Hong Kong University of Science and Technology, Hong Kong SAR, China; 19 Institute for Stem Cell Biology and Regenerative Medicine, Stanford University School of Medicine, Stanford, CA USA; 20 Department of Mathematics, Hong Kong University of Science and Technology, Hong Kong SAR, China; 21 Sarafan ChEM-H, Stanford, CA USA; 22 Department of Chemical and Biological Engineering, Hong Kong University of Science and Technology, Hong Kong SAR, China; 23 Center for Aging Science, Hong Kong University of Science and Technology, Hong Kong SAR, China; 24 Department of Applied Physics, Stanford University, Stanford, CA USA; 25 Department of Comparative Medicine, Stanford University School of Medicine, Stanford, CA USA; 26 Adaptive Mechanisms and Evolution (MECADEV), UMR 7179, National Center for Scientific Research, National Museum of Natural History, Brunoy, France; 27 Department of Anthropology, University of Texas at Austin, Austin, TX USA; 28 Department of Animal Biology, Faculty of Science, University of Antananarivo, Antananarivo, Madagascar; 29 Department of Anthropology, Stony Brook University, New York, NY USA; 30 Department of Biology, Duke University, Durham, NC USA; 31 Duke Lemur Center, Durham, NC USA; 32 Institute of Zoology, University of Veterinary Medicine Hannover, Hannover, Germany; 33 MMDN, University of Montpellier, EPHE-PSL, INSERM, Montpellier, France; 34 Unité Mixte de Recherche en Santé 894 INSERM, Centre de Psychiatrie et Neurosciences, Université Paris Descartes Sorbonne, Paris, France; 35 Stanford Cardiovascular Institute, Stanford, CA USA; 36 Department of Medicine, Stanford University School of Medicine, Stanford, CA USA; 37 Department of Developmental Biology, Stanford University School of Medicine, Stanford, CA USA; 38 Department of Urology, Stanford University School of Medicine, Stanford, CA USA; 39 Department of Biology, Stanford University, Stanford, CA USA; 40 Department of Genetics, Stanford University School of Medicine, Stanford, CA USA; 41 Human Cell Types Department, Allen Institute for Brain Science, Seattle, WA USA; 42 Department of Biomedical Engineering, Johns Hopkins School of Medicine, Baltimore, MD USA; 43 Department of Oncology and Social Medicine, Kyushu University, Fukuoka, Japan; 44 Department of Neurology and Neurological Sciences, Stanford University School of Medicine, Stanford, CA USA; 45 Department of Biomedicine, Aarhus University, Aarhus, Denmark; 46 Department of Ophthalmology, Stanford University School of Medicine, Stanford, CA USA; 47 Department of Neurobiology, Stanford University School of Medicine, Stanford, CA USA; 48 Department of Chemistry, Stanford University, Stanford, CA USA; 49 Wu Tsai Neurosciences Institute, Stanford, CA USA; 50 Division of Nephrology, Department of Medicine, University of California San Francisco, San Francisco, CA USA; 51 Zhejiang Provincial Key Laboratory for Cancer Molecular Cell Biology, Life Sciences Institute, Zhejiang University, Hangzhou, China; 52 Department of Medicine and Liver Center, University of California San Francisco, San Francisco, CA USA; 53 Stanford Cancer Institute, Stanford University School of Medicine, Stanford, CA USA; 54 Department of Pediatrics, Stanford University School of Medicine, Stanford, CA USA; 55 Stanford Diabetes Research Center, Stanford, CA USA; 56 JDRF Center of Excellence, Stanford, CA USA; 57 Division of Hematology/Oncology, Department of Medicine, University of California San Francisco, San Francisco, CA USA; 58 Helen Diller Family Comprehensive Cancer Center, University of California San Francisco, San Francisco, CA USA; 59 Bakar Computational Health Sciences Institute, University of California San Francisco, San Francisco, CA USA; 60 Department of Anesthesiology, Perioperative and Pain Medicine, Stanford University School of Medicine, Stanford, CA USA; 61 Paul G. Allen School of Computer Science and Engineering, University of Washington, Seattle, WA USA

**Keywords:** Transcriptomics, Cell biology

## Abstract

Mouse lemurs (*Microcebus* spp.) are an emerging primate model organism, but their genetics, cellular and molecular biology remain largely unexplored. In an accompanying paper^1^, we performed large-scale single-cell RNA sequencing of 27 organs from mouse lemurs. We identified more than 750 molecular cell types, characterized their transcriptomic profiles and provided insight into primate evolution of cell types. Here we use the generated atlas to characterize mouse lemur genes, physiology, disease and mutations. We uncover thousands of previously unidentified lemur genes and hundreds of thousands of new splice junctions including over 85,000 primate splice junctions missing in mice. We systematically explore the lemur immune system by comparing global expression profiles of key immune genes in health and disease, and by mapping immune cell development, trafficking and activation. We characterize primate-specific and lemur-specific physiology and disease, including molecular features of the immune program, lemur adipocytes and metastatic endometrial cancer that resembles the human malignancy. We present expression patterns of more than 400 primate genes missing in mice, many with similar expression patterns to humans and some implicated in human disease. Finally, we provide an experimental framework for reverse genetic analysis by identifying naturally occurring nonsense mutations in three primate immune genes missing in mice and by analysing their transcriptional phenotypes. This work establishes a foundation for molecular and genetic analyses of mouse lemurs and prioritizes primate genes, isoforms, physiology and disease for future study.

## Abstract (web summary)

Together with an accompanying paper presenting a transcriptomic atlas of the mouse lemur, interrogation of the atlas provides a rich body of data to support the use of the organism as a model for primate biology and health.

## Main

Many of the genes, pathways and principles of modern biology and the molecular foundations of medicine were uncovered through studies of canonical genetic model organisms. Nevertheless, new model organisms are being developed to study aspects of biology and medicine not observed or poorly recapitulated in the canonical models^2^. The expansion in emerging model organisms has been fuelled by genomic advances that have made reference genomes readily attainable and by gene editing tools such as CRISPR–Cas9 that have made the introduction of targeted mutations practical in many species. However, it remains challenging and time-consuming to establish a rich cellular, molecular and genetic understanding of a new model. We reasoned that organism-wide single-cell transcriptomics could greatly facilitate such understanding. In an accompanying paper^1^, we created a transcriptomic atlas of more than 750 cell types of the grey mouse lemur *Microcebus murinus*, an emerging primate model organism.

Mouse lemurs are an appealing model primate. Practical advantages include their small size, easy husbandry, short generation time and abundance in nature among primates^3^. Genomic sequence comparisons show that they are genetically intermediate between mice and humans^3^ (Supplementary Fig. 1). Moreover, transcriptomic comparisons using our newly reported atlas showed that expression patterns of many human genes and cell types are more similar to their lemur counterparts than those of mice^1^. The physiology of mouse lemurs has been studied for decades in laboratory colonies, especially their circadian and seasonal rhythms, metabolism, cognition and ageing^4,5^. Likewise, their ecology, behaviour and phylogeny have been investigated through field studies in Madagascar^6,7^. Here we use this new atlas^1^ to characterize mouse lemur genes, physiology, disease and mutations to provide a foundation for molecular and genetic studies of this model primate.

## The scRNA-seq atlas uncovers new genes

Our droplet-based (10x Genomics (10x)) and plate-based (Smart-seq2 (SS2)) single-cell RNA-sequencing (scRNA-seq) analyses of around 226,000 cells from 27 organs^1^ from 4 aged mouse lemur donors (L1–L4, with clinical and histological characterization^1,8^; Supplementary Note 2) provided an extensive amount of transcriptomic sequence information. About 2 × 10^12^ base pairs (around 10^12^ bp of high-quality reads each from 10x and SS2 sequencing) were distributed throughout the approximately 2.5 × 10^9 ^bp genome (Mmur 3.0 annotation^9^), which averaged around 10^4^-fold coverage of the transcriptome (about 2.5 × 10^8 ^bp of annotated transcripts from the National Center for Biotechnology Information (NCBI)). Such deep transcriptome coverage across most of the cell types of most organs can enhance gene detection, structure definition and annotation beyond current methods^10^, which rely primarily on phylogenetic sequence comparisons and bulk RNA sequencing, as done for *M.* *murinus* (NCBI annotation release 101, Ensembl genome browser v.100).

**Fig. 1:**
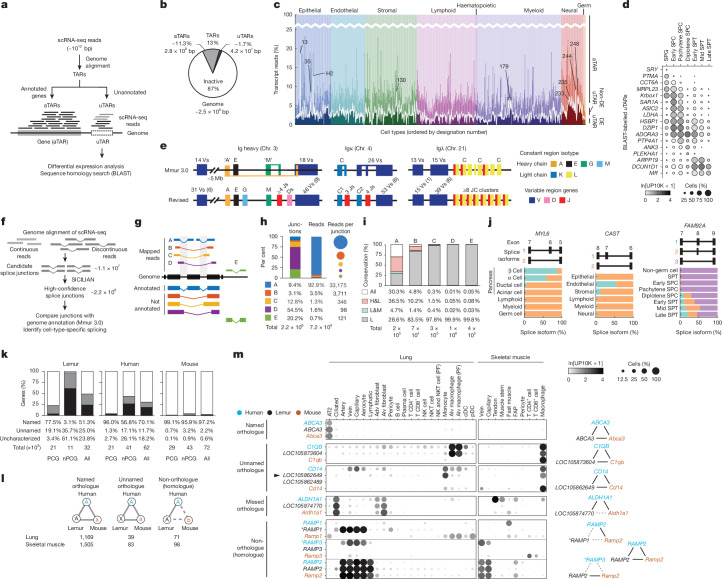
Organism-wide scRNA-seq uncovers new genes, splice forms and orthologues. **a–d**, Discovery of new genes (transcriptionally active regions, TARs). **f–j**, Discovery of new splice forms. **k–m**, Enhancement of gene annotation. **a**, Scheme for finding uTARs in the genome. **b**, Fraction of the genome (base pairs) that comprise uTARs and aTARs. **c**, Stacked bar plot showing the median percentage (transcript reads) of differentially expressed uTARs (DE uTARs), non-DE uTARs and aTARs for each atlas cell type. Example cell types enriched for DE uTARs are indicated by their designation number. 13, sweat gland; 35, enterocyte; H2, enterocyte/goblet; 130, pericyte; 179, basophil; 233, corticotroph; 235, lactotroph; 244, ependymal; 248, myelinating Schwann. **d**, Dot plot of mean expression (based on unique molecular identifier (UMI) counts: ln[UMI_gene_/UMI_total_ ×10^4^ + 1], abbreviated as ln[UP10K + 1] in dot heatmaps) and the percentage of cells (dot size) expressing the indicated DE uTARs during spermatogenesis. Gene names were assigned using a BLAST sequence homology search. **e**, Current (Mmur 3.0, top) and revised (using the scRNA-seq cell atlas, bottom) annotation of lemur immunoglobulin (Ig) loci. Numbers above gene clusters indicate the estimated number of functional genes and those in parentheses pseudogenes, lacking transcripts. **f**, Scheme for characterizing lemur splice junctions. Bars, exons; lines, introns. **g**, Splice junction categories. A, previously annotated; B–E, not annotated, including novel junctions between two annotated exon boundaries (for example, novel exon skipping, B), between annotated exon boundary and unannotated location in the gene (C), between two unannotated locations in the gene (D), and outside annotated genes (E). **h**, Percentage of total splice junction counts and reads and mean reads per junction for each category. **i**, Percentage of lemur splice junctions in each category that are conserved in both human and mouse genomes (All), only in human (H&L), only in mouse (L&M) or neither (L). **j**, Examples of genes (*MYL6*, *CAST* and *FAM92A*) with cell-specific and tissue-selective alternative splicing. Plots show the percentage of each isoform (coloured as in the diagram above) expressed in indicated cell types or compartments. **k**, Stacked bar plot showing the percentage of named (white), unnamed (grey) and uncharacterized (black) genes in lemur, human and mouse genomes, separated by protein-coding genes (PCGs), non-protein-coding (nPCGs) and all genes (All). **l**, Top, three types of human–lemur–mouse expression homologue triads. Left and middle, triads of sequence homologues with similar expression profiles that are assigned (NCBI and Ensembl) as orthologues (solid line) in all three species, and the lemur orthologue is named accordingly (left) or unnamed (middle). Right, triads of sequence homologues with similar expression profiles but not currently assigned as orthologues (dashed line) for at least one species. Bottom, number of each type when comparing lung or skeletal muscle cell-expression profiles. **m**, Dot plot comparison of the mean expression of selected expression homologue triads of each type across human, lemur and mouse lung and skeletal muscle cell types. Two lemur unnamed loci (*LOC105862649* and *LOC105862489*) are assigned (NCBI) as orthologues of mouse and human *CD14*, but only *LOC105862649* (arrowhead) is an expression homologue, which suggests that it is the true orthologue. *LOC105874770* is assigned as an orthologue of human *ALDH1A1* but not of mouse *Aldh1a1* (missed). For the three RAMP genes in each species, note that lemur *RAMP1* and human *RAMP3* are evolutionary outliers (asterisks), with both resembling the conserved *RAMP2* expression pattern. See also Extended Data Figs. 1–3 and Supplementary Fig. 2. Adv, adventitial; Alv, alveolar; AT2, alveolar type 2 cell; cDC, conventional dendritic cell; FAP, fibroadipogenic progenitor; pDC, plasmacytoid dendritic cell; PF, proliferating; SPC, spermatocyte; SPG, spermatogonium; SPT, spermatid. Source data

**Extended Data Fig. 1:**
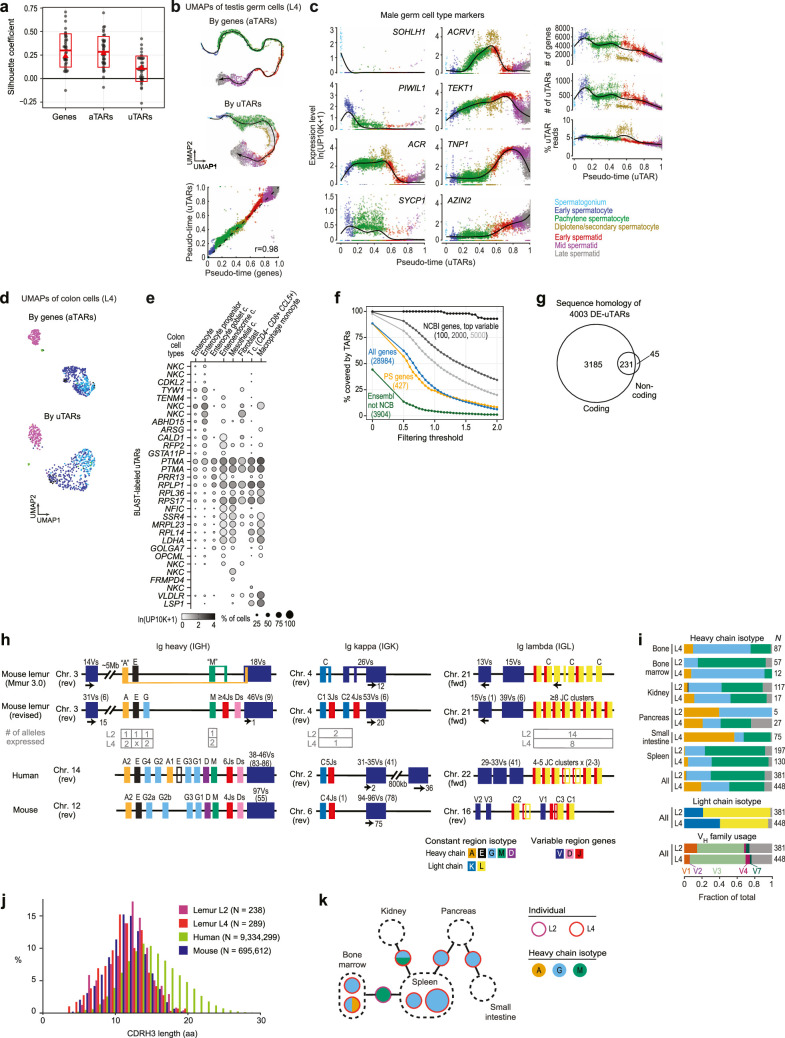
Comparison of expression patterns of uTARs and annotated genes, and Ig gene structures. **a**. Box plot of average silhouette coefficient values of the atlas datasets (separated by tissue, individual, and sequencing channel, N = 41) based on expression of annotated genes, aTARs, or uTARs. Box, mean±s.d.; red triangles, L4 colon example dataset as shown in panel d. Note the positive uTAR-based silhouette values of most datasets, supporting effective clustering of cells according to cell types by uTARs alone. **b**. UMAP of lemur male germ cells from testis (L4, 10x) embedded based on expression of either annotated genes^1^ (top) or uTARs alone (middle), colored by spermatogenesis stage (color code in c). Black line, pseudotime trajectory, with arrow indicating maturation direction; thin gray lines, individual cell alignments to trajectory. Dot plot (bottom) compares cellular pseudotime trajectory coordinates from annotated genes (x-axis) vs. uTARs (y-axis). Dashed black line, 1:1 relationship; *r*, Pearson’s correlation coefficient. **c**. Left, expression of selected sperm cell markers in germ cells ordered by the pseudotime developmental trajectory calculated by uTAR expression as in c. Right, number of expressed annotated genes (top) or uTARs (middle), and percent uTAR reads of total TARs (bottom), in each cell along trajectory. Note similar pattern of transcriptional downregulation of both uTARs and annotated genes during spermatogenesis. **d**. UMAP of colon cells (L4, 10x) embedded based on expression of annotated genes (top) or uTARs (bottom), colored by cell type as in e. **e**. Dot plot showing mean expression of selected DE uTARs across L4 colon cell types. Gene names for each DE uTAR based on sequence homology (identical names indicate multiple uTARs aligned to the same gene in another species). **f**. Percent of genes detected by TAR analysis as a function of the filtering threshold used to define cell type selective expression (i.e., TAR expression in any cell type ≥ e^threshold times that of the average of other cell types). Gene categories used include: the top 100 (black), 2000 (dark gray), and 5000 (light gray) variably-expressed genes annotated in Mmur 3.0 NCBI annotation, all genes (blue), PS genes (yellow), and genes annotated in Mmur 3.0 Ensembl annotations but missing from NCBI (green). **g**. Venn diagram of the 4003 DE-uTARs with sequence homology to coding regions (>1 hit by DIAMOND blastp analysis) and/or non-coding regions (>1 hit by Infernal cmscan analysis), according to Nf-predictorthologs analysis. **h**. Extension of schematic in Fig. 1e with lemur (top), human^68,89^ (middle) and mouse^68^ (bottom) Ig loci for heavy chain (left) and κ (center) and λ (right) light chains, located on the forward (fwd) or reverse (rev) strand of the indicated chromosomes (chr) and colored as in key. Top lemur line shows annotation as in NCBI’s Annotation Release 101 of Mmur 3.0; line below shows revised annotation using the atlas. Filled boxes, constant (A, E, G, D, M for heavy, C for light chains), variable (V), joining (J) and diversity (D) regions; open boxes, pseudogenes. Above V regions are the estimated number of functional V genes (varies per individual) and, in parentheses, the estimated number of V pseudogenes (lack transcripts). Note smaller V cluster ~5 Mb downstream of constant region in heavy locus which may be an assembly error (main V cluster is upstream). Arrows below clusters indicate genes oriented opposite to direction of constant regions, and those with numbers indicate subset of those genes that are flipped. Values below lemur loci in gray indicate number of expressed alleles for each constant region isotype in each lemur profiled. **i**. Bar graph showing fraction of B and plasma cells (SS2) by their expressed Ig heavy chain (top), light chain (middle) isotype, and heavy chain variable domain (VH) family member (bottom), separated by individual (L2, L4) and colored as in panel h (gray, unassigned isotype). N, number of cells analyzed. Fractions for heavy chain isotypes are also shown separately for organs with ≥5 cells, revealing tissue specialization (e.g., IGA-expressing cells prominent in small intestine and pancreas). Note V_H_ gene families related to human *IGHV1*, *3* and *4*, show the broadest expression, as in human and mouse^68,90,91^; however, light chain isotype *IGL* is more commonly expressed than *IGK*, in contrast to human and mouse B cells where *IGK* predominates^92^. **j**. CDRH3 lengths (number of amino acids, aa) for L2 and L4 (SS2), compared to that of healthy humans and lab mice. N, number of analyzed cells for lemurs and number of unique clones for humans and mice, including all isotypes. Human and mouse data courtesy of Scott Boyd’s group and Tho Pham^93,94^ (source data). CDRH3 lengths over 30 are not displayed because they are rare in human/mouse and did not occur in the lemurs analyzed. Note CDRH3 length in lemur is generally shorter than in human and more comparable to that of mouse. Though it is known that CDRH3 region length varies with age, disease state, and B cell maturity in order to affect antigen-binding affinity, the functional relevance of inter-species variation is unsettled^95^. **k**. B and plasma cell clones identified by their CDRH3 sequence. Each clone is represented as a filled circle, with its outline color indicating the lemur from which the clone was found and the fill color indicating the heavy chain isotype(s) of the clone. All clones consisted of two cells except in spleen which is a three-cell clone. Dashed outlines represent which organ(s) the constituent cells of the clone were found in. Circles between two dashed outlines indicate that the clone was found in both organs. Source data

To examine the value of this deep coverage, we first used the scRNA-seq data to systematically detect unannotated genes across the genome. A hidden Markov model approach^11^ was used to identify transcriptionally active regions (TARs), which are genomic locations with significant scRNA-seq coverage (Fig. 1a). TARs constituted 13% (3.3 × 10^8^ bp) of the genome, with most (87%, 2.8 × 10^8 ^bp, 11% of the genome) mapping to annotated genes (aTARs) (Fig. 1b). The rest (13%, 4.2 × 10^7 ^bp, 1.7% of the genome) mapped to unannotated regions (uTARs), which suggested that they could be unannotated genes. uTARs differentially expressed across cell types accounted for 2.4 ± 1.5% (mean ± s.d.) of the unique sequencing reads per cell, with up to 18.5% in sweat gland cells (Fig. 1c and Supplementary Tables 1 and 2). These differentially expressed uTARs are probably biologically significant because from their expression patterns alone, we could cluster cell types and reconstruct adult developmental programs (for example, spermatogenesis) with a consistency approaching that using annotated genes (Extended Data Fig. 1a–d).

To determine the gene identities of uTARs, we first confirmed that TAR analysis has high sensitivity for detecting previously annotated genes (Extended Data Fig. 1f). TARs captured almost all (98%, 4,884 genes) of the top 5,000 NCBI-annotated lemur genes with the highest cell-type expression variance in our scRNA-seq dataset. Moreover, they captured 44% (1,728; Supplementary Tables 1 and 2) of the 3,904 genes annotated by Ensembl but not NCBI, and 88% (376) of the 425 ‘primate-selective’ (PS) genes (see below). We then searched for homologues of the 4,003 differentially expressed lemur uTARs in other species (Fig. 1d, Extended Data Fig. 1e and Supplementary Table 1). DNA sequence searches (using BLASTn from the NCBI) identified homologous genes for 2,368 (59%) of these uTARs. Transcript and protein sequence searches (using DIAMOND blast and Infernal cmscan) identified protein-coding hits for 3,185 (80%) genes, non-coding hits for 45 (1%) genes and coding and non-coding hits for 231 (6%) genes (Extended Data Fig. 1g).

We also used our scRNA-seq datasets in a targeted approach to aid gene discovery in historically challenging loci. The B cell receptor (BCR) loci, which contain immunoglobulin genes, are difficult to annotate because they comprise large arrays of related, rapidly evolving genes and gene segments. Moreover, some segments are extremely short (for example, 10 bp diversity (D) segments) and widely spaced, and are brought together by variable–diversity–joining (V(D)J) recombination during B cell development to create antibody diversity^12^. Genomic mapping of the immunoglobulin heavy chain (*IGH* locus) transcripts in B cells and plasma cells (SS2 dataset) revised *IGA* and *IGM* gene structures and uncovered D and J gene clusters. Mapping also tripled the number of identified V genes (from 32 to 92) and identified 15 unexpressed V genes as probable pseudogenes (Fig. 1e and Extended Data Fig. 1h). Some expressed V genes mapped to a large V gene cluster about 5 Mb upstream of the rest of the *IGH* locus, which suggested that it is an orphan cluster. This atlas-enhanced annotation revealed that the lemur *IGH* locus has a similar organization to the human locus. However, the lemur locus is streamlined, with only a single constant region for each IGH isotype and no IGD, an evolutionarily plastic isotype lost in many lineages^13^. Hence the lemur provides a simplified model for understanding immunoglobulin gene rearrangement, expression and functions. Analyses of immunoglobulin light chain genes similarly enhanced the structure of *IGK* and *IGL* loci (Fig. 1e and Extended Data Fig. 1h). Thus, organism-wide scRNA-seq is an effective way of detecting missed genes throughout the genome, including complex, evolutionarily plastic regions.

## The scRNA-seq atlas defines splice isoforms

To enhance the characterization of gene structures and splice sites, we used the algorithm SICILIAN^14^ to uncover potential splice junctions from sequence reads that mapped to discontinuous positions along the genome (Fig. 1f and Supplementary Table 3). The current lemur genome annotation (NCBI, genome size of about 2.5 × 10^9 ^bp) has 212,198 assigned splice junctions, 41% fewer than in humans (358,924 in RefSeq hg38, genome size of around 3.1 × 10^9 ^bp) and 33% fewer than in mice (319,497 in RefSeq mm10, genome size of about 2.7 × 10^9 ^bp). These values suggest that thousands of lemur splice junctions remain to be discovered. Application of SICILIAN to our scRNA-seq dataset (Fig. 1f–h) computationally supported nearly all (98%, 202,802 junctions) of the currently annotated splice junctions. However, annotated junctions accounted for only 9.4% of the SICILIAN-identified junctions (category A). Newly identified junctions included 67,672 that had both 5′ and 3′ splice sites that have been previously separately annotated (category B; for example, exon skipping) and 274,991 between an annotated and a novel splice site (category C). Both types were supported by substantial scRNA-seq reads (on average, 3,711 (category B) and 346 (category C) unique reads per junction). SICILIAN analyses also detected new junctions between two novel splice sites (category D) and junctions that mapped to unannotated genes (category E). However, these junctions and sites were supported by fewer reads (98 and 121, respectively), which indicated that some could be a result of noise in splicing^15^ or are highly cell-type specific.

More than 85,000 of the lemur splice junctions were conserved in humans but missing in mice (Fig. 1i and Supplementary Table 3). Among the newly detected junctions, nearly 19,000 were conserved in humans and/or mice, most of which (59%) belonged to category B. These results suggest that organism-wide scRNA-seq combined with the SICILIAN algorithm can greatly enhance RNA splicing and gene structure characterization in a new reference genome. Moreover, this approach can be used to prioritize splice junctions and isoforms for further study, such as those present in primates but missing in mice.

**Extended Data Fig. 2:**
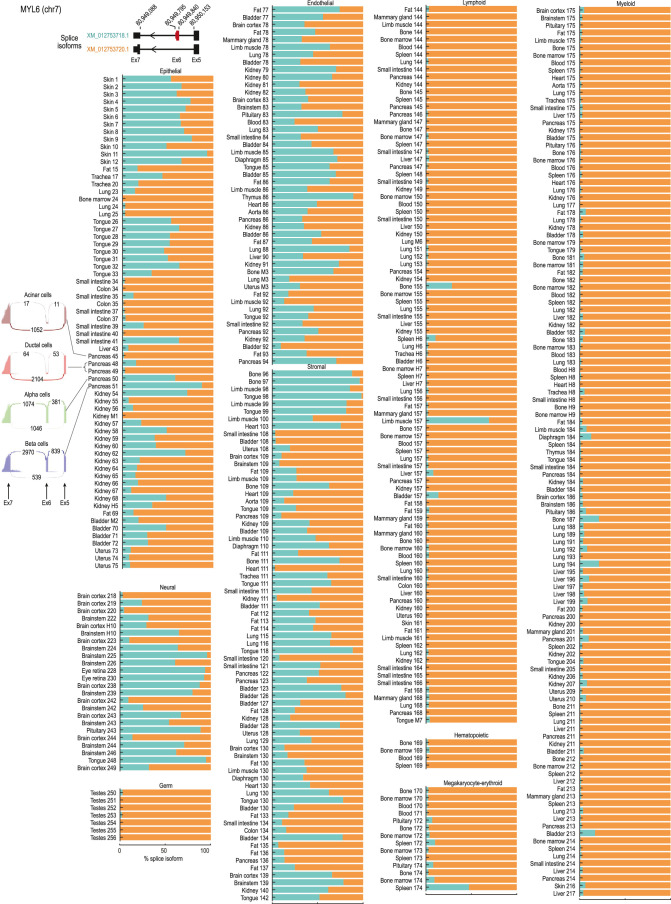
Expression of alternatively spliced isoforms across atlas cell types. Stacked bar graphs showing percent of indicated splice isoforms expressed across atlas cell types for MYL6 that is differentially spliced across compartments, formatted as in Fig. 1j. Cell types shown are those with spliced transcripts of the gene in ≥300 reads across ≥10 cells (except for sperm cells with fewer reads/cells). Cell types are labeled by their tissue source and designation number^1^, and colored by compartment. Top, transcript structure shown with splice isoforms labeled by corresponding NCBI Refseq ID and with exons affected by alternative splicing in red. Left, diagrams of mapped read buildups in Ex5-Ex7 genomic region are shown for the indicated cell types with weight of the connecting arcs reflecting the number of mapped reads (shown) that span each junction. See also Supplementary Fig. 2. Source data

We next performed differential splicing analysis using multivariate analysis of variance (MANOVA), which identified 545 genes that were the most differentially spliced across cell types in the atlas (Supplementary Table 4). For example, the gene *MYL6*, which encodes a myosin light chain that is ubiquitously expressed but poorly characterized^16^, can be alternatively spliced to include or skip exon 6. Both isoforms are produced in most cell types but their ratio can markedly differ. In pancreatic α and β cells, most transcripts included exon 6, whereas in ductal and acinar cells and most immune and germ cells, almost all transcripts excluded it (Fig. 1j and Extended Data Fig. 2). *CAST*, which encodes a regulator of membrane fusion, had its exon 7 included in about 50% of transcripts in endothelial cell types but almost always skipped in other cell types (Fig. 1j and Supplementary Fig. 2a). Numerous genes showed sperm-specific splicing and differential splicing during spermatogenesis (for example, *FAM92A*; Fig. 1j and Supplementary Fig. 2b–e).

## The scRNA-seq atlas aids gene annotation

Gene identity assignments in new reference genomes have traditionally relied on phylogenetic sequence comparisons and chromosomal positioning. That is, a gene is assigned a name that corresponds to the characterized homologue in other species with the greatest sequence similarity and conserved chromosomal gene order because such connections indicate a direct evolutionary relationship (orthologue). However, such analyses sometimes do not identify homologues for a gene or can uncover multiple homologues with similar sequence identity, thereby obscuring the true orthologue^10^. Hence, about a quarter of the genes (around 7,600) in the current lemur genome annotation (NCBI) have only a locus identifier (for example, ‘Loc_’ or ‘orf’) and no formal gene name or symbol or description (‘uncharacterized genes’). Moreover, another quarter (about 8,000) have an initial description from sequence homology but no name or symbol (‘unnamed genes’) (Fig. 1k). The fractions of these uncharacterized and unnamed genes in the current lemur genome annotation are much greater than those in the human and mouse genomes. Therefore, we sought to complement the classical approaches used for gene orthology assignment and naming by identifying the sequence homologue (or homologues) with the most conserved expression pattern (‘expression homologue’).

**Extended Data Fig. 3:**
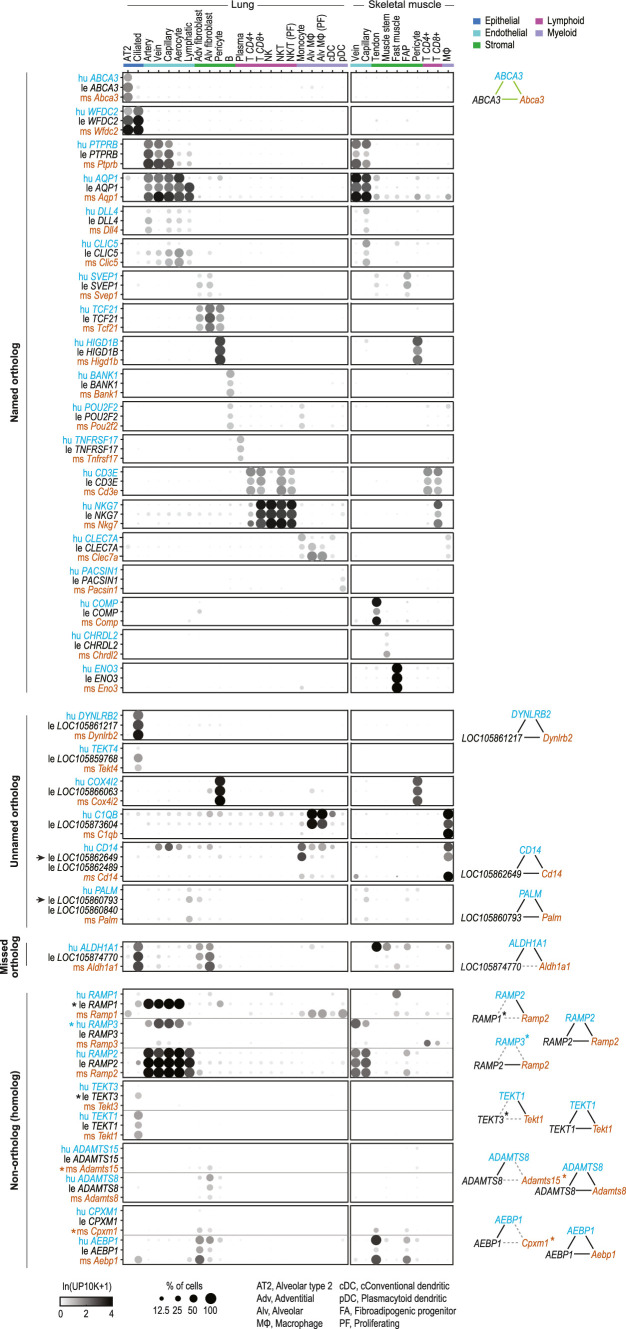
Additional examples of expression homologue triads. Dot plots as in Fig. 1m with additional examples of expression homologue triads (named, unnamed, missed orthologue, and non-orthologue types) across human, lemur, and mouse lung and skeletal muscle cell types indicated. Corresponding triad diagrams are shown on the right. Note, three *RAMP* expression homologue triads are detected: two non-orthologues (i.e., hu *RAMP2* - le RAMP1* - ms *Ramp2*, hu RAMP3* - le *RAMP2* - ms *Ramp2*) and one named orthologue (hu *RAMP2* - le *RAMP2* - ms *Ramp2*). Asterisk indicates the outlier non-orthologous gene. The shared expression patterns of lemur *RAMP1* and human *RAMP3* with *RAMP2* suggests that lemur RAMP1 and human RAMP3 have evolved to engage in similar physiological functions as the species-conserved RAMP2 or to modulate RAMP2-mediated ligand signaling in lung endothelial cells. Similar non-orthologous expression homologues were identified for *TEKT1/3*, *ADAMTS8/S15*, and *AEBP1/CPXM1*.

We used the algorithm SAMap^17^ to find for each lemur gene the mouse and human sequence homologues with the most similar expression patterns across 32 orthologous lung and muscle cell types, which we carefully curated in the same way for all three species. This strategy identified 1,279 expression homologue triads in lung and 1,686 in muscle (Fig. 1l,m, Extended Data Fig. 3 and Supplementary Table 5), most of which (91% lung, 89% muscle) were triads of named orthologous genes across the three species (for example, *ABCA3*). This result substantiates the orthology assignments of traditional approaches (Fig. 1l,m). We also identified 39 (3%) lung and 83 (5%) muscle orthologous gene triads that showed conserved expression patterns, but for which the lemur locus remained unnamed in the NCBI annotation. This finding indicates, for example, that the identified lemur gene *LOC105873604* is the orthologue of mouse and human *C1QB* and should be named accordingly (Fig. 1l,m and Extended Data Fig. 3). There were also instances whereby multiple unnamed lemur loci had the same NCBI description (for example, ‘monocyte differentiation antigen CD14-like’), but a comparison of expression patterns across species identified the probable orthologue (*LOC105862649* as *CD14* given its expression in lemur myeloid cells, and *LOC105862489* as a possible *CD14* pseudogene given its sparse expression) (Fig. 1m and Extended Data Fig. 3). We also found expression homologue triads with incomplete orthology assignments in the NCBI and Ensembl annotations that were completed by cross-species expression comparisons (for example, lemur *LOC105874770* is probably a missed orthologue of mouse *Aldh1a1*; Fig. 1m).

Notably, the analysis uncovered a small fraction (6%) of genes (71 out of 1,279 in lung, 98 out of 1,686 in muscle) for which the expression patterns were not conserved with their assigned orthologues (Fig. 1l,m and Extended Data Fig. 3). For example, in the lung, *RAMP1*, which encodes a hormone co-receptor, was highly expressed in endothelial cell types in lemurs, myeloid cell types in mice and sparsely in humans. In fact, lemur *RAMP1* shared a lung expression pattern most similar to *RAMP2*, which was selectively expressed in endothelial cells across all three species. These results suggest that lung endothelial and myeloid cells have species-specific responses to certain hormones^18^. This finding exemplifies rare, species-specific adaptations that have dissociated gene expression patterns from their conserved protein structure^1^. Examination of the expression patterns of homologues therefore provides another dimension for gene naming and orthology assignment and for exploring the diversification of gene expression in evolution.

**Extended Data Fig. 4:**
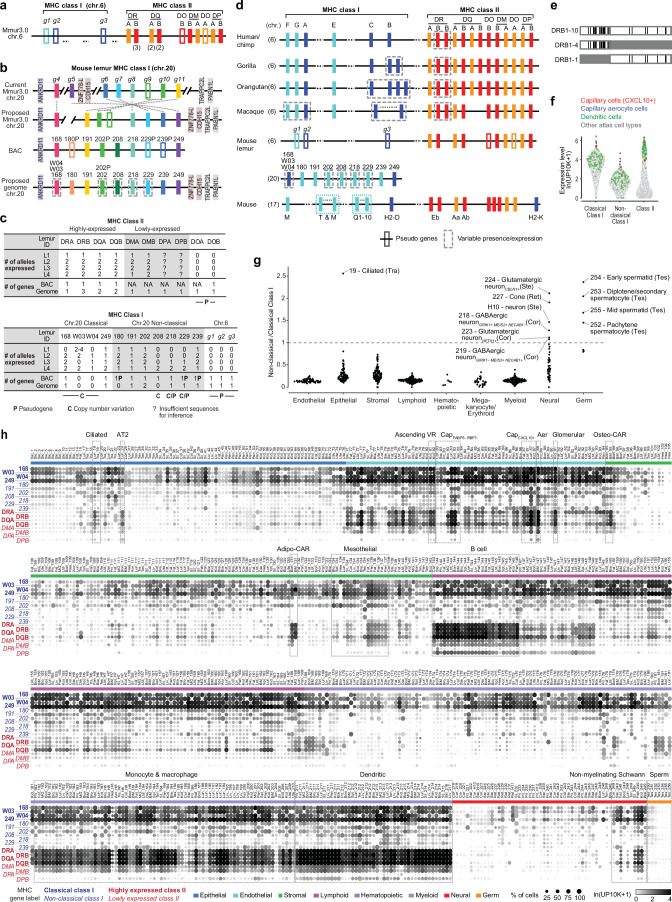
Structure and global expression pattern of mouse lemur MHC class I and II genes. **a**. Schematic of mouse lemur MHC locus on chromosome (chr.) 6. Filled rectangles, expressed genes; open rectangles, pseudogenes. Numbers below *DRB*, *DQA*, and *DQB* indicate the number of genes annotated by NCBI, though Guethlein et al.^21^ suggest a single gene for each family. Dashed lines, extended areas in genome assembly. g1, *LOC105855356* in NCBI; g2, *LOC105855357*; g3, *LOC105858107*. **b**. Schematic of MHC class I gene locus on lemur chr. 20. Top line, gene order as annotated by NCBI’s Refseq Annotation Release 101. The three genomic segments are separated by gaps (slanted lines) in Mmur 3.0 genome assembly. Second line, proposed reorganization of assembly based on rearrangement of three segments to match gene order of a sequenced BAC^75^ (third line). Note gene content in Mmur 3.0 assembly and BAC differ due to haplotype variability in the individuals sequenced. Dashed lines connect corresponding genes. Fourth (bottom) line, proposed revision of structure and annotation of this locus based on above and the expression pattern of these genes in the lemur atlas. Dashed boxes, genes varying in either presence, copy number or expression status. *W03* and *W04* are sequences derived from the original study on the lemur MHC^76^. Based on sequence similarity, *168*, *W03* and *W04* could represent divergent allelic variants (or separate genes). *202* (g10) and *202P* (g9) are a pair of phylogenetically related genes annotated by NCBI; there was no evidence supporting them as separate genes in atlas expression data, suggesting *202P* is a pseudogene, genomic polymorphism, or an assembly error. g4, *LOC105855949* in NCBI; g5, *LOC105855951*; g6, *LOC105870766*; g7, *LOC105870764*; g8, *LOC105870765*; g9, *LOC105870769*; g10, *LOC105870767*; g11, *LOC105870762*. **c**. Number of putative alleles for each MHC gene in the four lemurs (L1-4), and number of genes annotated in the BAC and by NCBI. Note some class I sequences differ only by a few base pairs among haplotypes^21^, therefore the number of alleles in the panel represents our best estimate but may be inexact due to sequencing errors or other technical artifacts. C, genes that exhibit copy number variation; P, genes that have at least one allele that is a pseudogene;?, insufficient number of reads for a reliable allele count; NA, gene absent from BAC sequence. **d**. Comparison of mouse lemur MHC class I and class II regions with that of other primates and mouse^74,75,96,97^. Note lemur MHC class I region on chr. 6 contains only pseudogenes (opened boxes) whereas functional class I genes (filled boxes) are translocated to chr. 20. Dark blue, classical MHC class I genes (high, widespread expression); light blue, non-classical MHC class I genes (lower expression and/or tissue-specific expression), orange, MHC class II A genes; red, MHC class II B genes. Gray dashed box, genes with haplotypic variability in gene number or expression status. Cyan dashed boxes enclose mouse MHC haplotype T, M, and Q regions with expanded gene families. Dashed lines, extended areas in genome assembly. **e**. Schematic of the three *Mimu-DRB* genes in lemur genome assembly Mmur 3.0. *DRB1-10* is predicted to encode a full length DRB1 polypeptide, whereas *DRB1-1* and *DRB1-4* are incomplete and contain non-MHC sequences (gray shading) but would together encode a functional DRB polypeptide, suggesting that these sequences were misassembled and belong together to form a second complete *DRB1* allele^21^. Thin vertical lines, positions that differ between the three sequences. **f**. Sina plots of summed expression of classical class I, non-classical class I, and class II MHC genes, respectively and averaged across cell types. **g**. Sina plots of ratio of summed non-classical to summed classical MHC class I expression, averaged across cell types and plotted separately by compartment. Note consistently lower levels of non-classical vs. classical MHC class I expression across almost all atlas cell types (dots), except a few highlighted cell types in the neural and germ compartments. Ste, brainstem; Cor, brain cortex; Ret, retina; Tes, testis. **h**. Dot plot of mean expression of each MHC gene across all atlas molecular cell types (10x, L1-L4), ordered by compartment and labeled by tissue and designation number^1^. Gray dashed boxes, cell types highlighted in main text. Source data

We also used the atlas to enhance annotation of the major histocompatibility complex (MHC) (Extended Data Fig. 4a–e), which encodes antigen-presenting proteins in adaptive immunity. The MHC is difficult to annotate because of its extreme evolutionary plasticity^19^, including some of the most polymorphic genes in the genome^20^ due to mutations, gene duplications and deletions that individualize immune systems and their response to infection. Allele-specific expression analysis of MHC class II genes across the atlas established gene copy numbers. The analysis also distinguished major (highly and broadly expressed) class II genes (*DQA*, *DQB*, *DRA* and *DRB*) from minor genes expressed at lower levels and in fewer cells (*DMA*, *DMB*, *DPA* and *DPB*) and unexpressed putative pseudogenes (*DOA* and *DOB*) (Extended Data Fig. 4c,h). A similar analysis of class I genes distinguished a cluster of non-expressed pseudogenes (chromosome 6) from a functional cluster (11 expressed genes on chromosome 20q) that included four with high and widespread expression, which we designate ‘classical’ (*Mimu-168*, *Mimu-W03*, *Mimu-W04* and *Mimu-249*), and three previously thought to be pseudogenes (*Mimu-180ps*, *Mimu-229ps* and *Mimu-239ps*) based on sequence analysis^21^ (Extended Data Fig. 4a–c,h and Supplementary Note 3).

Thus, organism-wide scRNA-seq is a powerful complement to phylogenetic sequence comparisons for the creation of a high-quality annotation of a genome.

## Immune expression, development and function

Little is known about the cell or molecular biology of lemurs. Our organism-wide transcriptomic atlas can expedite such understanding. Here we demonstrate how we used the atlas to examine lemur immune function, a physiologically important process with significant human–mouse differences^22^. We mapped global expression patterns of three key sets of immune genes and examined immune cells across the body to characterize their development, dispersal and activation. These analyses revealed general immune functions in lemur as well as primate specializations.

Classical MHC class I genes were highly and broadly expressed (Extended Data Fig. 4f–h), a result that reflects their widespread role in presenting peptides derived from cytosolic proteins to CD8^+^ T cells^23^. However, expression varied between compartments (highest in endothelial and immune, intermediate in stromal and epithelial, low in neural and germ cell), and even within a compartment there were significant cell-type differences (Extended Data Fig. 4f–h). For example, CXCL10^+^ capillary cells and lung capillary aerocytes showed the highest expression of MHC class I genes in the atlas, and non-myelinating Schwann cells were a notable exception to the general low expression of these genes in the neural compartment, suggesting special roles for these cell types in protecting the lung and peripheral nervous system against intracellular pathogens. MHC class II genes were more specifically expressed, notably in professional antigen-presenting cells (dendritic cells, macrophages and B cells) (Extended Data Fig. 4f,h), a result that reflects their role in presenting fragments of engulfed extracellular pathogens to CD4^+^ T cells^23^. However, they were also expressed across the endothelial compartment, like in humans but not in rodents^24^, and at particularly high levels in several capillary subtypes (Extended Data Fig. 4f,h). There was little expression in stromal, epithelial and neural compartments, except two stem cell niche cells (adipo-CXCL12-abundant reticular (adipo-CAR) and osteo-CAR cells) and some mesothelial and lung epithelial (ciliated, AT2) cells (Extended Data Fig. 4h). These high-expressing non-immune cells presumably have ‘non-professional’ roles alerting the immune system to extracellular pathogens.

Mapping BCR immunoglobulin gene expression established many classical features of B cell development and function in the lemur. These included expression in each B cell of a dominant immunoglobulin heavy and light chain isotype, and heavy chain isotype specificity by tissue, and class switching during B cell development with clonal expansion in different tissues. We also characterized the heavy chain complementarity-determining antigen-binding region (CDRH3) (Extended Data Fig. 1i–k and Supplementary Table 6).

**Fig. 2:**
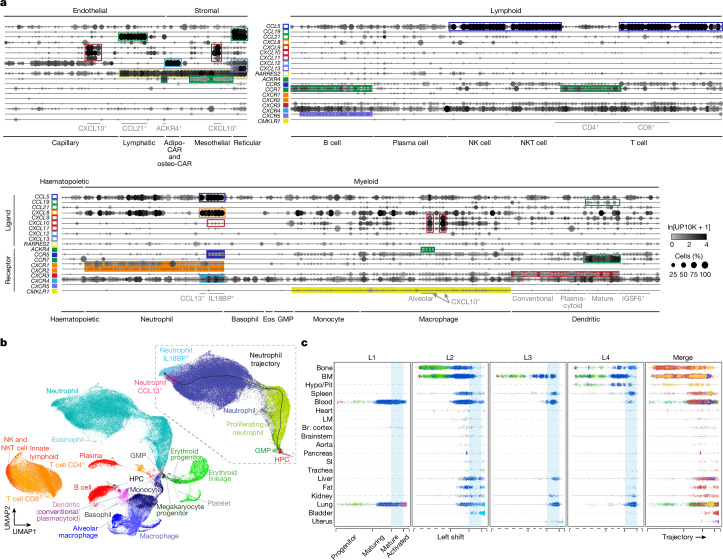
Organism-wide mapping of chemokine signalling and neutrophil maturation. **a**, Dot plot of the mean expression of selected chemokine receptors and their primary cognate ligands across immune and other major interacting cell types in the atlas (10x data). Boxes of the same colour highlight cell types highly expressing a receptor (filled boxes) and its ligand (open boxes). **b**, UMAP of immune cells in the atlas (10x and SS2 data) that were integrated using the FIRM algorithm across all tissues and individuals, coloured by major immune cell type. Inset (boxed), extracted neutrophil UMAP with cells coloured by cell type. Black line, pseudotime trajectory; thin grey lines, individual cell alignments to trajectory. **c**, Neutrophil lineage cells (dots; coloured as in **b** inset) along the pseudotime trajectory (*x* axis) separated by the source tissue (*y* axis) and individual lemur (L1–L4; merged on the right). The light blue background highlights the trajectory location of non-activated, mature neutrophils (the main circulating neutrophil population in health) with progenitor or maturing cells to the left and activated neutrophils to the right. Note that lemur L1 had progenitor cells in the blood, which implicated dysregulation of granulopoiesis. Lemur L2 had maturing neutrophils in the blood (clinically called ‘left shift’). Lemurs L1–L3 all had activated neutrophils in peripheral inflamed tissues, which was probably in response to infection or malignancy. Grey dashed lines indicate organs not profiled. See also Extended Data Figs. 5–8, Supplementary Notes 4–7 and Supplementary Figs. 3 and 4. BM, bone marrow; Br, brain; Eos, eosinophil; GMP, granulocyte–monocyte progenitor; HPC, haematopoietic precursor cell; Hypo/Pit, hypothalamus/pituitary gland; LM, limb muscle; SI, small intestine. Source data

**Extended Data Fig. 5:**
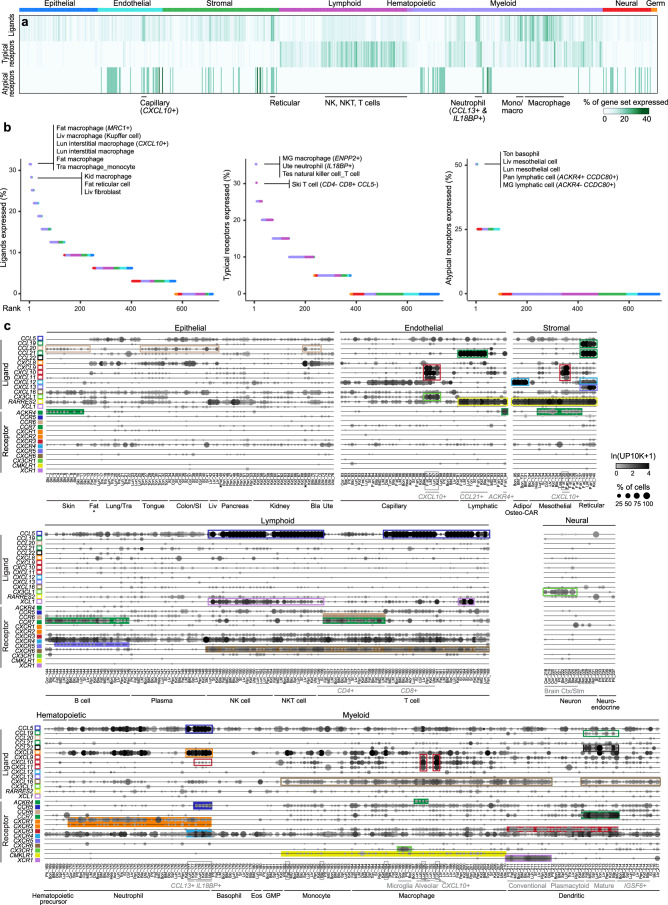
Expression of chemokines and receptors across atlas cell types. **a**. Heat map showing percent of chemokine ligands (n = 32), typical receptors (20), and atypical receptors (4) expressed across atlas cell types (10x, L1-L4), ordered by designation number and tissue. **b**. Rank of cell types based on the percent of expressed chemokine ligands (left), typical receptors (center), and atypical receptors (right). Cell types (dots) colored by tissue compartment. **c**. Extension of Fig. 2a with dot plot of mean expression of selected chemokine receptors and their primary cognate ligands across immune and other major interacting cell types in the atlas (10x). Gray boxes, cell types with inflammation and disease related expression patterns. See Supplementary Note 4 and Supplementary Fig. 3 for further analysis. Source data

Expression of chemokines (32 genes) and their receptors (24 genes) (Supplementary Table 7) provided insight into the regulation of immune cell trafficking (Fig. 2a, Extended Data Fig. 5 and Supplementary Fig. 3). Ligands were broadly expressed across non-germ cell compartments, whereas receptors were mostly restricted to immune populations (Extended Data Fig. 5a). For example, we identified specific cell types (adipo-CAR and osteo-CAR cells) that expressed *CXCL12*, which is thought to enable retention of haematopoietic progenitors (which express its receptor *CXCR4*) within bone marrow and regulate the release of maturing cells into the circulation as they downregulate receptor expression^25,26^ (Fig. 2a and Supplementary Fig. 3a). We also identified epithelial cell types that express *CCL20*, which is implicated in the recruitment of receptor *CCR6*-expressing immune cells^27^. Similarly, cortical and brainstem neurons were identified to express *CX3CL1*, which attracts *CX3CR1*-expressing microglia^28^. Finally, we identified cell types and chemokines that may direct diverse immune cells to lymph nodes (detailed in Extended Data Fig. 5c, Supplementary Note 4 and Supplementary Fig. 3c).

**Extended Data Fig. 6:**
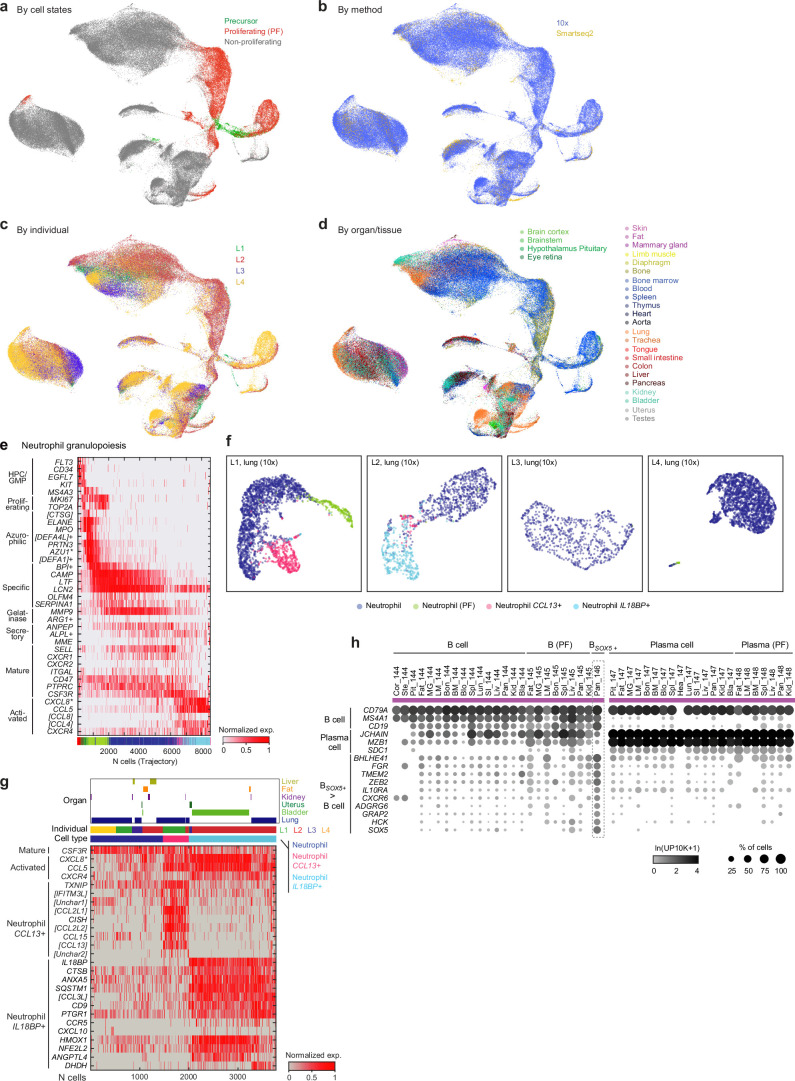
FIRM-integrated UMAP of atlas immune cells and further characterization of neutrophils and B lymphocytes. **a-d**. UMAP of atlas immune cells as in Fig. 2b but colored by proliferation state (**a**), scRNA-seq method (**b**), individual (**c**), and tissue of origin (**d**). **e**. Heatmap showing relative expression along neutrophil trajectory (10x) of lemur orthologues of human neutrophil markers. Colored bar indicates cell type designation as in Fig. 2b inset. For each gene, expression values (ln(UP10K + 1)) were normalized to its maximal value (99.5 percentile) in trajectory. Non-activated neutrophils in plot were uniformly subsampled (10%). Note human-like sequential expression of granulopoiesis marker genes; azurophilic (primary) granules (*AZU1*, *MPO*, *ELANE*) in early stages, followed by specific (secondary) granules (*LTF*, *CAMP*, *LCN2*), gelatinase granules (*MMP9*, *ARG1*), and finally secretory vesicles (*ALPL*, *MME*) in mature neutrophils. *, genes without a mouse orthologue; +, genes not expressed in mouse neutrophils. [], description of genes identified by NCBI as loci: *[CTSG], LOC105866609; [DEFA4L], LOC105881499; [DEFA1], LOC105881500; [CCL8], LOC105885739; [CCL4], LOC105881712*. **f**. UMAPs of lung neutrophils (10x) of the indicated individuals, with cells colored by cell type designation. Note the two subtypes of activated neutrophils (*CCL13+* and *IL18BP+*) cluster separately from the main neutrophil population (non-activated) in both L1 and L2. **g**. Heatmap showing relative expression of the indicated marker genes for mature and activated neutrophils as well as DEGs for each activated neutrophil subtypes. Bars at top show for each neutrophil, its tissue source (top set of bars), individual lemur source (middle), and cell type designation (bottom). Note both activated neutrophil subtypes were found in more than one individual and from multiple tissues. Expression values for each gene were normalized to the maximal value (99.5 percentile) for the gene across all cells in the neutrophil trajectory. The mature (non-activated) neutrophils are highly abundant so uniformly subsampled (20%) in plot at late stage (>0.7) of the pseudotime trajectory. *, genes without a mouse orthologue. *[IFITM3L], LOC105874071; [Uncharacterized 1], LOC105867541; [CCL2L], LOC105859340 and LOC105885684; [CCL13], LOC105859268; [Uncharacterized 2], LOC105856756; [CCL3L], LOC105881608*. **h**. Dot plot showing mean expression in B lymphocyte lineage cells of marker genes for B cells, plasma cells, and top DEGs in the *SOX5*+ B cell population compared to other B cells (10x, L1-L4). Lemur B cells and plasma cells in the atlas appear relatively homogenous molecularly, except for *SOX5*+ B cell population identified in L4’s pancreas (with nearby lymph nodes). See Supplementary Note 6 for further analysis. Source data

In addition to these local interactions, we globally mapped the lemur haematopoietic program beginning with bone marrow progenitors and continuing with their maturation, dispersal and differentiation throughout the organism and activation in specific tissues. The integrated immune cell uniform manifold approximation and projection (UMAP) plot (Fig. 2b and Extended Data Fig. 6a–d) reconstructed the developmental trajectories of major haematopoietic lineages. We describe the neutrophil lineage here.

Neutrophils are circulating leukocytes that ingest microorganisms and release granules containing enzymes that kill them. Human and mouse neutrophil markers (CSF3R^+^ and MSR1^–^) identified about 59,000 developing, proliferating and mature lemur neutrophils across the atlas that recapitulated their full trajectory (Fig. 2b). The trajectory showed sequential expression of granulopoiesis genes (Extended Data Fig. 6e), which mimicked the time course of different granule production during human neutrophil maturation^29^. It included antimicrobial enzymes absent or expressed at low levels in the corresponding mouse granules (*DEFA1*, *DEFA4*, *BPI*, *ALPL* and *ARG1*)^30^. Lemur neutrophils also expressed multiple human neutrophil genes missing in mice (*AZU1*, *IL32*, *TCN1*, *FCAR*, *S100A12*, *CCL14*, *CCL16* and *CXCL8*)^30^ (Extended Data Fig. 6e).

**Extended Data Fig. 7:**
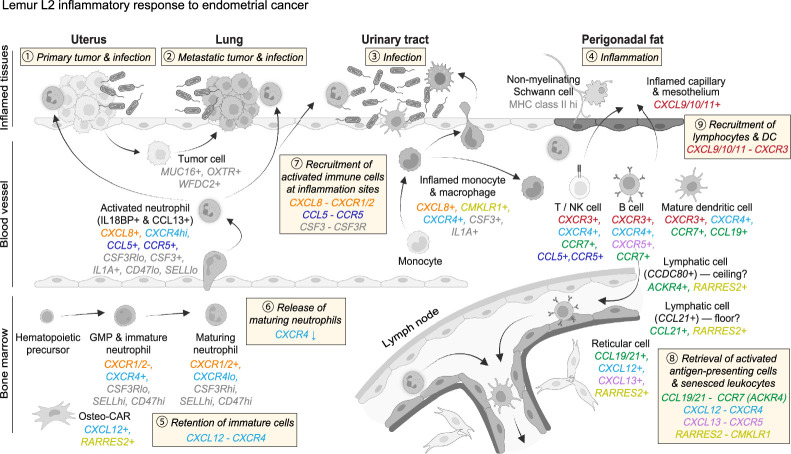
Control of immune cell development, activation, and senescence. Atlas-informed control of multi-organ tumor progression and the local and systemic inflammatory programs in L2, diagnosed with endometrial cancer (Step 1), metastatic spread to lung (2), secondary bacterial infection in both organs, plus suppurative cystitis (3) and suspected inflammation in perigonadal fat (4). Involved cell types in bone marrow (bottom left), circulating in blood vessels (middle), in the inflamed tissues (top), and in lymph nodes (bottom right) are shown along with their marker genes, and the signals (*ligand–receptor* pairs in tan boxes, colored as in Fig. 2a) proposed to control the local and systemic inflammatory steps (5-9) are indicated. Detailed description in Supplementary Note 5. Schematic created in BioRender. Ezran, C. (2025) https://BioRender.com/r8i9ddj.

Mapping tissue locations of neutrophils along the trajectory in each lemur (L1–L4) revealed specific inflammatory sites and global feedback regulation of haematopoiesis (Fig. 2c and Extended Data Fig. 7). The expected distribution of neutrophils in health (earliest progenitors and maturing neutrophils predominantly localized to bone marrow; mature, unactivated neutrophils enriched in blood and other tissues) was observed for lemur L4 (Fig. 2c). However, activated neutrophils (designated CCL13^+^ and IL18BP^+^, end of trajectory) were found in the lung, bladder, kidney and perigonadal fat of lemur L2, the lung of lemur L1 and the uterus of lemur L3 (Fig. 2c and Extended Data Fig. 6e–g),which were focal sites of inflammation from infection or malignancy (see below). These advanced neutrophils showed downregulation of mature neutrophil markers that facilitate extravasation, induction of chemokines that promote homing to inflammatory sites and recruitment of additional neutrophils (*CXCL8* (also known as *IL8*) and *CCL5* (also known as *RANTES*))^31^ and markers of neutrophil ageing and lymph node trafficking (Extended Data Fig. 6e,g and Supplementary Notes 5 and 6). The CCL13^+^ and IL18BP^+^ subtypes showed different tissue distributions across lemurs L1–L3 (Extended Data Fig. 6e–g and Supplementary Note 6), which suggested that local factors can drive distinct activation pathways. We also uncovered global responses to neutrophil activation. Lemur L2 had leukocytosis (32.1 k µl^–1^; 4.5–11 k µl^–1^ in healthy humans) dominated by neutrophils (91%), which were shifted towards immature stages of the trajectory (Fig. 2c). This result provides a molecular demonstration of the classical ‘left shift’ seen in smears of human blood, which reflects the movement of immature neutrophils from the bone marrow into the circulation to replenish neutrophils recruited to an infection site^32^ (Extended Data Fig. 7). Lemur L1 showed a distinctive global pattern, with neutrophils from across the trajectory in circulation (Fig. 2c), presumably from dysregulation of granulopoiesis by widespread fibrous osteodystrophy, as seen in the histopathology analyses.

**Extended Data Fig. 8:**
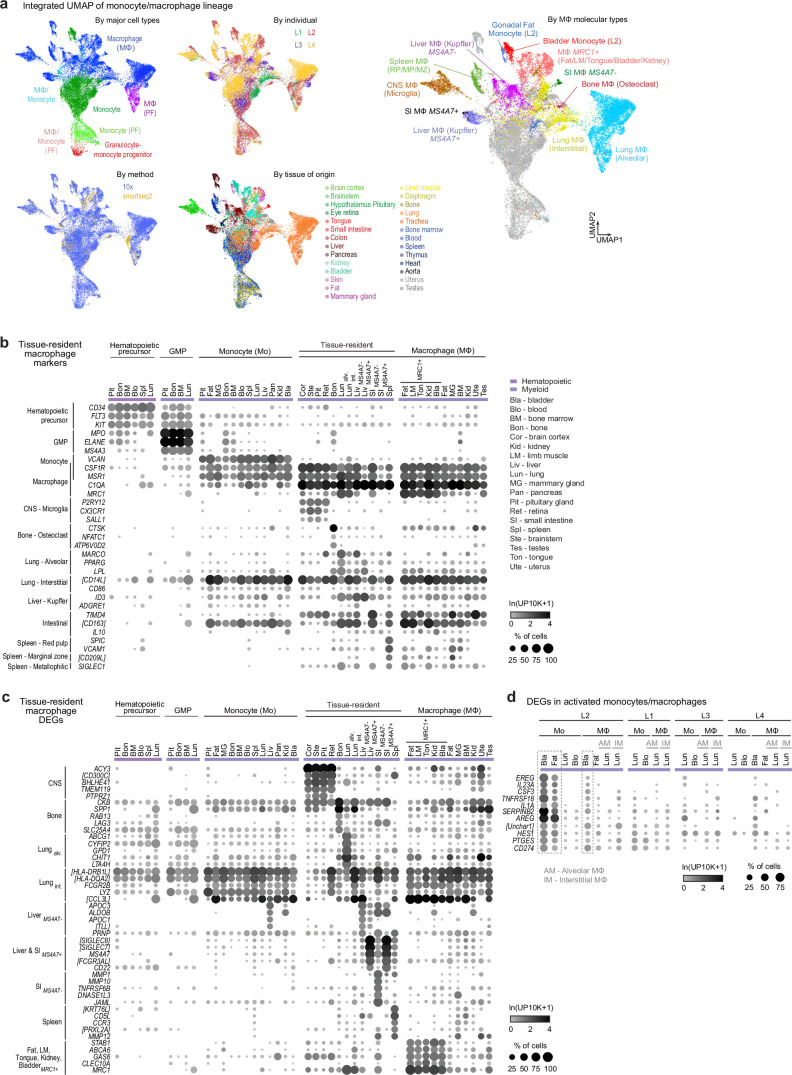
Monocyte and macrophage lineage development and tissue specialization. **a**. UMAP of atlas monocyte, macrophages, and their progenitors, integrated by FIRM across tissues and individuals (10x and SS2, L1-L4), colored by major cell types (top left), lemur individual (top middle), scRNA-seq method (bottom left), tissue source (bottom middle), and major groups of tissue-specific/resident macrophages (right). Note separate clustering of different macrophage subtypes as well as a unique population of activated monocytes (L2 bladder and perigonadal fat). **b**. Dot plot showing mean expression of classical marker genes for hematopoietic precursors, granulocyte-monocyte progenitors (GMP), monocytes, macrophage, and tissue-resident macrophage markers across monocyte/macrophage cell types and their progenitors, separated by tissue. Note some markers are shared between multiple cell types (see Supplementary Table 1 in the accompanying paper^1^). *[CD14L], LOC105862649; [CD163], LOC105869074; [CD209L], LOC105885453*. **c**. Dot plot showing mean expression across cell type as in b of top DEGs in the indicated tissue-resident macrophage populations compared to all other macrophage populations. *[CD300C], LOC105878881; [HLA-DRB1L], LOC105876782; [HLA-DQA2], LOC105869752; [CCL3L], LOC105882215; [TLL], LOC105872655; [SIGLEC8], LOC105866341; [SIGLEC7], LOC105882132; [FCGR3AL], LOC105873562; [KRT76L], LOC105871481; [PRXL2A], LOC105863040*. **d**. Dot plot showing mean expression of the top DEGs in the population of separately-clustered monocytes from L2 bladder and perigonadal fat compared to the other atlas monocytes/macrophages (blood, lung shown), separated by individual. DEGs include inflammation-associated genes *CD274/PD-L1*, *IL23A*, *AREG*, *CSF3*, *IL1A*, suggesting these could be activated populations. *[Uncharacterized 1], LOC105869025*. See Supplementary Note 7 for further analysis. Source data

We similarly mapped development and trafficking of the monocyte–macrophage lineage, which showed dozens of distinct, tissue-specific macrophage subtypes, including several locally activated subtypes (Extended Data Fig. 8, Supplementary Note 7 and Supplementary Fig. 4). By contrast, mature T cells, natural killer (NK) cells, natural killer T (NKT) cells and innate lymphoid cells formed a single isolated cluster, as did B cells and plasma cells (Fig. 2b and Extended Data Fig. 6h), which suggested rapid lymphocyte development with few standing intermediates.

Extended Data Fig. 7 summarizes the expression of the above highlighted chemokines and immune regulatory genes that govern the trafficking of leukocytes from the bone marrow into the circulation, extravasation into inflamed tissues and clearance through the lymphatics in response to cancer and infection in lemur L2. This analysis highlights how the organism-wide atlas provides a rich and dynamic portrait of the lemur immune system, revealing many cellular and molecular aspects of development and function, including human-like features that differ from mice.

## Lemur disease and physiology

**Fig. 3:**
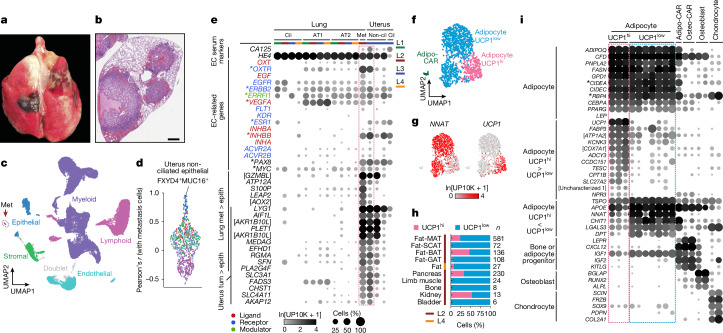
Cellular and molecular characterization of mouse lemur cancer and fat depots. **a**,**b**, Image of an intact lung from lemur L2 (**a**) and a section stained with haematoxylin and eosin (**b**) showing metastatic endometrial tumour nodules on the lung surface and extending into the parenchyma^8^ (*n* = 1). Scale bar, 1 mm. **c**, UMAP of lung cells from lemurs L1–L4 (10x and SS2 data, FIRM-integrated) coloured by compartment. Note the isolated cluster (arrow) of epithelial cells, identified as metastatic tumour (Met) cells. **d**, Sina plot of the Pearson’s correlation coefficients between lung metastatic cells from lemur L2 and all other atlas cell types (10x and SS2 data, coloured by compartment). Note the high correlation with uterine non-ciliated epithelial cells (FXYD4^+^MUC16^+^) from lemur L3, presumptively a primary tumour. **e**, Dot plot of the mean expression in lung and uterus epithelial cell types (separated by lemur, coloured bars) of endometrial (and ovarian) cancer (EC) serum marker genes and genes (indicated by an asterisk) known to be amplified, overexpressed or mutated in EC with their cognate ligands, receptors and/or modulators^38–42^. Lung met > epith, genes enriched in lung metastasis compared with lung epithelial cell types; Uterus tum > epith, genes enriched in uterine FXYD4^+^MUC16^+^ cells compared with other uterine epithelial cell types. **f**,**g**, FIRM-integrated UMAP of adipocytes and adipo-CAR cells (10x and SS2 data) coloured by cell type (**f**) and expression levels of indicated genes (**g**). Adipocytes form two main populations, distinguished by the expression of classical white (for example, *NNAT*) and brown (for example, *UCP1*) adipocyte markers (**g**), and designated here as UCP1^low^ and UCP1^hi^, respectively. UCP1^low^ formed two subclusters in UMAP that differed only in the total gene and UMI counts per cell and not the expression of any biologically significant genes (Extended Data Fig. 10a–c). **h**, Distribution of UCP1^hi^ versus UCP1^low^ adipocytes in the indicated fat depots and organs (10x and SS2 data from lemur L2 and combined fat depots from lemur L4). *n*, number of adipocytes. BAT, interscapular brown adipose tissue; GAT, perigonadal adipose tissue; MAT, mesenchymal adipose tissue; SCAT, subcutaneous adipose tissue. **i**, Dot plot of the mean expression of the indicated cell-type markers and differentially expressed genes in the indicated cell types (L1–L4, 10x data). Notably, the classical brown adipocyte marker *CIDEA* and the white adipokine *RBP4* (asterisks) were equally expressed across all adipocytes. Symbols in brackets indicate the description of genes identified by NCBI as loci: [*GZMBL*], *LOC105864431*; [*AOX2*], *LOC105856978*; [*AKR1B10L*], *LOC105857399* and *LOC105860191*; [*ATP1A2*], *LOC105862687*; [*COX7A1*], *LOC105876884*; [Uncharacterized 1], *LOC105854963*. See also Extended Data Figs. 9 and 10. Cil, ciliated; Met, metastatic; Non-cil, non-ciliated. Source data

**Extended Data Fig. 9:**
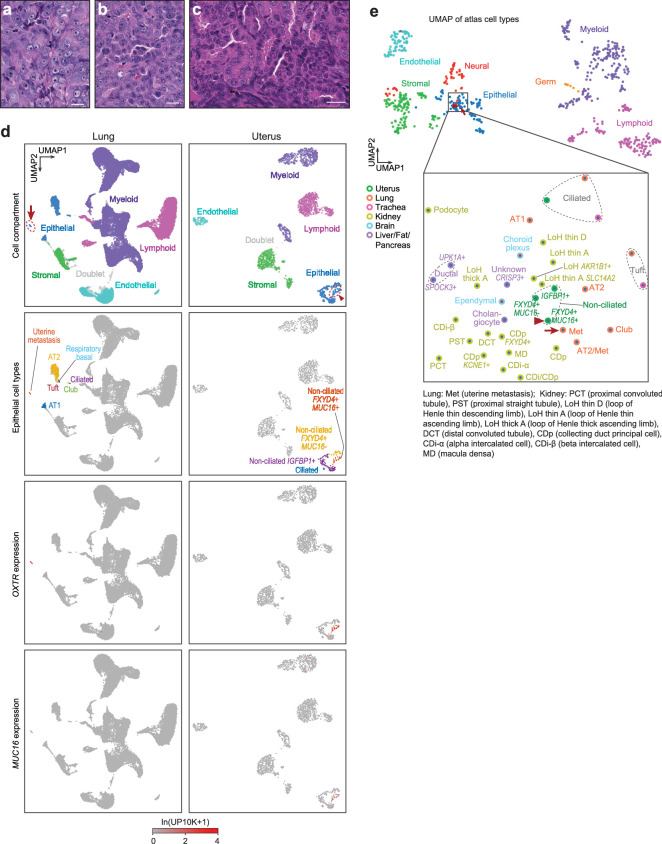
Further characterization of uterine tumor cells and metastasis to lung. **a-c**. H&E sections of primary endometrial tumor cells in the uterus of L2 (a, N = 2), which metastasized to the lung (b, close up of Fig. 3b, N = 1), and of endometrial tumor cells of L3 (c), which metastasized locally. Scale bar, 20 μm (all panels). Full set of micrographs are available on Tabula Microcebus web portal. **d**. UMAP of lung cells (left, L1-L4, 10x and SS2) and uterine cells (right, L3, 10x) integrated by FIRM and colored by (top to bottom) compartment, epithelial cell type designation (non-epithelial cells in grey), expression level of uterine marker *OXTR*, and expression level of human endometrial cancer marker *MUC16/CA125*, respectively. Note isolated cluster of lung epithelial cells (arrow, from L2), identified as metastatic tumor cells and cluster of uterine epithelial cells (arrowhead, from L3), designated as non-ciliated epithelial cell of uterus (*FXYD4*+ *MUC16*+), presumed to be primary endometrial tumor cells. **e**. UMAP of all atlas cell types showing their mean transcriptional similarity (L1-L4, 10x)^1^. Each data point is a cell type (unique combination of tissue and free annotations), colored by compartment. Boxed inset shows close-up of indicated UMAP region with cell types colored by compartment (circle fill) and tissue of origin (circle border), and labeled by cell type designation. Note metastatic tumor cells (Met, arrow) from L2 lung and adjacent (molecularly similar) uterine non-ciliated epithelial cells (*FXYD4*+ *MUC16*+, arrowhead) from L3, supporting uterine origin of the metastatic tumor. Source data

We leveraged the atlas to explore lemur disease and physiology. The analysed lemurs were elderly and had human-like pathologies, as revealed by necropsy^8^. Both female lemurs (L2 and L3) had endometrial cancer (Fig. 3a,b and Extended Data Fig. 9a–c). This cancer is the most common malignancy of the female reproductive tract and the fourth most common cancer in women in the United States^33^, with increasing incidence and mortality attributed to an ageing population and increasing obesity^34^. Animal models of this cancer are limited. Mice do not naturally acquire endometrial cancer, and although rats do, they and engineered mouse models generally resemble low-grade type 1 rather than high-grade, intractable type 2 tumours^35^. The cancer in lemur L2 was uncovered as a previously undescribed lung cell type (Fig. 3c) that expressed high levels of *OXTR* (which encodes the oxytocin receptor) (Fig. 3e), a gene known to be highly expressed in female reproductive tissues^36^. Comparisons across the atlas revealed their similarity to uterine epithelial cells (Fig. 3d and Extended Data Fig. 9d,e), and necropsies established the diagnosis of primary endometrial carcinoma with metastases to the lung (L2) or to the mesenteric lymph nodes (L3)^8^. Organism-wide atlases therefore enabled the identification of the primary site of cancers of unknown origin, which constitute around 2% of all human cancers^37^.

The presumptive primary tumour cells in the uterus of lemur L3 (based on co-expression of the human endometrial and ovarian cancer markers *CA125* (also known as *MUC16*) and *HE4* (also known as *WFDC2*)^38^), showed enriched expression of *OXTR*. *MYC* and *ERBB2* (also known as *HER2*) (Fig. 3e), two genes commonly amplified or mutated in human type 2 endometrial tumours^39^, were also enriched. Moreover, the cells expressed *INHBB*, which, as a homodimer (activin B), promotes cancer cell migration and invasion, and its expression correlates with higher grade endometrial tumours^40^. The lung metastasis sample also expressed *ERBB2*, its binding partner *EGFR* and specifically the ligand *EGF*, which indicated progression to autocrine mitogenic signalling during metastasis. Expression of *ESR1* (which encodes the oestrogen receptor) was lost^41^ (Fig. 3e), a pattern that correlates with more advanced human tumours^42^. Lemur endometrial cancer therefore molecularly and histologically mimics the aggressive human form, including its metastatic propensity. However, experimental validation is needed. The lemur presents a promising model to explore susceptibility factors, pathogenetic mechanisms and therapies, in particular anti-angiogenic (VEGFR), anti-EGF–EGFR and endocrine (for example, ESR1) therapies given the expression of these potential targets in both lemur and human tumours. Conversely, therapies used in humans might help control the disease in lemurs^43^.

**Extended Data Fig. 10:**
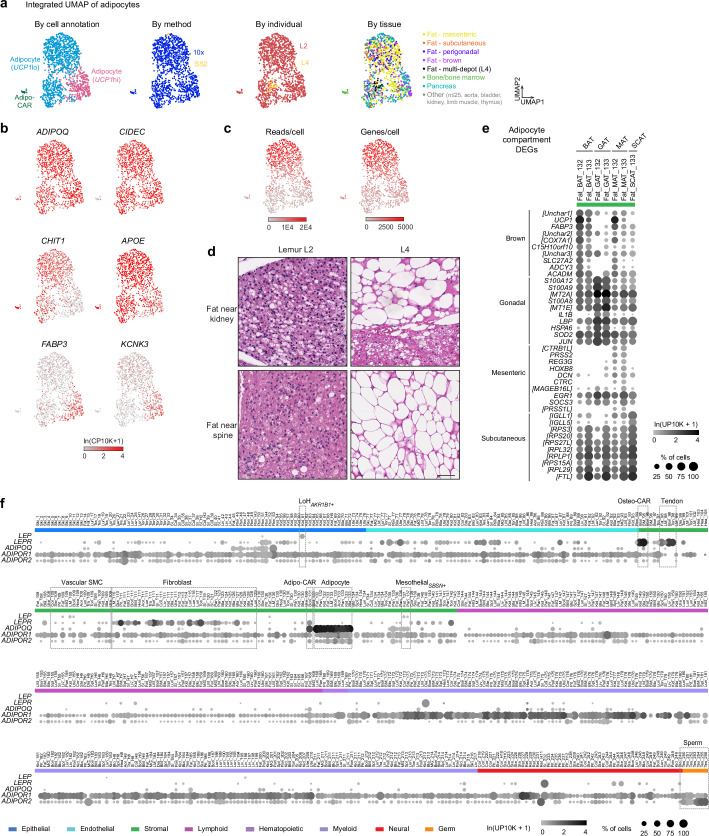
Further characterization of lemur adipocytes and their expression patterns. **a**. FIRM-integrated UMAP of adipocytes and adipo-CAR cells (10x and SS2) as in Fig. 3f with cells colored by (left to right) cell type designation, scRNA-seq method, individual lemur source, and tissue source, respectively. **b**. UMAP as above colored by expression level of indicated adipocyte markers (*ADIPOQ*, *CIDEC*) and example DEGs in *UCP1*lo (*CHIT1*, *APOE*) and *UCP1*hi (*FABP3*, *KCNK3*) adipocytes. **c**. UMAP as above colored by the number of scRNA-seq reads per cell (UMIs, 10x; transcripts, SS2, left) and number of genes detected per cell (right). Note the heterogeneity of *UCP1*lo population, which forms two subclusters distinguished by total read per cell and genes detected per cell, but not by any biologically significant DEGs. **d**. H&E-stained sections of fat tissues from L2 (left) and L4 (right) that are near the kidney (top) and paraspinal muscle (bottom), N = 4. Scale bar, 50 μm (all panels). Full set of micrographs available online on Tabula Microcebus web portal. **e**. Dot plot of mean expression of the top 10 DEGs in each of the four fat depots: BAT, interscapular brown adipose tissue; GAT, perigonadal; MAT, mesenchymal; SCAT, subcutaneous (L2, 10x). *[Uncharacterized 1], LOC105856764; [Uncharacterized 2], LOC105867540; [COX7A1], LOC105876884; [Uncharacterized 3], LOC105867541; [MT2A], LOC105866476; [MT1E], LOC105866554; [CTRB1L], LOC105875474; [MAGEB16L], LOC105877758; [PRSS1L], LOC105873340; [IGLL1], LOC109729893; [IGLL5], LOC105882024; [RPS3], LOC105862350; [RPS20], LOC105874908; [RPS27L], LOC109731171; [RPL32], LOC105861123; [RPLP1], LOC105859117; [RPS15A], LOC105857549; [RPL29], LOC105863618; [FTL], LOC105870251*. **f**. Dot plot of expression of adipokines *LEP* and *ADIPOQ* as well as their receptors across atlas cell types (L1-L4, 10x). Note abundant and specific expression of *ADIPOQ* but lack of *LEP* expression in adipocytes. Curiously, *LEP* transcripts are detected in the *AKR1B1*+ kidney loop of Henle cells (LoH), mesothelial cells, and some vascular-associated smooth muscle cells (SMC), although at very low levels. In contrast, *LEPR* shows expected expression in various cell types including tendon cells, fibroblasts, and endothelial cells, and high *LEPR* expression is found in mesenchymal progenitor cell types such as osteo-CAR and adipo-CAR cells. Also note ubiquitous expression of *ADIPOR1* across almost all atlas cell types, and enriched expression of *ADIPOR2* in sperm lineage cells and adipocytes^41^. Source data

A notable aspect of mouse lemur physiology is their marked annual oscillations in body weight, temperature and metabolism as they enter a hibernation-like (torpor) state during the resource-poor winter. Mouse lemurs therefore provide a model for primate seasonal rhythms, regulation of metabolism and adipose biology^4,44^. We analysed four lemur fat depots and identified hundreds of adipocytes that expressed canonical adipocyte markers, including lipid biosynthesis and metabolic genes (for example, *PNPLA2*, *FASN*, *GPD1* and *CIDEC*) and adipokines (*ADIPOQ* and *CFD*)^45^ (Fig. 3f–i and Extended Data Fig. 10). We also found rare adipocytes in seven other tissues (Fig. 3h and Extended Data Fig. 10a,d).

Lemur adipocytes showed two notable features. Although they expressed most of the established adipocyte markers, they did not strongly express the classical adipocyte hormone leptin (*LEP*), which is highly expressed by human and mouse adipocytes and regulates food intake, energy expenditure and weight^46^ (Extended Data Fig. 10f). *LEP* expression was detected in only 0.6% of lemur adipocytes and at a low level (mean of 3.2 transcripts per 10,000 reads), and even lower levels in unrelated cell types. However, its receptor *LEPR* was selectively and highly expressed in a similar cellular pattern as in humans and rodents^41,46^ (Extended Data Fig. 10f). Perhaps *LEP* is inducible in lemur adipocytes depending on the season, diet or other factors^47^, or some occult cellular source (or another gene) has usurped its function.

Another aspect of note was the blurring of the distinction between white and brown adipocytes. Aside from bone adipo-CAR cells, which may be adipogenic progenitors (Fig. 3f,i), adipocytes formed two continuous populations distinguished by the expression of uncoupling protein 1 (*UCP1*), the canonical thermogenic brown adipocyte marker^48^ (Fig. 3f,g and Extended Data Fig. 10a–c). We designate the UCP1^hi^ population as ‘brown-like’ because they also expressed increased levels of known thermogenesis regulators (for example, *CPT1B*, *SLC27A2*, *FABP3* and *KCNK3*)^49,50^ (Fig. 3i). We designate the UCP1^low^ population as ‘white-like’ because of the enriched expression of many white adipocyte genes (for example, *NNAT* and *DPT*), despite the expression (albeit low) of the brown-defining gene *UCP1*. Further blurring the white–brown distinction, both the classical brown adipocyte marker *CIDEA* and the white adipokine *RBP4* were equally expressed across all adipocytes^51,52^ (Fig. 3i). These mixed molecular signatures suggest that the white–brown adipocyte distinction is less strong in *M.* *murinus*, and the continuum between them suggests potential interconversion between white-specific lipid storage and brown-specific thermogenesis, perhaps affording functional plasticity for energy-intensive seasonal cycling.

There was no exclusively brown-like fat depot among the surveyed depots; each site contained exclusively white-like adipocytes or a mix (Fig. 3h). Different depots did not cluster separately or differentially express any biologically significant genes (Extended Data Fig. 10a,e), except gonadal adipocytes, which were enriched for *S100A8*, *S100A9*, *S100A12*, *IL1B*, *MT2A* and *MT1E*, which are correlated with inflammatory and feeding status and insulin resistance^53,54^. It will be important to study seasonal changes in gene expression in each depot to explore adipocyte plasticity and its role in seasonal physiology.

## Primate genes missing in mice

**Fig. 4:**
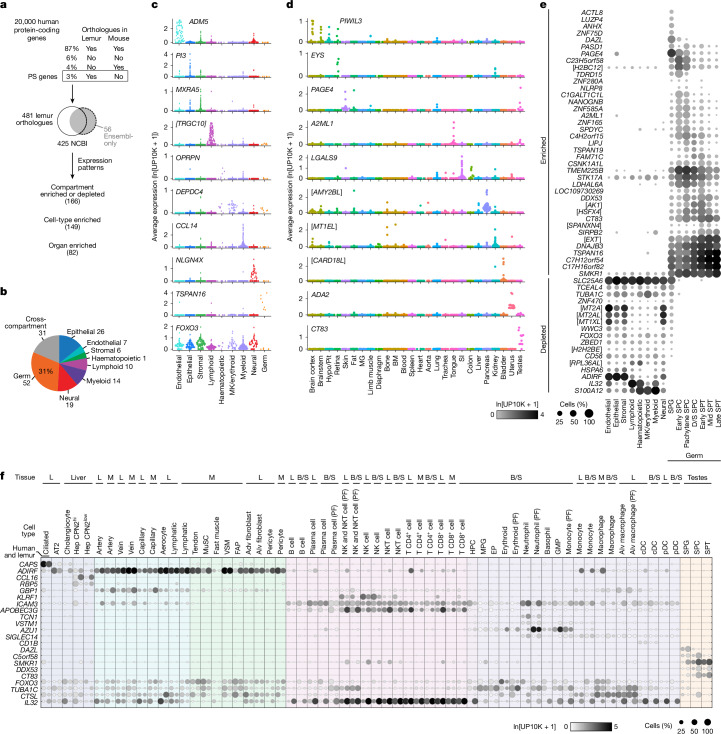
Lemur expression patterns of PS genes. **a**, Scheme for identifying and characterizing PS genes. The pie chart shows the fraction of the approximately 20,000 human protein-coding genes with identified orthologues (in NCBI, Ensembl and/or Mouse Genome Informatics) in lemur and/or mouse. The 539 (3%) that share an orthologue only with lemur (PS genes) correspond to 481 lemur genes **b**, Number of PS genes enriched (or depleted) in a specific tissue compartment. Cross-compartment, enriched or depleted in >1 compartment. **c**,**d**, Sina plots showing the expression of example PS genes that are compartment enriched or depleted (**c**) or organ-enriched (**d**) (10x data), with cell types (dots) grouped by compartment (**c**) or by organ (**d**). **e**, Dot plot of the mean expression of PS genes enriched or depleted in the germ compartment. Values are averaged across all cells in the indicated non-germ compartments and germ cell types (10x data). **f**, Dot plot of the mean expression of selected PS genes in 63 orthologous cell types in human and lemur lung (L), skeletal muscle (M), liver, testes, and bone marrow and spleen (B/S)^1^. Rows, orthologous genes (indicated with human gene symbols). Columns, cell types displayed as paired dots showing expression in humans and lemurs. Symbols in brackets indicate the description of genes identified by NCBI as loci: [*TRGC10*], *LOC105878255*; [*AMY2BL*], *LOC105863954*; [*MT1EL*], *LOC105866478*; [*CARD18L*], *LOC105862464*, [*H2BC12*], *LOC105858749*; [*AK1*], *LOC105869668*; [*HSFX4*], *LOC109730266*; [*SPANXN4*], *LOC105864720*; [*EXT*], *LOC105877793*; [*MT2A*], *LOC105866476*; [*MT2AL*], *LOC105866477*; [*MT1XL*], *LOC105866553*; [*H2H2BE*], *LOC105865505*; [*RPL36AL*], *LOC105873222*. D/S, diplotene/secondary; EP, erythroid progenitor; Hep, hepatocyte; MG, mammary gland; MGP, megakaryocyte progenitor; MK, megakaryocyte; MuSC, skeletal muscle stem cell; VSM, vascular smooth muscle. See also Supplementary Figs. 5 and 6. Source data

Lemurs could be valuable in the study of human genes missing or expressed differently^1^ in mouse or other model organisms. Comparisons of lists of orthologous protein-coding genes in humans, lemurs and mice (Supplementary Table 8) identified 539 human genes for which there were orthologues in lemur (425 orthologues annotated in NCBI) but not mice (Fig. 4a and Supplementary Table 9), which we call PS (primate-selective) genes here for simplicity. At least 24 PS genes cause human disease or phenotypes^55^ (Supplementary Table 9), whereas others have important roles in human physiology, such as motilin (*MLN* and the receptor *MLNR*) in gastrointestinal motility^56^, *CD58* in antigen presentation, and *FCAR* (IgA receptor), *CXCL8* and *S100A12* in inflammation. Gene set enrichment analysis showed that PS genes are enriched in transcription factor activity and regulation and in herpes simplex virus 1 infection, including many zinc finger proteins (Supplementary Table 10). Nearly all (94%) NCBI-annotated PS genes were expressed in the atlas (Supplementary Table 9). Some were selectively expressed (or depleted) in specific compartments (166 genes) and/or specific organs (99 genes) (Fig. 4a–e, Supplementary Fig. 5). Many were specific to the male germline, immune cells and neurons, which indicated substantial evolutionary gene plasticity in these compartments. Many PS genes (including some with unknown functions) exhibited similar expression patterns in humans and lemurs (Fig. 4f, Supplementary Table 9 and Supplementary Fig. 6), and these should be prioritized for functional study in lemurs.

## Phenotyping natural mutations

**Fig. 5:**
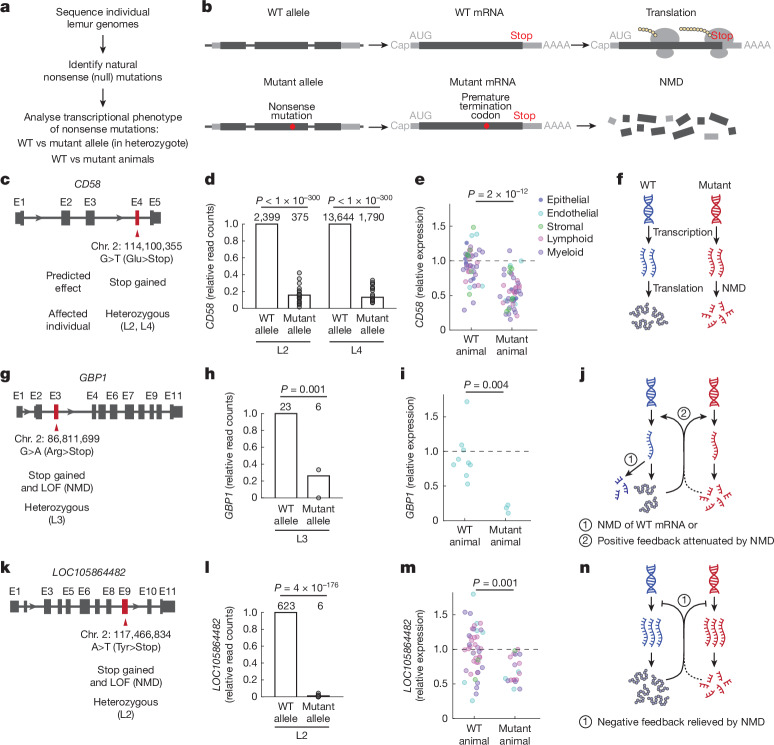
Nonsense mutations in lemur immune genes and their transcriptional phenotypes. **a**, Scheme for finding and transcriptional phenotyping of nonsense mutations in the profiled lemurs. **b**, NMD pathway showing the degradation of mRNA with a nonsense mutation (bottom) but not the corresponding WT mRNA (top). **c**–**n**, Identified heterozygous nonsense mutations and their transcriptional consequences for three lemur immune genes present in lemur and human genomes but missing in the mouse genome: *CD58* (**c**–**f**), a ubiquitously expressed CD2-binding T cell activator; *GBP1* (**g**–**j**), an interferon-inducible GTPase highly expressed in endothelial cells; and *LOC105864482* (*PYH1N1* homologue; **k–n**), an interferon-inducible protein abundant in T cells and NK cells. **c**,**g**,**k**, Diagram of mutations (arrowhead) with the affected exon (E) in red in the affected (heterozygous mutant) individual lemurs. ‘Stop’ indicates a change to a stop codon in the mutant allele. **d**,**h**,**l**, Bar plots of relative transcript read counts in the mutant allele normalized to counts from the WT allele (raw values above bars) for each affected individual (10x data). Dots, each tissue. Note that transcript reads analysed here are only those that covered the mutation position. *P* values, one-tailed binomial test (combining reads from all tissues). Sample size (unique read count) indicated above the bar. **e**,**i**,**m**, Dot plots of the relative expression levels of the gene in mutant (heterozygous) versus WT individuals, normalized to the mean expression level across all WT cells (dashed line). Dots, cell types separated by each individual, coloured by compartment (*n* = 46, 49 (**e**); 9, 3 (**i**); 44, 19 (**m**) for WT and mutant, respectively). *P* values, two-tailed student *t*-test. **f**,**j**,**n**, Models of the effects of the nonsense mutation on the expression of the mutant and WT alleles of the gene. **f**, Simple model showing how NMD degrades only the mutant and not the WT transcript. Around 90% depletion of *CD58* mutant transcript (**d**) results in about 45% less transcripts in heterozygous mutants (**e**). **j**, NMD destroys both mutant and WT transcripts (or, there is attenuation of a positive-feedback loop). Thus heterozygous mutants have a reduction in total *GBP1* transcripts (**i**) greater than expected (**h**) from the simple model. **n**, NMD destroys mutant transcripts, but the gene exhibits compensatory transcriptional upregulation. Despite almost complete (99%) elimination of mutant transcripts (**l**), heterozygotes show only about 30% less total gene transcripts than WT animals (**m**). See also Extended Data Fig. 11. LOF, loss of function. Source data

**Extended Data Fig. 11:**
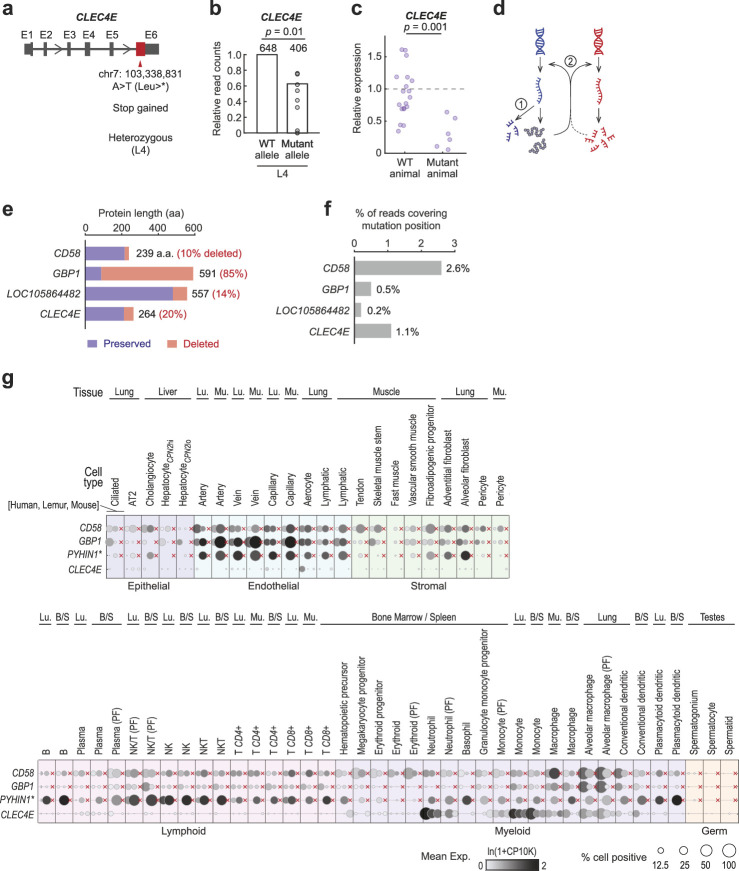
Further characterization of the identified nonsense mutations identified in the profiled lemurs. **a-d**. Gene schematic (**a**), bar plot (**b**) and dot plot (**c**) of gene expression, and model of NMD regulation of the gene’s expression (**d**) as in Fig. 5c–n but for *CLEC4E*, a fourth gene with a nonsense mutation identified in a lemur in the atlas (L4). *CLEC4E* is an innate immune regulator expressed in neutrophils and monocytes. (**c**) N cell types = 19, 6 for wildtype and mutant, respectively. **e**. Length of preserved and C-terminus depleted portion of the mutant protein predicted for the four characterized genes with a nonsense mutation, based on the position of the nonsense mutation in the gene. Numbers at right of bar indicate the total length of the protein (black) and the percent of the depleted portion (red). **f**. Percent of transcript reads that cover the mutation position among all transcripts reads that align to the corresponding gene in the affected individual (10x). **g**. Dot plot showing mean expression of the four characterized genes across 63 orthologous cell types in human, lemur, and mouse^1^. Rows are orthologous genes, indicated by their respective human gene symbols. *, lemur homologue for human gene *PYHN1* is *LOC105864482*. Columns are cell types, displayed as trios of dots showing the respective expression, from left to right, in human, lemur, and mouse. Red cross, gene missing in mouse genome. Source data

A crucial step in establishing a model organism is the development of methods for functional analyses in vivo of individual genes and mutations. We used the atlas to achieve this for lemurs (Fig. 5a and Extended Data Fig. 11). Whole-genome sequencing was performed, and natural mutations (single nucleotide polymorphisms and insertions and deletions) in the profiled lemurs were uncovered by carrying out comparisons to the reference genome Mmur 3.0. We focused on genes with putative null (nonsense) alleles that were present in one or two of the profiled lemurs.

Most identified nonsense mutations were heterozygous; therefore, we leveraged our scRNA-seq data to distinguish transcripts from each allele to quantify the effect of nonsense-mediated mRNA decay (NMD) (Fig. 5b). For autosomal genes, both alleles are generally transcribed at similar levels. But in an individual with a heterozygous nonsense mutation, transcripts with the mutation would be selectively degraded by the NMD pathway and hence underrepresented relative to the wild-type (WT) transcript, with the magnitude of difference reflecting the efficiency of mutant mRNA destruction.

Here we describe the transcriptional phenotypes of nonsense mutations identified in four genes for which human orthologues function as immune regulators (Fig. 5c–n and Extended Data Fig. 11a–d). Two are PS genes: *CD58* (which encodes a T cell CD2 ligand) and *GBP1* (which encodes an interferon-inducible GTPase in innate immunity). The third, *LOC105864482*, is a homologue of human *PYH1N1* (which encodes an interferon-inducible protein), with orthologues restricted to primates (and flying lemurs, a close primate relative). For all three genes, nonsense transcript reads were substantially depleted (74–99%) compared with WT transcripts in the same individual, which implied efficient destruction by NMD (Fig. 5d,h,l). For the fourth gene, *CLEC4E* (which encodes an immune regulator conserved across humans, lemurs and mice), mutant transcript reads were 37% depleted, which implied less efficient NMD (Extended Data Fig. 11b). This result is consistent with the location of this mutation in the last exon, which prevents or reduces NMD^57^.

We used the atlas to reveal the indirect consequences of the mutations on expression of the WT allele and overall expression of the gene by comparing total transcript levels of the gene between heterozygous and WT individuals. For *CD58*, heterozygous individuals exhibited about 45% less *CD58* expression than WT individuals (Fig. 5e), the level expected based on the observed approximate 90% depletion in the mutant transcript (Fig. 5d). This finding indicates that transcription of the WT allele was unaffected by the transcripts with nonsense mutations (Fig. 5f). However, for *LOC105864482*, the heterozygous lemur showed only an approximately 30% overall reduction in *LOC105864482* expression relative to WT individuals (Fig. 5m) despite almost complete (99%) elimination of the mutant transcript (Fig. 5l). This result suggests that there is compensatory upregulation of the WT transcript (Fig. 5n). By contrast, *GBP1* and *CLEC4E* heterozygotes showed more than the expected reduction in their overall expression (Fig. 5h,i and Extended Data Fig. 11b,c), which suggested that NMD somehow also reduces (in *trans*) transcripts of the respective WT allele or positive feedback is attenuated (Fig. 5j). Thus, the atlas enabled the transcriptomic characterization of nonsense mutations in lemurs and highlighted gene-specific differences in NMD.

## Discussion

We used our transcriptomic atlas^1^ to establish a foundation for molecular and genetic studies of mouse lemurs. We identified and named thousands of mouse lemur genes and their expression homologues in addition to hundreds of thousands of splice forms missed by conventional pipelines, including genes in the most difficult to annotate loci. We also showed how the atlas can be used to elucidate lemur physiology with cellular and molecular precision, such as development, trafficking and activation of immune cells, as well as lemur endocrinology^41^. By combining the atlas with clinical metadata and histopathology, we ascertained rich molecular portraits of lemur disease such as the pathogenic sequence of endometrial cancer. Such ‘molecular cell autopsies’ represent a new era of pathology, providing both local and systems-level understanding of disease and inflammatory processes.

We also used the atlas to identify high priority areas for mouse lemur studies, in particular genes, physiology and diseases that are conserved in humans, or specific to lemurs, but absent or divergent in mice. For example, further investigation into primate-specific molecular features of the immune program, adipocytes and metastatic endometrial cancer is needed. Our classical autopsies uncovered other human-like pathologies, including cataracts, osteoarthritis, chronic kidney disease and amyloidosis^8^, and previous studies have identified Alzheimer’s-like neurodegenerative disease^5^. A top priority for futures studies are the >400 primate genes missing in mice, and the many others present in mice but for which expression^1^ or splicing differ from primates.

Finally, our experimental pipeline for reverse genetic analysis transcriptomically characterized natural null alleles in several top priority genes: primate immune genes missing in mice. In parallel, forward screens for lemur morphological, physiological and disease phenotypes identified eight human-like cardiac arrhythmias and mapped the disease gene for one (sick sinus syndrome), a transporter with primate-specific pacemaker function^58^. Forward and reverse genetic approaches are now possible for the mouse lemur, although tools for targeted genetic and transgenic studies (for example, induced pluripotent stem cells, CRISPR technologies and viral vectors) await development.

The results from this study and the accompanying paper^1^ have created a strong molecular, cellular and genetic foundation that make mouse lemurs a tractable primate model. This foundation and our approaches can be used to elucidate almost any aspect of primate physiology, disease, ecology and evolution, and can be compared to humans and mice as well as other emerging model organisms and ultimately the full tree of life.

## Methods

### uTAR analysis to identify unannotated genes

To uncover uTARs, we used a previously published workflow^11^ for scRNA-seq data that identifies TARs, genome regions with abundant transcript alignments. In brief, all mouse lemur 10x datasets were aligned to the genome assembly Mmur 3.0 using STAR with default parameters, without gene annotation indexing. Transcribed regions were predicted using groHMM^59^. TARs within 500 bp of another were combined into a single TAR and kept if they were expressed in at least 2 cells of the 10x atlas dataset. The detected TARs were then separated into aTARs and uTARs on the basis of whether the region is currently annotated as a gene in the NCBI annotation release 101 of Mmur 3.0. This strategy identified that aTARs and uTARs cover 284 and 42 Mbp, respectively, of the mouse lemur genome (2,487 Mbp).

To filter out transcriptional and sequencing noise from biologically significant uTARs, we then examined whether a uTAR was differentially expressed across cell types using Wilcoxon rank-sum tests. This analysis was performed separately for each tissue and individual lemur. A DE uTAR was defined as a uTAR if it met the following criteria: had a significant Bonferroni-corrected *P* < 0.05 from the two-tailed Wilcoxon rank-sum test; expressed in ≥25% of cells of a cell type; and had a cell-type mean expression level of ≥1.65 (*e*^0.5^) times the average of other cell types in the same tissue. Some of the uTARs passed the differential expression test in multiple cell types and/or tissues. Together, a total of 4,003 DE uTARs were identified. To infer their identity, we applied BLASTn on each of the DE uTARs against the nucleotide collection (nt) database (with a threshold of maximum *e* value of 0.01 and a minimum bit score of 50) using either the entire length of the uTAR or the peak coverage region (full width at half maximum region around the absolute peak in coverage after Gaussian smoothing in the uTAR location). Occasionally, multiple uTARs aligned to the same gene in another species. The genome location and inferred homology of all DE uTARs and their expression levels across the cells in the atlas (10x data) are provided in Supplementary Tables 1 and 2. There were 30 DE uTARs with a BLASTn result that corresponded to one of the 2,060 human genes without a mouse lemur orthologue, which are probably genes missed from annotations of Mmur 3.0 in the NCBI and Ensemble databases (for example, *GSTA3* in tendon cells of the bone, *TIGD1* in CD4^+^ T cells and *SPRR2G* in suprabasal epidermal cells; Supplementary Table 1). Note that 14 out of these 30 genes have no mouse orthologue, which suggests that these are PS genes.

DE uTARs were also classified as protein-coding or non-coding using the custom program Nf-core/predictorthologs (https://github.com/czbiohub-sf/nf-predictorthologs)^60^, with sequences containing 95% or more *k*-mers (*k* = 9) matching a reference database of mammalian proteins from UniProt assigned as putatively protein-coding, and otherwise assigned as non-coding. Coding sequences were then annotated using DIAMOND blast^61^ and non-coding sequences were annotated using Infernal cmscan (https://www.ebi.ac.uk/Tools/rna/infernal_cmscan/), both algorithms that are built into Nf-core/predictorthologs. Some uTARs had both coding reads and non-coding reads, which potentially represent incompletely spliced transcripts or untranslated regions.

To detect the developmental trajectory of the sperm lineage cells in the testes 10x dataset using the uTAR expression data, we followed the same procedure as for detecting the trajectory through gene expression data, which is described in the accompanying paper^1^. The analysis included a total of about 50,000 uTARs that have transcript reads in the testis dataset. Each uTAR was treated as a gene, and data were first normalized for scRNA-seq library size (to 10,000 total uTAR transcripts per cell) and natural log-transformed. The top highly variable uTARs (around 1,500) were then used for principal component analysis. The top 20 principal components that were not driven by extreme outlier data or immediate early genes were used to construct a two-dimensional (2D) UMAP using cell–cell Euclidean distances as input. The pseudotime developmental trajectory was then identified as the density ridge of the data in this 2D UMAP through automated image processing. Cells were assigned to the trajectory on the basis of the shortest connecting distance. The pseudotime trajectory coordinates of the cells were linearly normalized such that the trajectory started at 0 and ended at 1, and then were compared with the pseudotime coordinates derived from the gene-based trajectory using Pearson’s correlation. The uTAR expression data were similarly pre-processed (normalized, scaled and UMAP embedded) in other analyses, including when comparing the UMAP cell distribution patterns of the 10x colon dataset (Extended Data Fig. 1d) and in the silhouette coefficient analysis (Extended Data Fig. 1a).

To measure the consistency of cell distribution patterns for 10x datasets embedded in a UMAP using the gene, aTAR and uTAR expression spaces, silhouette coefficient values were calculated for each dataset (separated by tissue and individual lemur, and sequencing channel). Cells were grouped according to cell-type designation (free annotation). The silhouette coefficient value for each cell *i* in a dataset was calculated as *s*(*i*) = (*b*(*i*) – *a*(*i*))/max{*b*(*i*),*a*(*i*)}, where *a*(*i*) is the mean in-group distance (mean distance of cell *i* to the other cells in the same cell type) and *b*(*i*) is the minimal out-group distance (minimal distance of cell *i* to any cell in the tissue of a different cell type). The cell silhouette coefficient values were then averaged across each dataset to derive the dataset-averaged silhouette value, which is an overall score representing how well each cell type co-clusters and separates from other cell types in the UMAP embedded space, with higher positive values representing better separation. The silhouette coefficient values were calculated separately using the cell-to-cell distances in UMAPs based on the gene expression, aTAR expression and uTAR expression spaces, and then compared with a box plot (Extended Data Fig. 1a).

The lists of genes for each category shown in Extended Data Fig. 1f were obtained as follows. Lists of the top *n* variable NCBI-annotated genes were derived by applying variance-stabilizing transformation to the entire 10x dataset and selecting the genes with top *n* transformed variance. The list of the genes annotated by Ensembl only (not by NCBI) were detected by comparing the genomic positions of mouse lemur genes annotated in the two databases, searching for the Ensembl-annotated gene with no overlap in NCBI. The PS genes were derived as described below and listed in Supplementary Table 9.

To determine the amount of transcriptomic sequence information provided by the mouse lemur scRNA-seq atlas datasets, we estimated the total number of sequenced base pairs that mapped to the mouse lemur genome. For the 10x datasets, the total number of aligned paired-end reads (around 1.63 × 10^10^) was multiplied by the number of base pairs per read (90), which equated to 1.46 × 10^12 ^bp, although this number is inflated by PCR duplicates. However, summing the number of UMIs across all cells in the atlas (around 1.22 × 10^9^) and multiplying it by the number of base pairs per read (90) equated to 1.10 × 10^11 ^bp, which is 10-fold less than the estimate with total reads. For the SS2 datasets, the total number of aligned paired-end reads (1.11 × 10^10^) was multiplied by the number of base pairs per read (100), which equated to 1.11 × 10^12 ^bp (unique reads are not possible to identify in SS2 datasets). By comparison, the total number of bulk RNA-seq base pairs used by the NCBI for gene prediction and annotation of Mmur 3.0 equates to about 3.4 × 10^11 ^bp (across around 3.4 × 10^9^ total reads), which was calculated by summing the number of sequenced base pairs for each RNA-seq run (all tissues and generic samples included), information obtained from the Sequence Read Archive (SRA) (biosample identification numbers and corresponding SRA link available at https://www.ncbi.nlm.nih.gov/genome/annotation_euk/Microcebus_murinus/101/). However, this estimate is inflated because it includes base pairs from unaligned reads and does not correct for PCR duplicates.

### SICILIAN splicing analysis

To identify splice junctions of mouse lemur transcripts, we used SICILIAN, a statistical method used for unbiased and annotation-free detection of splice junctions^14,62^. Raw sequencing reads from the atlas (10x and SS2 datasets) were first aligned to the mouse lemur genome assembly Mmur 3.0 using the STAR algorithm with parameters chimSegmentMin = 12, chimJunctionOverhangMin = 10, chimOutType = “WithinBAM SoftClip Junctions”, and default values for the rest of the parameters. SICILIAN then extracts spliced reads that mapped to discontinuous regions of the genome. These reads could provide evidence for candidate splice junction sites but may also reflect sequencing noise or alignment error. For each of these potentially spliced scRNA-seq reads, SICILIAN estimates a read-level confidence score, which quantifies the probability that the alignment of the read is true based on features that influence sequence alignment (for example, sequence entropy of the read, sequence mismatches and number of mapping locations for the read in the genome). It then incorporates all the read-level scores for the reads aligned to each potential splice junction and computes a final confidence score (empirical *P* value) for the junction. The empirical *P* value was computed for each SS2 cell and 10x channel in the dataset, separated by lemur individual, then the median was calculated for these empirical *P* values across the dataset. Junctions with median empirical *P* < 0.1 were selected for follow-up analyses. The threshold 0.1 was determined as the optimal point in the receiver operating characteristic (ROC) curve based on simulated data in the article describing the SICILIAN method^14^ (see ROC in Fig. 1e), which identified that a threshold of 0.15 maximizes discovery sensitivity and specificity (Youden’s index) using bulk simulated RNA-seq data. Here we prioritized specificity to identify high-confidence novel junctions and therefore used a more stringent threshold (0.1).

The detected splice junctions were compared with the junctions in transcripts annotated in the NCBI annotation release 101 of the mouse lemur genome assembly Mmur 3.0. The detected splice junctions were categorized into five types (Fig. 1g). Type A refers to junctions that matched an annotated splicing pattern. Types B–D refer to junctions that aligned to an annotated gene but the specific splicing pattern is unannotated. Specifically, type B contains junctions in which both the donor (5′) and acceptor (3′) splice sites are annotated but not previously paired (for example, unannotated exon skipping), type C contains junctions in which one site is annotated and the other is not (for example, annotated donor site but unannotated acceptor site) and type D contains junctions in which both splices sites are unannotated. Type E refers to detected junctions that do not align to any annotated gene.

To examine whether the detected splice junctions are conserved in human or mouse genomes, we used the UCSC LiftOver tool from the UCSC genome browser^63^ and computed the fraction of junctions annotated in the mouse and/or human genome by considering a junction as conserved only if it had successful LiftOver conversion to the other genome (that is, both 5′ and 3′ splice sites in the lemur genome mapped successfully to unique coordinates in the other genome).

To identify cell-type-specific splicing events, we performed two-tailed MANOVA separately on the 10x data from each tissue. Cell types with fewer than ten cells or junctions present in fewer than two cells were removed from the analysis. To highlight splicing events with global effects, we analysed the genes with at least two spliced reads mapping to the gene in each cell type in the tissue. Note, however, our approach can easily be extended to include more genes expressed in only a subset of cell types. Let *C* be the set of cells, and *J* be the set of junctions for this gene. Consider cell $m\in {C}$ and junction $i\in J$ for a particular gene. Let ${n}_{m}^{(i)}$ be the number of reads mapping to junction $i$ in cell $m$. The fraction of junctional reads mapping to junction *i* in cell *m* is therefore defined as follows: ${f}_{m}^{(i)}={n}_{m}^{(i)}/\sum _{j{\epsilon }J}{n}_{m}^{(j)}$. The dataset average fraction of junction *i* was then calculated as follows: ${f}^{(i)}=\sum _{c\in C}{n}_{c}^{(i)}/\sum _{c\in C}\sum _{j\in J}{n}_{c}^{(j)}$. The scaled *z* score for junction *i* in cell *m* was defined as follows: ${z}_{m}^{(i)}=\left(\sqrt{\sum _{j\in J}{n}_{m}^{(j)}}\right)({f}_{m}^{(i)}-{f}^{(i)}\,)/\sqrt{{f}^{(i)}(1-{f}^{(i)}\,)}$. MANOVA was then performed using the cellular *z* scores of each junction for the gene as input and the cell type (free annotation) as output. This analysis generated, for each gene, a *P* value of all cell types in the tissue having the same multivariate mean junctional expression. Benjamini–Hochberg correction was then applied to all *P* values. To identify a list of candidate junctions with the most significant cell-type differential splicing, a stringent threshold (corrected *P* < 10^–16^) was used, which resulted in 545 junctions.

### SAMap analysis to study the conservation of gene expression patterns across species

To compare expression patterns of homologous genes across the human, lemur and mouse genomes, we used the SAMap method^17,64^. In addition to the lemur 10x cell atlas datasets, mouse and human 10x scRNA-seq datasets were retrieved from Tabula Muris Senis^65^ and Tabula Sapiens^66^, respectively. We applied SAMap to these datasets to compare the cell-type expression patterns of homologous genes across the three species. Although the SAMap algorithm does not require cell-type labels, having comparable annotations simplifies the interpretation of the mapping results. Therefore, we limited the analysis to the tissues (lung and skeletal muscle) that were re-annotated using the same standards as described in the accompanying paper^1^. This cross-species data with new unified cell-type designation can be found on Globus (see Data availability).

SAMap was used to simultaneously map genes and cells across the three species (human, lemur and mouse). For each pairwise combination of species, SAMap first detects homologous genes (sequence homologues) through bidirectional BLAST analysis of the transcriptomes of the two species, as annotated by Ensembl and NCBI. A cross-species gene-to-gene graph is then generated, with edges connecting a gene in one species and a homologous gene in the other species and edge weights assigned as sequence similarity of the gene pairs. The homology graphs from all pairwise comparisons of species were combined into one, tripartite adjacency matrix. Using this initial gene graph, SAMap projects the three scRNA-seq datasets into a joint, lower-dimensional manifold representation. This joint manifold enables estimation of similarity between cells and genes across species. Note that the SAMap method considers not only one-to-one orthologues but also integrates many-to-one, one-to-many and many-to-many orthologous genes as well as the non-orthogonal relationship between non-orthologous genes, which are commonly ignored in cross-species comparisons given their complexity. Next, the expression correlations between homologous genes were calculated in the initial joint manifold to re-weight the edges of the gene–gene homology graph. Using the re-weighted homology graph as the new input, SAMap then iterates until convergence to generate a final joint manifold. The expression correlation between homologous genes of the two species calculated across the joint manifold quantifies the similarity of the expression patterns of two genes. Homologous gene pairs with an expression correlation higher than 0.3 were deemed expression homologues; that is, homologues that share similar expression patterns across mapped cells. Triads of mapped expression homologues from human, lemur and mouse datasets were identified.

We then examined for each expression homologue triad whether the three gene pairs were assigned as orthologous genes in NCBI and/or Ensembl (Supplementary Table 8). We further examined for each expression homologue triad whether the lemur gene is named or unnamed in NCBI annotation release 101 of the mouse lemur genome assembly Mmur 3.0 (with only a locus identifier, for example, ‘Loc__’ or ‘orf’). Expression homologue triads were then categorized into three types (Fig. 1l). Type ‘named orthologue’ refers to triads that consist of three orthologous gene pairs, and the lemur gene is named accordingly. Type ‘unnamed orthologue’ refers to triads of three orthologous gene pairs but the lemur gene is unnamed. Type ‘non-orthologue’ refers to triads that contain at least one non-orthologous gene pair, regardless of the naming status of the lemur gene. Supplementary Table 5 lists the expression homologues detected in this study.

Quantification of genes that are named (with a gene symbol), unnamed (only a locus identifier, for example, ‘Loc__’ or ‘orf’, and a suggested gene description) or uncharacterized (unnamed and with no gene description) for Fig. 1k was obtained from the following databases: genome assembly Mmur 3.0 and NCBI annotation release 101 for mouse lemurs; assembly GRCh38.p13 and NCBI annotation release 109 for humans; and assembly GRCm38.p6 and NCBI annotation release 108 for mice.

### BCR analysis

To improve the annotation level of mouse lemur BCR loci (which contain immunoglobin genes), we first used BLAST^67^ with human BCR genes (retrieved from ImMunoGeneTics (IMGT)^68^) to search for unannotated variable and constant region genes (for example, *IGG*) in the mouse lemur genome (Mmur 3.0, NCBI annotation release 101). We then built a custom reference database from these retrieved mouse lemur immunoglobulin genes and the human IMGT sequences to extract transcripts and to assemble the immunoglobulin sequence for each of the 829 B cell and plasma cells from the SS2 data analysed using BASIC^69^. Immunoglobulin sequences obtained through BLAST searches of the transcriptomes from a subset of mouse lemur atlas B cells were added to the custom reference database to further improve alignment. Constant regions from both the heavy and light chain contigs assembled using BASIC were aligned to the reference database using BLAST, and the best hit (with at least 80 nucleotides of overlap) was used to assign the isotype (that is, *IGA*, *IGG*, *IGM* or *IGE* for the heavy chain, and *IGK* or *IGL* for the light chain) for each cell. Putative V and J gene families and the *CDR3* sequences from both the heavy and light chain contigs were identified using IgBlast^70^. In some cases, BASIC was not able to generate a contig for the heavy and/or light chain; therefore, the isotype was not assigned for these cells (52 and 10 cells for the heavy and light chains, respectively). In other cases, BASIC was unable to assemble a single continuous contig from both constant and variable region ends of either the heavy and/or light chain, and therefore, we submitted to BLAST and IgBLAST the two contigs constructed from each end (94 and 100 cells for heavy and light chains, respectively) or the only constructed contig from one end (147, 1, 28 and 2 cells with only heavy chain constant, heavy chain variable, light chain constant and light chain variable contigs, respectively). In rare cases, constant-region isotypes were not assigned for cells for which BLAST returned different hits from the variable and constant region ends (16 and 13 cells for heavy and light chains, respectively). Similarly, V gene families were not assigned for cells for which IgBLAST returned different hits (alignment quality V score > 100) from the constant and variable contigs (3 and 59 cells for heavy and light chains, respectively). These discrepancies may be caused by doublets of B cells and plasma cells (although applying the program Scrublet^71^ with default parameters identified only 3 out of the 80 cells with 2 different BLAST or IgBLAST hits as possible doublets) or more probably, reflect dual expression as more recently appreciated^72^. CDRH3 lengths were calculated as the number of amino acids between the canonical C at the 5′ end of the sequence and the 3′ sequence WGXG, where X is any amino acid. CDRL3 (including λ and κ chains) lengths were calculated as the number of amino acids between the canonical C at the 5′ end of the sequence and the 3′ sequence FGXG or WGXG, where X is any amino acid.

All immunoglobulin sequences assembled through BASIC were then used to determine the minimum number of constant and variable region alleles in the mouse lemur genome. These sequences were aligned using MAFFT^73^ and then manually corrected using Geneious Prime (v.2021.1.1; https://www.geneious.com). Because somatic mutation patterns in *IGV* genes can render a single V gene indistinguishable from separate but closely related alleles, we estimated a minimum number of V genes based on the number of V loci that occur in the current assembly of the genome. Long-read sequences covering this region would help determine the true number of V gene loci.

Clonal lineages were identified as groups (*n* ≥ 2) of cells in a single individual lemur with the same light chain isotype and identical CDRH3 and CDRL3 lengths, with both having at least 80% identity across the cells.

### MHC gene expression analysis

The methods used to examine mouse lemur MHC gene structure, to extract MHC gene expression from the atlas and to re-annotate MHC genes are detailed in a previous study^21^. In brief, raw fastq files from 10x scRNA-seq data from each organ for all individual lemurs were mapped against a MHC reference sequence extracted from the Mmur 3.0 genome assembly according to the bacterial artificial chromosome (BAC) sequences^74,75^, as well as the known expressed mouse lemur class I *Mimu-W01-04* (ref. ^76^) (GenBank accession numbers are provided in Supplementary Note 3) using bowtie2 (ref. ^77^). The mapping results were assessed for mismapping of reads, allelic variation and the possible presence of additional genes through manual inspection using the Integrative Genomics Viewer (IGV)^78^ and Geneious Prime (v.2021.2.2). The fastq files were also ‘probed in silico’ by searching for reads that contained sequences specific to the known genes. This approach confirmed the absence of expression of particular MHC genes. For the analysis of expression levels, a reference specific to each individual was created and used with bowtie2 to map the reads extracted from the raw fastq files for each tissue. The sequences used were restricted to the final 600 bp (comprising exon 5 through the 3′ UTR) to avoid complications from potential recombinant sequences. Manual inspection of the results from the blood 10x scRNA-seq files was used to determine a mapping quality (MAPQ) threshold for each gene. The sequence alignment map (SAM) file from the mapping was converted to a BAM file and then divided into individual BAM files for each gene. These individual files were then filtered to remove reads below the MAPQ threshold. For the remaining reads, the cell barcode and UMI were counted. The expression level was normalized as read counts per 10,000 UMIs and then natural log transformed. Counts for each MHC gene (raw and normalized) are available in the metadata for every h5ad file in Figshare (https://figshare.com/projects/Tabula_Microcebus/112227). SS2 data were not used owing to limited data available regarding allelic variation and recombination between alleles and/or genes that is prevalent in the MHC. The lack of phase information for the SS2 data made it impossible to accurately assign all sequences to specific genes (only the terminal 3′ 600–650 bp could be assigned with confidence to a particular class I gene). Discarding the upstream information would have biased the expression-level results. Thus, we chose to focus on the 10x dataset, for which the majority of the sequences were obtained from a single region that fell within 600–650 bp of the 3′ end and therefore could be unambiguously assigned to a specific gene.

### Chemokine ligand and receptor expression analysis

A list of human chemokine receptors was compiled from the literature^79,80^ and their cognate ligands were obtained from CellPhoneDB^81^ (Supplementary Table 7). We included the four atypical chemokine receptors, which induce G-protein-independent downstream signalling^82^, as well as the chemerin (encoded by *RARRES2*) receptors (*CMKLR1*, *GPR1* and *CCRL2*) given their established dual role in immune and adipokine chemoattraction^83^. The chemokine *CXCL17*, without a known receptor, was also included. Of the 25 identified receptors, a corresponding lemur orthologue annotated in NCBI was found for all except *CCR2*. Of the 45 cognate ligands, a corresponding lemur orthologue was identified for 32. The expression level of each of the lemur orthologues across all cell types in the 10x dataset is summarized in Supplementary Fig. 3c. Cell-type expression levels for each gene were then binarized (that is, expressed or not expressed) based on absolute and relative thresholds for the purpose of building an interaction network, per below. First, an absolute threshold was applied, which required that a gene is expressed at non-zero levels in at least 5% of cells of a cell type and with a mean expression level of at least 0.5 across all cells from that cell type. Second, a relative threshold was applied, whereby for each gene, a ceiling expression level was defined as the expression level of the 99th percentile of all cell types that passed the first threshold (to remove outliers with abnormally high expression levels). Cell types with a receptor gene mean expression level above 5% of the ceiling were deemed to be expressing the receptor. A higher threshold (20%) was applied for ligand genes given that ligands are diffusible and therefore require high levels to be functional.

To build a chemokine interaction network across all cell types in the atlas, connections (edges) were drawn between cell types (nodes) expressing a ligand and cell types expressing the cognate receptor. Self-loops were allowed, wherein a cell type expressed both the ligand and the corresponding receptor. Connections between cell types from different organs (other than blood) were excluded given the short effective intercellular communication distances of chemokine signals. Note that edges are directed such that cell type A expressing a ligand and cell type B expressing the cognate receptor formed a separate edge from cell type B expressing the same ligand and cell type A expressing the cognate receptor. Multiple connections in the same direction between two nodes (that is, two cell types with more than one receptor–ligand interaction) were counted as a single edge. The network density was calculated as the number of edges identified divided by the total number of possible edges in the network: $\frac{{N}_{{edges}}}{{N}_{{nodes}}^{2}}$. The density was calculated separately for the following networks: interactions across all cell types in the atlas; only immune cell types; only non-immune cell types; and between immune and non-immune cell types.

### Cross-organ immune cell analysis

Immune subpopulations were identified and annotated through the systematic subclustering of the lymphoid and myeloid compartment in each tissue for every individual lemur, then adjusted through inspection using cellxgene after integration of tissues across all individuals to ensure consistency of cell-type labelling, as described in the accompanying paper^1^. Clusters branching off the main group were labelled with a differentially expressed gene (DEG) (for example, neutrophil (CCL13^+^), neutrophil (IL18BP^+^) and B cell (SOX5^+^)), and cells expressing proliferative markers (MKI67 and TOP2A) were appended with ‘PF’ (for example, B cell (PF)). For macrophages, their identities as tissue-resident macrophages based on published marker genes (see supplementary table 1 in the accompanying paper^1^) was indicated by appending the corresponding name (for example, macrophage (Kupffer cell), macrophage (microglial cell)), given that a clear distinction from monocyte-derived macrophages was challenging (with the exception of lung tissue-resident alveolar and monocyte-derived interstitial macrophages, which were confidently distinguished on the basis of canonical markers and labelled as such). Identification of DEGs for each subpopulation was performed using two-tailed Wilcoxon rank-sum tests, selecting genes with log fold change ≥ 1 and *P* < 0.05 after adjustment by using Benjamini–Hochberg correction.

For the cross-organ monocyte–macrophage analysis, all granulocyte–monocyte precursors, monocytes and macrophages from the atlas were extracted for further analysis. Data were integrated across the four lemur individuals and then across the scRNA-seq methods (10x and SS2 datasets) using the FIRM algorithm^84^ to correct for batch effects. In this integrated UMAP, monocyte populations co-clustered across tissues, whereas macrophage populations were generally separated by tissue. We therefore tried additional FIRM integration across tissues; however, tissue-specific separation of macrophage types and bladder monocytes from lemur L2 remained. Therefore, we did not perform tissue-level FIRM integration for the final UMAP to avoid potential computational bias from overcorrection. We then examined the expression levels of known monocyte and macrophage markers reported in the literature as well as the distribution of monocytes and macrophages from each tissue in the FIRM-integrated UMAP (Extended Data Fig. 8 and Supplementary Fig. 4). In addition to the tissue-specific and tissue-resident populations highlighted in Supplementary Fig. 4, we found that pancreatic and heart macrophages formed separate populations. However, these results were excluded from further analysis because they probably resulted from technical issues. That is, the DEGs for pancreatic macrophages were broadly expressed in other cell types of the same tissue (signal spreading), and the heart sample had overall fewer transcripts per cell (lower quality).

The neutrophil developmental trajectory was based on embedding of neutrophils in the FIRM-integrated UMAP of the entire atlas (as described in the accompanying paper^1^). This resulted in co-clustering of neutrophils by the individual and tissue, which enabled recapitulation of the developmental trajectory. We also tried FIRM integration of neutrophils alone (by individual and scRNA-seq methods). However, this resulted in separation of neutrophils by tissue and individual, which was largely driven by batch effects (no biologically meaningful DEGs were identified across most clusters). This result suggests that neutrophils are more molecularly homogeneous across tissues compared with other cell types such as macrophages. The trajectory was obtained using an in-house algorithm that detects the density ridge of the cell distribution on the FIRM-integrated UMAP embedding, as described in the accompanying paper^1^, with the direction of the trajectory manually assigned on the basis of the expression of neutrophil maturation markers. Similar to the neutrophils, the FIRM-integrated UMAP of B cells and plasma cells showed global separation of plasma cells and B cells. However, further cell separation was driven by batch effects. In the atlas-wide UMAP, the clear separation between B cells and plasma cells precluded further trajectory analysis.

### Endometrial cancer analysis

Uterine cancer was identified by scRNA-seq and later confirmed by histopathology in both of the female lemurs (L2 and L3). Both had metastases, with L2 showing spread to the lung and L3 to an intra-abdominal lymph node. We analysed lung metastasis in lemur L2 and the primary tumour in lemur L3. The uterus of L2 was not analysed by scRNA-seq because we were unaware of the tumour at the time of tissue collection. For lemur L3, we were unable to sequence the metastasis given the liquefactive necrotic nature of the tissue.

To compare the novel lung epithelial cell cluster in L2 (retrospectively identified as endometrial tumour cells metastasized from the uterus) with all other cell types of the atlas, we examined the correlation scores (Fig. 3d) and UMAP embedding (Extended Data Fig. 9e) of their gene expression profiles using the methods described in the accompanying paper^1^. Here we extracted results of the metastatic tumour cell type. In brief, to calculate the cell-type pairwise correlation scores with the lung metastatic tumour cell type in lemur L2, atlas data were first integrated across individuals, tissues and scRNA-seq methods (10x and SS2) using FIRM^84^, and FIRM-generated principal component coefficients were calculated for each cell. The coefficients were then averaged across all cells of a cell type and used to calculate the Pearson’s correlation scores between every atlas cell type and the metastatic cells. The lung cell type in L2 that is a hybrid of metastatic and AT2 cells, which could be doublets of the two cell types (although Scrublet^71^ only identified one of these five cells as a possible doublet) was excluded from the analysis. The lung metastatic tumour cells in L2 had high correlation (0.94) with the uterine non-ciliated epithelial cells (FXYD4^+^MUC16^+^) in L3, the presumptive primary tumour. Other cell types with high correlation scores to the metastatic cells included kidney ductal and secretory cells (0.80–0.95), pancreatic ductal cells (0.85–0.88), other uterine epithelial cells (0.70–0.89), fat urothelial cells (0.87), liver cholangiocytes (0.86) and brain ependymal (0.65).

To generate the cell-type UMAP (Extended Data Fig. 9e), gene expression levels were averaged across cells for each cell type (10x dataset, excluding low-quality cell types and ones represented by <4 individual cells). Expression levels were normalized (0 to 1 scale) to the maximal value of each gene across all cell types, and the normalized cell-type gene expression matrix was projected onto a 2D space with cosine distances between pairs of cell types as input.

Differential gene expression analysis was performed on lung metastatic cells versus all other lung epithelial cells and on uterine FXYD4^+^MUC16^+^ epithelial cells versus all other uterine epithelial cells (10x datasets) using two-tailed Wilcoxon rank-sum tests (*P* < 0.05, after adjustment using the Benjamini–Hochberg method), and selected examples are presented in Fig. 3e.

### Adipocyte analysis

Adipocytes and adipo-CAR cells were extracted from the FIRM-scaled and integrated data of the entire atlas (1,231 cells, 10x and SS2 datasets, see accompanying paper^1^). The top 3,000 highly variable genes in the FIRM-transformed gene count table of adipocytes and adipo-CAR cells were selected, and dimensionality reduction by principal component analysis was performed (top 13 principal components) to generate a 2D UMAP of adipocytes and adipo-CAR cells. Differential gene expression analysis on the UCP1^high^ and UCP1^low^ adipocyte populations (L2 and L4, 10x data) was performed using two-tailed Wilcoxon rank-sum tests (*P* < 0.05, after adjustment using the Benjamini–Hochberg method), and example genes were selected for presentation in Fig. 3i. Similarly, differential gene expression analysis was performed between the adipocytes of each fat depot of L2 (BAT, GAT, MAT and SCAT), and the top ten genes enriched in each depot were selected for presentation in Extended Data Fig. 10e.

Most of the adipocytes in the atlas were isolated from fat depots, for which the tissue-dissociation protocol was designed to enrich for the stromal vascular fraction and exclude adipocytes (see the supplementary methods in the accompanying paper^1^). Most were from L2 (Extended Data Fig. 10a), whose adipocytes in fat depots surrounding several tissues (for example, kidney, spine and uterus) were generally small, predominantly multilocular, densely stained and mitochondrial rich (Extended Data Fig. 10d). These are also features of brown or beige adipocytes in humans and mice. They intermingled with small, unilocular adipocytes with a single lipid droplet, which resemble white adipocytes. By contrast, adipocytes from L3 and L4 were generally larger and most were unilocular (Extended Data Fig. 10d). These may be harder to capture using current scRNA-seq protocols, so may have contributed to the lower yield of adipocytes for L3 and L4.

### Identification of PS genes and analysis of their expression patterns in lemur and human genomes

A list of human and lemur orthologous genes with no corresponding mouse orthologue was compiled by merging human, mouse lemur and mouse homology assignments from NCBI, Ensembl and MGI databases using a similar method used to compile the list of one-to-one-to-one gene orthologues for the comparison of cell types across the three species in the accompanying paper^1^. We began by compiling all human protein-coding genes annotated in NCBI (taxonomy identifier (ID): 9606), then merged the corresponding mouse lemur and mouse orthologues from NCBI (gene_info.gz and gene_orthologs.gz from https://ftp.ncbi.nlm.nih.gov/gene/DATA/, accessed February 2020 and August 2023). We next added Ensembl gene ID numbers, gene names and lemur or mouse orthologue assignments from Ensembl Biomart (Ensembl Genes v.99, February 2020), using the Ensembl gene ID (variable ‘Gene_stable_ID’) for each NCBI gene ID (variable ‘NCBI_gene_ID’) in Ensembl Biomart. MGI mouse gene ID numbers, gene names and orthologue assignments (none provided for lemur) from the Jackson Laboratory (HOM_MouseHumanSequence.rpt from http://www.informatics.jax.org/downloads/reports/, Feb 2020) were added using the MGI homology ID (variable ‘HomoloGene_ID’) attributed to each NCBI gene ID (variable ‘EntrezGene_ID’) in the MGI database. The Online Mendelian Inheritance of Man (OMIM)^55^ genetic disorder phenotype associated with each human gene (genemap2.txt from https://www.omim.org/downloads January 2022, variable ‘Phenotypes’) was added using the gene name (variable ‘Approved_Gene_Symbol’) in the OMIM database.

A human gene was identified as sharing an orthologue with lemurs if at least one such assignment was made by either Ensembl or NCBI, and/or as sharing an orthologue with mouse if at least one such assignment was made by NCBI, Ensembl or MGI (Supplementary Table 8). This approach resulted in 539 human genes with an assigned lemur, but no mouse, orthologue (that is, PS genes), which corresponded to 388 unique lemur Ensembl gene IDs and to 425 unique lemur NCBI genes IDs (not all orthologues are annotated in both NCBI and Ensembl). Note that gene orthology assignments from NCBI, Ensembl and MGI are periodically updated; thus, these numbers may change in the future. Transcripts were detected for 401 out of the 425 PS NCBI-annotated genes, and their expression patterns across all lemur atlas cell types (10x dataset) were visualized in heatmaps and dot plots and qualitatively categorized by whether their expression was enriched (higher or restricted expression) or depleted in one or more tissues or organs or compartments (Supplementary Table 9 and Supplementary Fig. 5). Expressed genes that did not show any of these expression patterns were categorized as ‘not enriched in any category’.

Gene set enrichment analysis of the 539 PS genes was performed using gprofiler2 in R^85^, searching for overrepresented gene sets (relative to all human-annotated genes) in gene ontology terms, biological pathways, regulatory DNA elements, human disease gene annotations and protein–protein interaction networks, using default parameters (for example, user_threshold = 0.05, correction_method = “g_SCS” for Fisher’s one-tailed test with multiple testing correction).

We further analysed evolutionary conservation in the expression patterns of the PS genes that have one-to-one orthology mapping between humans and lemurs (Supplementary Table 9). The analysis followed a similar pipeline as described in the accompanying paper^1^, in which we compared across human, lemur, mouse and macaque using one-to-one orthologues (that is, not including any PS genes). We analysed cells from the lung, skeletal muscle, liver (epithelial cell only), testis (germ cell only), as well as bone marrow and spleen (immune cells only). To unify cell-type annotation, data of different species were integrated using Portal with around 15,000 one-to-one orthologues, and cells were re-annotated for consistent designation across all species. Here we applied the same cross-species cell type annotation and compared between human and lemur only, with lemur data from the Tabula Microcebus atlas^1^ and human data from the Human Lung Cell Atlas^79^ (lung), ref. ^86^ (testis) and Tabula Sapiens^66^ (rest of the tissues).

With manual curations, we identified a total of 398 PS genes with one-to-one orthology mapping between humans and lemurs. Note that NCBI and Ensembl occasionally have inconsistent orthology assignments. For example, one database may assign a one-to-one mapping, whereas the other database may assign a one-to-many mapping. In such cases, we prioritized NCBI mapping but also maximized coverage by retaining the orthologues with identical gene symbols or description in both species. Next, we analysed 346 of the PS genes that were reported in all scRNA-seq datasets described above. Because the number of annotated genes were different between humans and lemurs, we normalized the transcript counts of the PS genes against the background of all one-to-one orthologues and then log transformed the expression levels (that is, ln(UP10K + 1)). For each PS gene, mean expression (*E*_max_) in the maximally expressed cell type in each species was quantified. Next, we filtered for PS genes with notable expression across the analysed cell types, requiring *E*_max_ > 0.5 in each species, or *E*_max_ > 0.1 in each species and *E*_max_ > 1.5 in at least one species. This resulted in a total of 93 PS genes for which we quantified their expression pattern similarity between humans and lemurs. Mean cell type expression levels across the orthologous cell types were normalized by *E*_max_ of the same species, and Pearson’s correlation coefficients between humans and lemurs were calculated and reported in Supplementary Table 9. Most (55%, 51 out of 93) of the analysed PS genes had a correlation coefficient above 0.5, which indicated conservation in their expression patterns between lemurs and humans.

### Analysis of natural mutations

We performed whole-genome sequencing for each of the four lemurs (L1–L4) used to create the atlas, along with 31 additional lemurs originating from the same laboratory colony (median 54× coverage). Methods for the whole-genome sequencing and analysis pipeline are detailed in a recent study^58^ and in the accompanying paper^1^. In brief, genomic DNA libraries were generated through Tn5-based tagmentation, and indexed and PCR-amplified for 150 bp paired-end short-read Illumina sequencing. Sequencing reads were aligned to the current Mmur 3.0 genome assembly (NCBI RefSeq assembly accession GCF_000165445.2) and germline variants were identified using the Sentieon DNAseq workflow. Variants were annotated and filtered, and functional impact predictions were made using the SnpEff & SnpSift toolbox. This resulted in around 45 million total variants across all 35 lemurs. To identify functionally significant rare nonsense variants relevant for this study, we first filtered for three criteria: (1) allele frequency < 0.5; (2) base call quality > 99.9%; and (3) homozygous or heterozygous variants present in at least one of the lemurs (L1–L4) used for the atlas. This resulted in 6,905 variants. Next, we refined this list by filtering for variants for which their respective genotypes were identified in all sequenced animals, focusing on nonsense variants by looking at those predicted to cause frameshift mutations, alterations in the stop codon and variants computationally predicted to cause NMD. This narrowed our final list to 713 unique variants found in 713 genes (1 per gene).

To analyse the transcriptional impact of these nonsense variants, we compared cell-type-specific gene expression of the affected gene in the four lemurs used to create the atlas. We prioritized genes that were abundantly expressed and potentially functionally important (for example, absent in mice). Cell-type specific scRNA-seq reads were identified by their 10x barcodes, parsed from the original post-alignment BAM files for each lemur and counted using Samtools (v.1.16.1) across the respective gene. This enabled discernment of the number of reads with the reference allele versus those with the alternative allele at the variant locus, along with the total number of reads mapping to the gene. Quantifying and analysing the differing allelic expression patterns of these genes, in the presence and absence of the variant, enabled us to verify nonsense variants linked to significant reductions in gene expression. To compare gene expression in WT and mutant individuals, cells were grouped by their cell-type designation (without distinguishing their tissue of origin). Cell-type average expression was then calculated for each individual separately (excluding cell types with <35 profiled cells (10x)). Cell types with no expression or low expression of the gene (ln(UP10K + 1) < 0.3) in control animals were not plotted in Fig. 5e,i,m and Extended Data Fig. 11c.

### Reporting summary

Further information on research design is available in the Nature Portfolio Reporting Summary linked to this article.

## Online content

Any methods, additional references, Nature Portfolio reporting summaries, source data, extended data, supplementary information, acknowledgements, peer review information; details of author contributions and competing interests; and statements of data and code availability are available at 10.1038/s41586-025-09114-8.

## Supplementary information

Supplementary Information Supplementary Notes, Supplementary Figures, legends for the Supplementary Tables and Supplementary References.

Reporting Summary

Supplementary Tables Supplementary Tables 1–10.

Supplementary Data 1 Source data for Supplementary Fig. 1.

Supplementary Data 2 Source data for Supplementary Fig. 2.

Supplementary Data 3 Source data for Supplementary Fig. 3.

Supplementary Data 4 Source data for Supplementary Fig. 4.

Supplementary Data 5 Source data for Supplementary Fig. 5.

Supplementary Data 6 Source data for Supplementary Fig. 6.

## Source data

Source Data Fig. 1

Source Data Fig. 2

Source Data Fig. 3

Source Data Fig. 4

Source Data Fig. 5

Source Data Extended Data Fig. 1

Source Data Extended Data Fig. 2

Source Data Extended Data Fig. 4

Source Data Extended Data Fig. 5

Source Data Extended Data Fig. 6

Source Data Extended Data Fig. 8

Source Data Extended Data Fig. 9

Source Data Extended Data Fig. 10

Source Data Extended Data Fig. 11

## Data Availability

Tabula Microcebus mouse lemur scRNA-seq gene expression counts and UMI tables, and cellular metadata used in this study are available from Figshare (https://figshare.com/projects/Tabula_Microcebus/112227)^87^, and can be explored interactively using the UCSC Cell Browser on the Tabula Microcebus portal (https://tabula-microcebus.ds.czbiohub.org/). A histological atlas of all the tissues analysed is also available on the portal. Raw sequencing data (fastq files) are available from Globus (https://app.globus.org/file-manager?origin_id=c9fc0a15-54a0-4182−8d64-fd8afc12f1fc&origin_path=%2F). For sequence alignment, *M.* *murinus* genome assembly (Mmur 3.0, NCBI accession GCF_000165445.2) and gene annotation file (NCBI Refseq annotation release 101) were obtained from NCBI FTP sites (https://www.ncbi.nlm.nih.gov/datasets/genome/GCF_000165445.2/; https://ftp.ncbi.nlm.nih.gov/genomes/all/annotation_releases/30608/101/). To classify DE uTARs as protein-coding or non-protein-coding, the reference database of mammalian proteins from UniProt was used (https://www.ebi.ac.uk/reference_proteomes/). Human BCR genes were retrieved from IMGT (https://www.ebi.ac.uk/ipd/imgt/hla/), and lemur MHC genes were retrieved from GenBank (accession numbers in Supplementary Note 3). A list of cognate ligands to human chemokine receptors was manually downloaded from CellPhoneDB (https://www.cellphonedb.org/index.html, March 2024). For cross-species analysis, human 10x data were from Tabula Sapiens^66^ for the liver, spleen and bone marrow (https://figshare.com/projects/Tabula_Sapiens/100973) and the Human Lung Cell Atlas^79^ for the lung (https://www.synapse.org/#!Synapse:syn21041850/wiki/600865). Human testis drop-seq data were from a previous study^86^ (https://www.ncbi.nlm.nih.gov/geo/query/acc.cgi?acc=GSE142585). Mouse data were all from 10x data of Tabula Muris Senis^65^ (https://figshare.com/articles/dataset/Processed_files_to_use_with_scanpy_/8273102/2), except for the testis, which was based on a previously published 10x dataset^88^ (https://www.ebi.ac.uk/biostudies/arrayexpress/studies/E-MTAB-6946). For orthologous gene compilation and to quantify named, unnamed and uncharacterized genes, data were obtained from NCBI (gene_info.gz and gene_orthologs.gz from https://ftp.ncbi.nlm.nih.gov/gene/DATA/), Ensembl Biomart (Ensembl Genes v.99) and MGI (HOM_MouseHumanSequence.rpt from http://www.informatics.jax.org/downloads/reports/). The list of human genes with associated genetic disorders was obtained from OMIM (genemap2.txt from https://www.omim.org/downloads). Source data are provided with this paper.

## Code availability

Custom computer codes are available on Globus (https://app.globus.org/file-manager?origin_id=c9fc0a15-54a0-4182-8d64-fd8afc12f1fc&origin_path=%2F). Additional software and packages used are described below. Raw sequencing data were processed using Cell Ranger (v.2.2, 10x Genomics) for 10x data and with STAR aligner (v.2.6.1a), skewer (v.0.2.2), RSEM (v.1.3.1) and HTSEQ (v.2.0) for SS2 data. Downstream analyses were performed using R (v.4.3.0), Python (v.3.6 and v.3.9) and Matlab (v.2020b). Seurat (R package, v.2.3.0), Scanpy (v.1.8) and cellxgene (v.1.0.1) were used for cell clustering and annotation. Cell gradients were generated using Slingshot (v.2.14.0) and a custom program developed in Matlab (trajectory analysis: https://github.com/Shixuan1/scRNAseq_trajectory_analysis) using Matlab built-in functions (for example, pca), the Image Processing Toolbox (Matlab v.2020b) and a Matlab umap package (https://www.mathworks.com/matlabcentral/fileexchange/71902). scRNA-seq data integration used custom programs developed by co-authors, including FIRM (https://github.com/mingjingsi/FIRM) and Portal (https://github.com/YangLabHKUST/Portal). TAR analysis used an author-generated program (http://github.com/fw262/TAR-scRNA-seq), groHMM tool (v.1.40.3), BLASTn (https://blast.ncbi.nlm.nih.gov/Blast.cgi), an author-generated program Nf core/predictorthologs (https://github.com/czbiohub-sf/nf-predictorthologs), DIAMOND blast (https://github.com/bbuchfink/diamond) and Infernal cmscan (https://www.ebi.ac.uk/Tools/rna/infernal_cmscan/). SICLIAN analysis used an author-generated program (https://github.com/salzmanlab/SICILIAN) and the UCSC LiftOver tool (https://genome.ucsc.edu/cgi-bin/hgLiftOver). SAMap analysis used an author-generated program (https://github.com/atarashansky/SAMap, v.1.0.15). BCR analysis used BLASTn (https://blast.ncbi.nlm.nih.gov/Blast.cgi), IgBLAST (https://www.ncbi.nlm.nih.gov/igblast/), BASIC (v.1.5.0), MAFFT (v.7) and Geneious Prime (v.2021.1.1). MHC analysis used bowtie2 (v.2.3.5), IGV (v.2.8.0) and Geneious Prime (v.2021.2.2). Gene set enrichment analysis used gprofiler2 in R (v.0.2.1). Natural mutant analysis used Sentieon (v.202308.03). For data visualization, dot plots, sina plots, violin plots, line plots, bar plots, box plots, heatmaps, pie charts, interaction plots, error bars and contour figures were generated using the following Python, R and Matlab packages: Python: pandas (v.1.1.5), numpy (v.1.19.3), anndata (v.0.7.4), scanpy (v.1.6.0), matplotlib (v.3.3.2), igraph (v.0.7.1), seaborn (v.0.9.0) and ‘louvain’ (v.0.6.1); R packages: ggplot2 (v.3.4.4), gplots (v.3.1.3), readr (v.2.1.4), dplyr (v.1.1.2), reshape2 (v.1.4.4), patchwork (v.1.1.3), RColorBrewer (v.1.1.3), ggrepel (v.0.9.4), aplot (v.0.1.10), ggdendro (v.0.1.23), Matrix (v.1.6.4), here (v.1.0.1), pheatmap (v.1.0.12), tidyr (v.1.3.0), cowplot (v.1.1.1) and circlize’ (v.0.4.15); and Matlab built-in functions: plot, scatter, violinplot, imagesc, contour, bar, box, errorbar and pie.
