# Supplementary material for: Mouse lemur cell atlas informs primate genes, physiology and disease
Source: Nature. 2025 Jul 30;644(8075):185–96. doi: 10.1038/s41586-025-09114-8 (PMC12328237; doi:10.1038/s41586-025-09114-8)
Supplement: Supplementary file 1 — Supplementary Notes, Supplementary Figures, legends for the Supplementary Tables and Supplementary References. [file 41586_2025_9114_MOESM1_ESM.pdf]

nature portfolio

https://doi.org/10.1038/s41586-025-09114-8

Supplementary information

Mouse lemur cell atlas informs primate genes, physiology and disease

In the format provided by the authors and unedited

Mouse lemur cell atlas informs primate genes, physiology, and disease

Camille Ezran[1, 2 ‡], Shixuan Liu[1, 2, 3 ‡], Stephen Chang[1, 2, 4‡], Jingsi Ming[5], Lisbeth A. Guethlein[6, 7], Michael F.Z. Wang[8, 9], Roozbeh Dehghannasiri[1, 10], Julia Olivieri[1, 11], Hannah K. Frank[12, 13], Alexander Tarashansky[14, 15], Winston Koh[16, 17], Qiuyu Jing[18], Olga Botvinnik[15], Jane Antony[19], The Tabula Microcebus Consortium*, Angela Oliveira Pisco[15], Jim Karkanias[15], Can Yang[20], James E. Ferrell, Jr.[1, 3], Scott D. Boyd[12], Peter Parham[6, 7], Jonathan Z. Long[12, 21], Bo Wang[14], Julia Salzman[1, 10], Iwijn De Vlaminck[8], Angela Ruohao Wu[18, 22, 23], Stephen R. Quake[14, 15, 24]†, Mark A. Krasnow[1, 2] †

‡ Co-first authors
† Corresponding authors: krasnow@stanford.edu (M.A.K.), steve@quake-lab.org (S.R.Q.)

SUPPLEMENTARY INFORMATION

Table of contents

Supplementary Notes (pages 3-12)
1. Tabula Microcebus Consortium author contributions
2. Summary of mouse lemur clinical conditions
3. MHC annotation
4. Analysis of chemokine receptors and ligands
5. Expansion of Extended Data Figure 7 legend
6. Analysis of activated neutrophils
7. Analysis of monocytes and macrophages

Supplementary Figures (pages 13-34)
1. Gene sequence conservation between human and lemur versus human and rodents or higher monkeys
2. Additional examples of alternatively spliced isoforms across atlas cell types
3. Cell-cell interactions maps based on chemokine signaling
4. FIRM-integrated UMAP of atlas monocytes and macrophages
5. Expression patterns of PS genes across lemur cell types
6. Comparison of expression patterns of PS genes across human and lemur cell types

Supplementary Table Legends (pages 35-38)
1. Differentially-expressed-uTARs and their homologous genes and cellular expression levels
2. uTAR expression counts across 10x dataset
3. Splice junctions and categories identified by the SICILIAN analysis
4. Genes differentially spliced across cell types
5. Expression homologue triads of human, lemur, and mouse detected by SAMap
6. Expression of immunoglobulin isotypes in atlas B cells and plasma cells
7. Chemokine receptor and cognate ligand genes
8. Human genes and their lemur and mouse orthology assignments
9. PS genes and their expression patterns in the mouse lemur and human
10. Gene sets enriched in PS genes

Supplementary References (pages 39-41)

Supplementary Notes

Supplementary Note 1. The Tabula Microcebus Consortium authorship contributions

Lead authors

Camille Ezran[1, 2 ‡], Shixuan Liu[1, 2, 3 ‡], Stephen Chang[1, 2, 4‡], Jingsi Ming[5], Lisbeth A. Guethlein[6, 7], Michael F.Z. Wang[8, 9], Roozbeh Dehghannasiri[1, 10], Julia Olivieri[1, 11], Hannah K. Frank[12, 13], Alexander Tarashansky[14, 15], Winston Koh[16, 17], Qiuyu Jing[18], Olga Botvinnik[15], Jane Antony[19], Angela Oliveira Pisco[15], Jim Karkanias[15], Can Yang[20], James E. Ferrell, Jr.[1, 3], Scott D. Boyd[12], Peter Parham[6, 7], Jonathan Z. Long[12, 21], Bo Wang[14], Julia Salzman[1, 10], Iwijn De Vlaminck[8], Angela Ruohao Wu[18, 22, 23], Stephen R. Quake[14, 15, 24]†, Mark A. Krasnow[1, 2] †

‡ Co-first authors

† Corresponding authors: krasnow@stanford.edu (M.A.K.), steve@quake-lab.org (S.R.Q.)

Leadership and coordination

Trainees: Camille Ezran[1, 2], Shixuan Liu[1, 2, 3]

Principal investigators: Mark A. Krasnow[1, 2], Stephen R. Quake[14, 15, 24], Angela Ruohao Wu[18, 22, 23], Iwijn De Vlaminck[8], Bo Wang[14], Julia Salzman[1, 10], Jim Karkanias[15], Angela Oliveira Pisco[15]

Donor recruitment and animal husbandry

Megan A. Albertelli[25], Caitlin J. Karanewsky[1, 2], Jozeph L. Pendleton[1, 2], Fabienne Aujard[26], Martine Perret[26], Liza Shapiro[27], Andriamahery Razafindrakoto[28], Hajanirina Noëline Ravelonjanahary[28], Patricia Wright[29], Anne D. Yoder[30], Cathy V. Williams[31], Robert Schopler[31], Ute Radespiel[32], Jean-Michel Verdier[33], Corinne Lautier[33], E. Christopher Kirk[27], Rebecca Lewis[27]

Necropsy, sample collection, biobanking, histology, and pathology

Kerriann M. Casey[25], Megan A. Albertelli[25], Caitlin J. Karanewsky[1, 2], Jozeph L. Pendleton[1, 2], Camille Ezran[1, 2], Shixuan Liu[1, 2, 3], Kyle J. Travaglini[1, 2], Astrid Gillich[1, 2], Zicheng Zhao[2, 25], Stephen Chang[1, 2, 4], Elias Godoy[25], Jérémy Terrien[26], Jacques Epelbaum[26, 34], Dita Gratzinger[12], Katherine Lucot[12], Thomas Montine[12]

Tissue processing, annotation, and analysis [Principal investigators]

Aorta: Jessica D'Addabbo[4, 35], Isaac Bakerman[4], [Patricia Nguyen[4, 35, 36]]

Bladder: Aaron Kershner[1, 19], Karim Mrouj[19], [Philip Beachy[3, 19, 37, 38]]

Blood: Rahul Sinha[19], Camille Ezran[1, 2], Yue Zhang[2, 39], Shixuan Liu[1, 2, 3], Caitlin J. Karanewsky[1, 2], [Irving L. Weissman[19], Mark A. Krasnow[1, 2]]

Bone (limb, spine): Thomas H. Ambrosi[19], Malachia Hoover[19], Alina Alam[19], [Charles Chan[19]]

Bone marrow: Rahul Sinha[19], SoRi Jang[1, 2], Camille Ezran[1, 2], [Irving L. Weissman[19], Mark A. Krasnow[1, 2]]

Brain (cortex, brainstem, cerebellum): Avin Veerakumar[1, 2, 14], Peng Li[1, 2], Andrea R. Yung[1, 2], Connor V. Duffy[2, 40], Song-Lin Ding[41], [Ed S. Lein[41], Silvana Konermann[1, 2], Liqun Luo[2, 39], Trygve E. Bakken[41], Justus M. Kebschull[42], Rebecca D. Hodge[41], Mark A. Krasnow[1, 2]]

Colon/Small intestine: Taichi Isobe[43], [Michael F. Clarke[19]]

Diaphragm/Limb muscle: Antoine de Morree[44, 45], Biter Bilen[44], Jean Farup[44, 45], Andoni Urtasun[44], Jengmin Kang[44], [Thomas A. Rando[44]]

Eye: Ming Chen[40], BaoXiang Li[46], Varun Ramanan Subramaniam[46], Shravani Mukherjee[46], Aditi Swarup[46], Lily Kim[40], Bronwyn Scott[46], Ahmad Al-Moujahed[46], [Albert Y. Wu[46], Douglas Vollrath[40], Lubert Stryer[47]]

Fat: Nicholas Schaum[44], Jingsi Ming[5], Shixuan Liu[1, 2, 3], Amanda L. Wiggenhorn[12, 48], [Tony Wyss-Coray[44, 49], Jonathan Z. Long[12, 21], Angela Ruohao Wu[18, 22, 23]]

Ganglion (vagus nerve): Yin Liu[1, 2], [Mark A. Krasnow[1, 2]]

Heart: Stephen Chang[1, 2, 4], [Mark A. Krasnow[1, 2]]

Hypothalamus & Pituitary: Andrea R. Yung[1, 2], Shixuan Liu[1, 2, 3], [Mark A. Krasnow[1, 2]]

Kidney: Shixuan Liu[1, 2, 3], Ahmad Nabhan[1, 2], Lolita Penland[15], Yue Zhang[2, 39], Andrea R. Yung[1, 2], [Gabriel Loeb[51], Mark A. Krasnow[1, 2]]

Liver: Shengda Lin[51], Shixuan Liu[1, 2, 3], Honor Paine[52], Deviana Burhan[52], Aris Taychameekiatchai[52], [Steven Artandi[1, 36, 53], Bruce Wang[52]]

Lung: Kyle J. Travaglini[1, 2], Ahmad Nabhan[1, 2], F. Hernán Espinoza[1, 2], Astrid Gillich[1, 2], [Christin Kuo[54], Ross Metzger[4, 54], Mark A. Krasnow[1, 2]]

Lymph node: SoRi Jang[1, 2], Lolita Penland[15], [Norma Neff[15], Mark A. Krasnow[1, 2]]

Mammary gland: Zhen Qi[19], [Michael F. Clarke[19]]

Microbiome: Rebecca Culver[40], [Kerwyn C. Huang[7, 14, 15]]

Pancreas: Patrick Neuhöfer[1, 36, 53], Charles A. Chang[37, 55], Yan Hang[37, 55], [Seung K. Kim[36, 37, 55, 56], Steven Artandi[1, 36, 53]]

Prostate: Hannah N. W. Weinstein[57, 58, 59], Paul Allegakoen[57], [Franklin W. Huang[57]]

Salivary gland: Sivakamasundari V[19], [Philip Beachy[3, 19, 37, 38]]

Skin: Song Eun Lee[44, 49], [Tony Wyss-Coray[44, 49]]

Spleen: Lolita Penland[15], Hannah K. Frank[12, 13], Dita Gratzinger[12], [Norma Neff[15], Scott D. Boyd[12]]

Testes: Kazuteru Hasegawa[1, 36, 53], Hosu Sin[37], Jingsi Ming[5], [Steven Artandi[1, 36, 53], Margaret T. Fuller[37, 40], Angela Ruohao Wu[18, 22, 23]]

Thymus: Isaac Bakerman[4], Jessica D'Addabbo[4, 35], [Patricia Nguyen[4, 35, 36]]

Tongue: Wan-Jin Lu[19], Ankit Baghel[39], [Philip Beachy[3, 19, 37, 38]]

Trachea/Thyroid/Parathyroid: William Kong[19], [Philip Beachy[3, 19, 37, 38]]

Uterus/Abdominal mass: Lolita Penland[15], [Norma Neff[15]]

Library processing, sorting, and sequencing

Stephen Chang[1, 2, 4], Lolita Penland[15], Carly Israel[15], Rene Sit[15], Jennifer Okamoto[15], Ashley Maynard[15], Michelle Tan[15], Yue Zhang[2, 39], Rahul Sinha[19], Shixuan Liu[1, 2, 3], Camille Ezran[1, 2], Kyle J. Travaglini[1, 2], Antoine de Morree[44, 45], Ahmad Nabhan[1, 2], Youcef Ouadah[1], Jalal Baruni[1, 2, 39, 60], Timothy Ting-Hsuan Wu[1, 2], Robert C. Jones[14], Norma Neff[15], Maurizio Morri[15], Spyros Darmanis[15], Sheela Crasta[15], Jia Yan[15], Aditi Agrawal[15], Shelly Huynh[15], Brian Yu[15], James Webber[15]

Global data analysis [Principal investigators]

General analysis: Shixuan Liu[1, 2, 3], Camille Ezran[1, 2], [Mark A. Krasnow[1, 2]]

Sequence alignment: Stephen Chang[1, 2, 4], Kyle J. Travaglini[1, 2], Rahul Sinha[19], Rene Sit[15], [Mark A. Krasnow[1, 2], Irving L. Weissman[19], Stephen R. Quake[14, 15, 24]]

Integration algorithms: Jingsi Ming[5], Jia Zhao[20], Gefei Wang[20], [Angela Ruohao Wu[18, 22, 23], Can Yang[20]]

Molecular trajectory: Shixuan Liu[1, 2, 3], Camille Ezran[1, 2], Timothy Ting-Hsuan Wu[1, 2], Weilun Tan[7], [Mark A. Krasnow[1, 2]]

Global cell type comparison: Shixuan Liu[1, 2, 3], [Mark A. Krasnow[1, 2]]

Cross-species comparison: Shixuan Liu[1, 2, 3], Camille Ezran[1, 2], Jia Zhao[20], Gefei Wang[20], Alexander Tarashansky[14, 15], Olga Botvinnik[15], Saba Nafees[15], Zhengda Li[3], Kyle J. Travaglini[1, 2], Antoine de Morree[44, 45], [Mark A. Krasnow[1, 2], Angela Ruohao Wu[18, 22, 23], Can Yang[20], James E. Ferrell, Jr.[1, 3], Jim Karkanias[15], Bo Wang[14], Stephen R. Quake[14, 15, 24], Thomas A. Rando[44]]

Spermatogenesis (accompanying paper): Jingsi Ming[5], Hosu Sin[37], Kazuteru Hasegawa[1, 36, 53], Camille Ezran[1, 2], Shixuan Liu[1, 2, 3], [Angela Ruohao Wu[18, 22, 23], Margaret T. Fuller[37, 40], Steven Artandi[1, 36, 53], Can Yang[20], Mark A. Krasnow[1, 2]]

uTAR: Michael F.Z. Wang[8, 9], [Iwijn De Vlaminck[8]]

SICILIAN: Roozbeh Dehghannasiri[1, 10], Julia Olivieri[1, 11], [Julia Salzman[1, 10]]

SAMap: Alexander Tarashansky[14, 15], [Bo Wang[14]]

Major histocompatibility locus: Lisbeth A. Guethlein[6, 7], Camille Ezran[1, 2], [Peter Parham[6, 7], Mark A. Krasnow[1, 2]]

B cell receptor locus: Hannah K. Frank[12, 13], Camille Ezran[1, 2], [Scott D. Boyd[12], Mark A. Krasnow[1, 2]]

Cross-tissue neutrophil trajectory: Camille Ezran[1, 2], Shixuan Liu[1, 2, 3], [Mark A. Krasnow[1, 2]]

Cross-tissue monocyte and macrophage comparison: Qiuyu Jing[18], Camille Ezran[1, 2], Shixuan Liu[1, 2, 3], [Angela Ruohao Wu[18, 22, 23], Mark A. Krasnow[1, 2]]

Chemokine expression: Camille Ezran[1, 2], [Mark A. Krasnow[1, 2]]

Metastatic tumor: Jane Antony[19], Camille Ezran[1, 2], [Michael F. Clarke[19], Mark A. Krasnow[1, 2]]

Endothelium: Camille Ezran[1, 2], Astrid Gillich[1, 2], Geoff Stanley[14], [Ross Metzger[4, 54], Mark A. Krasnow[1, 2], Stephen R. Quake[14, 15, 24]]

Adipocytes: Jingsi Ming[5], Jinxurong Yang[18], Camille Ezran[1, 2], Shixuan Liu[1, 2, 3], [Jonathan Z. Long[12, 21], Angela Ruohao Wu[18, 22, 23], Mark A. Krasnow[1, 2], Can Yang[20]]

Primate-selective/mouse-absent genes: Winston Koh[16, 17], Camille Ezran[1, 2], Shixuan Liu[1, 2, 3], [Mark A. Krasnow[1, 2]]

Global hormone signaling: Shixuan Liu[1, 2, 3], [James E. Ferrell, Jr.[1, 3], Mark A. Krasnow[1, 2]]

OnClass: [Sheng Wang[61]]

Data portal/Online histology atlas: Zicheng Zhao[2, 25], Snigdha Agarwal[15], Kyle Awayan[15], Erin McGeever[15], Venkata N. P. Vemuri[15], [Stephen R. Quake[14, 15, 24], Angela Oliveira Pisco[15]]

Natural mutations: Shixuan Liu[1, 2, 3], Stephen Chang[1, 2, 4], Pranav V. Lalgudi[1, 40], [Mark A. Krasnow[1, 2]]

Principal investigators (A-Z)

Megan A. Albertelli[25], Steven Artandi[1, 36, 53], Fabienne Aujard[26], Trygve E. Bakken[41], Philip Beachy[3, 19, 37, 38], Scott D. Boyd[12], Kerriann M. Casey[25], Charles Chan[19], Michael F. Clarke[19], Jacques Epelbaum[26, 34], James E. Ferrell, Jr.[1, 3], Margaret T. Fuller[37, 40], Dita Gratzinger[12],

Franklin W. Huang[57], Kerwyn C. Huang[7, 14, 15], Jim Karkanias[15], Justus M. Kebschull[42], Seung K. Kim[36, 37, 55, 56], Silvana Konermann[1, 2], Mark A. Krasnow[1, 2], Christin Kuo[54], Ed S. Lein[41], Shengda Lin[51], Gabriel Loeb[51], Jonathan Z. Long[12, 21], Liqun Luo[2, 39], Ross Metzger[4, 54], Jingsi Ming[5], Thomas Montine[12], Norma Neff[15], Patricia Nguyen[4, 35, 36], Peter Parham[6, 7], Martine Perret[26], Angela Oliveira Pisco[15], Stephen R. Quake[14, 15, 24], Thomas A. Rando[44], Ute Radespiel[32], Julia Salzman[1, 10], Liza Shapiro[27], Lubert Stryer[47], Jérémy Terrien[26], Jean-Michel Verdier[33], Iwijn De Vlaminck[8], Douglas Vollrath[40], Bo Wang[14], Bruce Wang[52], Sheng Wang[61], Irving L. Weissman[19], Patricia Wright[29], Tony Wyss-Coray[44, 49], Albert Y. Wu[46], Angela Ruohao Wu[18, 22, 23], Can Yang[20], Anne D. Yoder[30]

Writing

Camille Ezran[1, 2], Shixuan Liu[1, 2, 3], Jingsi Ming[5], Lisbeth A. Guethlein[6, 7], Michael F.Z. Wang[8, 9], Roozbeh Dehghannasiri[1, 10], Julia Olivieri[1, 11], Hannah K. Frank[12, 13], Alexander Tarashansky[14, 15], Qiuyu Jing[18], Jane Antony[19], Peter Parham[6, 7], Jonathan Z. Long[12, 21], Bo Wang[14], Iwijn De Vlaminck[8], Angela Ruohao Wu[18, 22, 23], Stephen R. Quake[14, 15, 24], Mark A. Krasnow[1, 2]

Supplementary Note 2. Summary of mouse lemur clinical conditions

Details of the clinical conditions for the lemurs, including histopathological diagnosis, complete blood count (CBC) and complete metabolic panel (CMP) at time of euthanasia, necropsy report, and medical history are described in Casey et al.[1] and accompanying paper[2]. Histology of all tissues in each individual can be viewed at the Tabula Microcebus web portal. Below we summarize clinical conditions relevant to this study:

L1 (male): widespread fibrous osteodystrophy with pathologic fractures; kidneys with bilateral acute tubular necrosis and pre-existing chronic renal disease; suppurative rhinitis, pharyngitis and tracheitis, as well as pulmonary emphysema.

L2 (female): mild fibrous osteodystrophy without pathologic fracture; kidneys with bilateral chronic renal disease (hydronephrosis, interstitial nephritis, and amyloidosis); uterus with diffuse uterine adenocarcinoma with suppurative metritis with tissue swab culture positive for *Klebsiella* species, *Enterococcus faecalis*, and aerobic gram-positive rods; lung with metastatic uterine adenocarcinoma with necrosis and hemorrhage, as well as multifocal bronchopneumonia with tissue swab culture positive for *Klebsiella* species; sinusitis and rhinitis; bladder with suppurative cystitis.

L3 (female): mild fibrous osteodystrophy without pathologic fracture; kidneys with bilateral mild diffuse glomerulopathy and interstitial nephritis/fibrosis; uterus with diffuse uterine adenocarcinoma with necrotic cysts; a 2-cm abdominal mass (suspected mesenteric lymph node) and a peri-adrenal lymph node with metastatic uterine adenocarcinoma.

L4 (male): mild fibrous osteodystrophy without pathologic fracture; kidneys with bilateral moderate diffuse glomerulopathy, interstitial nephritis/fibrosis and renal pelvis hemorrhage; severe pulmonary hemorrhage.

Supplementary Note 3. MHC annotation

We used our organism-wide atlas to enhance gene annotation of the major histocompatibility complex (MHC). MHC class II genes encode structurally-related alpha (A genes) and beta (B genes) subunits of transmembrane heterodimers expressed on dendritic and other professional antigen-presenting cells. They are loaded with exogenous peptides derived from phagocytosed pathogens and proteins, which serve as ligands for TCRs on helper T cells to initiate an adaptive immune response[3]. Our allele-specific analysis of expression of MHC class II genes across the full atlas shows that each individual mouse lemur expresses one or two alleles of each MHC class II gene except *DOA* and *DOB*, which were not expressed in any of them, supporting their designation as pseudogenes based on sequence analysis[4] (Extended Data Fig. 4a-c). The other class II genes all appear to be single, non-duplicated genes, including two (*DQA*, *DQB*) that are currently annotated as duplicated and one (*DRB*) as triplicated, the latter from failed assembly of two genomic fragments of the second *DRB* allele[5] (Extended Data Fig. 4e). *DQA*, *DQB*, *DRA*, and *DRB* are expressed at higher levels and in more cells than the other class II genes (*DMA*, *DMB*, *DPA*, *DPB*)[5] (Extended Data Fig. 4h). Number of alleles for *DPA* and *DPB* could not be estimated given few cells express the gene across all lemurs analyzed.

MHC class I genes encode the variable alpha chain that together with beta-2 microglobulin form widely-expressed transmembrane heterodimers that are loaded with peptides

derived from cytosolic proteins, which serve as ligands for TCRs on cytotoxic T cells, or for KIRs (killer cell immunoglobulin-like receptors) or KLRs (killer cell lectin-like receptors) on NK cells, to destroy cells expressing viral, tumor or other "non-self" proteins[3,6]. No expression was detected in our atlas of genes in the canonical MHC locus at chromosome 6 (Extended Data Fig. 4a), supporting their assignment as pseudogenes from sequence analysis[7]. All of the expressed mouse lemur class I genes mapped to a separate, duplicated cluster on chromosome 20q (Extended Data Fig. 4b), an organization unique to mouse lemur[5] (Extended Data Fig. 4d). Our allele-specific expression analysis identified 11 expressed genes in the cluster, four (*Mimu-168*, *-W03*, *-W04*, *-249*) we designate as "classical" based on their high and widespread expression, and the rest we designate “non-classical” (*Mimu-180ps*, *-191*, *-202*, *-208*, *-218*, *-229ps*, *-239ps*) including three previously thought to be pseudogenes (*Mimu-180ps*, *-229ps*, *-239ps*) based on sequence analysis (Extended Data Fig. 4c, f-h).

Given the polymorphic nature of the MHC complexes[8], it remains challenging to distinguish some genes from allelic variation. Three of the classical genes (*Mimu-168*, *-W03*, *-W04*) are very similar in sequence and appear to be divergent allelic lineages, with some individuals (L1, L3) missing (or not expressing) one or more of the three genes. Three of the non-classical genes (*Mimu -208*, *-218*, *-229ps*) are also missing transcripts in two or three individuals (Extended Data Fig. 4c). The current genome assembly (Mmur 3.0[9]) contains gaps separating the complex into three sequence islands, rearrangement of which better matches the available BAC sequence[7] (Extended Data Fig. 4b), as detailed in Guethlein et al.[5]; long range sequencing is needed to reveal the full number and order of genes for each individual.

GenBank accession numbers of lemur MHC gene sequences used to create a MHC reference sequence against which 10x atlas scRNA-seq reads were mapped.

- Class I BAC[4,7] sequences: FP236833.
- Class I W01-04[10] sequences: AJ297588, AJ297589, AJ297590, AJ302085.
- Mmur 3.0 reference genome annotated by NCBI (Refseq Annotation Release 101): Class I (chr. 20): XM_01273726, XM_012763577, XM_020282714, XR_002222271, XM_020282717, XM_020282716, XM_012763578, XM_012737265; Class II (chr. 6): XM_012774641, XM_012765726,XR_002223954, XM_020286955, XM_020286957, XM_020286956, XM_012761793, XM_020286954, XM_012791383, XM_012791391, XM_012791371, XM_012791379, XM_020286938, XM_012791375; Pseudogenes (chr. 6): NC_033665, XM_020287333.
- Other reference cDNA sequences used (all microcebus species): DRA: HQ222945, HQ222944, HQ222943; DRB: LN610539, LN610542, HE801960, HE801959, LN610553, HE801958, HE801963, LN610548, AJ555836, LN610550, AJ555839, HE801954, AJ555835, LN610543, LN610554, LN610544, LN610551, LN610547, AJ431269, AJ431270, LN610549, LN610541, AJ431266, HE801962, HE801956, HE801964, HE801955, HE801961, AJ431267, AJ431268, HE801957, AB078195, AB078196, AB078295, AB078297, AB078299, AB078290, AB078291, AB078203, AB078276, AB078222, AB078256, AJ555838, LN610545, LN610552, LN610546, AJ555837, AJ830741, AB078284, LN610540, AJ555840, AJ555841, AJ830740; DQA: HQ222964, HQ222957, HQ222959, HQ222958, HQ222956, HQ222961, HQ222962, HQ222960, HQ222955, HQ222963, HQ222938, HQ222936, HQ222937; DQB:

HQ222950, HQ222951, HQ222952, HQ222954, HQ222947, HQ222946, HQ222949, HQ222948, HQ222953, HQ222939.

Supplementary Note 4. Analysis of chemokine receptors and ligands

We examined the expression of chemokine ligands and receptors across the atlas (Fig. 2a, Extended Data Fig. 5, Supplementary Fig. 3). While the ligands were broadly expressed across non-germ compartments, the receptors were mostly restricted to the immune populations (Extended Data Fig. 5a). Macrophages expressed the highest number of chemokine ligands and receptors, supporting their critical role in directing immune cell trafficking (Extended Data Fig. 5b). Activated neutrophils of the uterus (*IL18BP+*) also expressed a large number of chemokine receptors, suggesting their role in responding to inflammation. Linking chemokine-expressing cell types to the cell types expressing the corresponding receptor revealed a global cell-cell interaction network (Supplementary Fig. 3a) with a moderate density (i.e., 6.8% of all possible interactions were present). Though as expected, the network was much more densely connected between immune cells (density = 33.5%) compared to between immune and non-immune cells (3.3%) and between non-immune cells (1.4%). There were also dramatic differences in the cell specificity of different chemokine signaling pairs, with some resulting in widespread connections across a tissue and others connecting only a few specific cell types (see the example of lung and blood in Supplementary Fig. 3b).

Chemokines and their receptors showed a highly cell type specific expression pattern across the atlas (Fig. 2a, Extended Data Fig. 5c, Supplementary Fig. 3c). For example, each major type of dendritic cells (DC) in the atlas expresses a unique combination of chemokine ligands and receptors: conventional DCs (*XCR1+*), plasmacytoid DCs (*CXCL16-*), mature DC (*CCL19+*, *CCL22+*, *CCR7+*), and *IGSF6+* DCs (*CXCR3-*).

The analysis also suggested patterns of cell-cell chemoattraction as highlighted in main text (Fig. 2a, Extended Data Fig. 5c). For example, epithelial cell types of the skin, GI tract, bladder and fat urothelium express *CCL20* so can recruit receptor *CCR6*-expressing immune cells, as reported in skin of psoriasis patients[11,12]. Moreover, we identified lemur B cells, *CD4+* T cells, and mature DCs selectively expressing the receptor *CCR7*, and thus could be directed by the corresponding ligand expressing lymphatic endothelial cells (*CCL21*) and reticular cells (*CCL21*, *CCL19*) to lymph nodes[13,14], as illustrated in Extended Data Fig. 7. This may be further aided by *ACKR4*, a decoy receptor for CCL19/CCL21, expressed in other lymphatic endothelial cells, and thus could enhance local gradients of CCL19 and CCL21 in and around lymph nodes[15]. *ACKR4* was also expressed in skin keratinocytes, mesothelial cells, and lung alveolar macrophages, likely to scavenge soluble CCL19/21 and create a gradient in the tissues to guide local immune cell trafficking, as previously suggested in mice[16,17]. Reticular cells also expressed *CXCL13* and *CXCL12*, which could help attract mature B cells that express the CXCL13 receptor (*CXCR5*) and other immune cells that express the CXCL12 receptor (*CXCR4*)[18,19]. The expression patterns of *RARRES2* and its receptor (*CMKLR1*) suggest they play a similar role for monocytes and macrophages in lymph node trafficking[20].

In addition to the stereotypical expression of chemokines and/or receptors across lemur individuals and tissues under normal physiology, we also uncovered likely examples of disease-associated expression of the chemokine pathways, consistent with the clinical data and histopathology in specific individuals and tissues[1]. These elucidate the lemur inflammatory

response (as exemplified in Extended Data Fig.7). For example, the three "interferon gamma-induced chemokines" (*CXCL9*, *CXCL10*, *CXCL11*) are co-expressed at high levels in several diseased or inflamed tissues: alveolar and interstitial macrophages and capillary cells of L2's and L4's lung (Fig. 2a) (beset with uterine metastases and pulmonary hemorrhage, respectively), as well as subpopulations of capillary and mesothelial cells in L2's fat (Fig. 2a) – presumably recruiting or retaining the *CXCR3*-expressing major dendritic cell types (conventional, plasmacytoid, mature) and other leukocytes into the inflamed tissues (and possibly suppressing angiogenesis)[21,22]. Similarly, a subset of activated neutrophils, monocytes, and macrophages (see below) express higher levels of the potent neutrophil chemoattractant *CXCL8* with its receptors *CXCR1* and *CXCR2* broadly expressed across all neutrophils, allowing recruitment of additional neutrophils and amplifying inflammation (Fig. 2a).

Supplementary Note 5. Expansion of Extended Data Figure 7 legend

Schematic summary of multi-organ inflammatory processes exemplified in L2, who was diagnosed with endometrial cancer (1) with metastatic spread to lung (2) and secondary bacterial infection in both organs, as well as suppurative cystitis (3) and suspected inflammation in perigonadal fat (4). Involved cell types in bone marrow (bottom left), circulating in blood vessels (middle), in the inflamed tissues (top), and in lymph nodes (bottom right) are shown along with their marker genes, and the signals (*ligand — receptor* in tan boxes, colored as in Fig. 2a) proposed to control the local and systemic inflammatory steps (5-9) are indicated.

We detected a systematic increase in circulating immature/maturing neutrophils (as in Fig. 2c). These maturing/immature cells and other immune progenitors (e.g., granulocyte-monocyte progenitors (GMP)) are normally retained in the bone marrow by surrounding niche cells (e.g., osteo-CAR cells) through CXCL12-CXCR4 signaling[23–25] (5). Upon infection, maturing neutrophils are released from the bone marrow, through down regulation of *CXCR4* and upregulation of *CSF3R*, *CXCR1/2*[23,26,27] (6).

At the site of infection, two subtypes of activated neutrophils (designated as *IL18BP*+ and *CCL13*+) were found expressing high levels of chemoattractants that can recruit additional immune cells (7) (see below and Fig. 2a, Extended Data Fig. 6g). These activated neutrophils also express senescence markers (e.g., *CXCR4*) and decreased level of the "don't eat me signal" (*CD47)*, facilitating their trafficking to lymph node, marrow, and spleen for antigen presentation and/or clearance[28,29], guided by subtypes of lymphatic endothelial and reticular stromal cells expressing *RARRES2*, *CCL21*, and/or *ACKR4* (8).

In bladder and perigonadal fat, inflamed monocytes and macrophages express cytokines *CSF3*, *CXCL8*, and *IL1A* (7, see below). In perigonadal fat, inflamed capillary and mesothelial cells express interferon-γ induced *CXCL9/10/11*, which attract *CXCR3*-expressing immune cells[21,22] (9) and non-myelinating Schwann cells expressed elevated levels of MHC class II genes (see Extended Data Fig. 4h), suggesting an inflammation state[30]. Schematic created with BioRender under license: https://BioRender.com/r8i9ddj.

Supplementary Note 6. Analysis of activated neutrophils

We examined gene expression in the activated neutrophils present at the end of the neutrophil developmental trajectory in lemur L1-3 (Fig. 2c). They showed downregulation of mature neutrophil markers (e.g., *SELL/CD62L*, *MMP9*, *CXCR1*, *ITGAL*) that facilitate extravasation. They also showed induction of chemokines that promote homing to inflammatory sites and recruitment of additional neutrophils: *CXCL8* that recruits *CXCR1/2*-expressing maturing neutrophils, *CCL5* (*RANTES*) that recruits *CCR5*-expressing activated neutrophils and other immune cells, as well as *CCL8 (LOC105885739),* and *CCL4 (LOC105881712)*)[31–35]. Some of these genes are markers of neutrophil aging and lymph node trafficking (increased *CXCR4*, decreased *SELL/CD62L* and *CD47* ("don't eat me" signal)), indicating antigen-presentation and clearance following activation[28,29] (Extended Data Fig. 6g, 7).

Subclustering of these activated neutrophils revealed two subpopulations (*CCL13+* and *IL18BP+*), with differing tissue distributions across lemur L1-3 (Extended Data Fig. 6f-g), suggesting local factors can drive distinct activation pathways. One group, designated *CCL13+*, expresses chemoattractants for monocytes and eosinophils (*CCL15, CCL13 (LOC105859268*), *CCL2-like* (*LOC105859340/LOC105885684*))[36–38]. This population was found in L2's lung (site of metastatic adenocarcinoma and pneumonia) and sparsely in the kidney (possibly affected by neighboring bladder infection/cystitis and/or underlying chronic kidney disease) as well as in L1's lung (site of emphysema and suppurative rhinotracheitis).

The other neutrophil group, designated *IL18BP+*, shows enriched expression of genes including *IL18BP*, *ANXA5*, *SQSTM1*, *CD9*, *PTGR1*, and *CCR5*, many not previously implicated in neutrophil biology (Extended Data Fig. 6g). This population was found in L2's bladder (site of suppurative cystitis) and lung (site of metastatic adenocarcinoma and pneumonia), and sparsely in the kidney (site of chronic kidney disease) and perigonadal fat (possibly affected by neighboring uterine cancer and infection), L1's lung (site of emphysema and suppurative rhinotracheitis), and L3's uterus (site of adenocarcinoma).

Supplementary Note 7. Analysis of monocytes and macrophages

Monocytes and macrophages are phagocytic leukocytes that work with neutrophils to engulf and destroy microbial pathogens and abnormal cells such as tumor cells. Like neutrophils, monocytes formed a continuous and homogeneous trajectory emanating from hematopoietic precursor and granulocyte-monocyte progenitor (Extended Data Fig. 8a), with the exception of likely activated monocytes as described below. Interestingly, we did not otherwise detect biologically meaningful monocyte subclusters in any tissue or identify subpopulations using canonical markers that distinguish human or mouse monocyte subtypes[2] (Supplementary Fig. 4a).

In contrast, lemur macrophages were highly diverse, showing many distinct, tissue-specific subpopulations (Extended Data Fig. 8a, Supplementary Fig. 4b). Several of the tissue-specific subpopulations clustered near monocytes, suggesting they may arise from circulating monocytes and undergo tissue-specific maturation. Other subpopulations segregated away from the monocyte trajectory, suggesting these are likely stable populations of tissue-resident macrophages. Indeed, many expressed genes known to mark tissue-resident macrophages in human and mouse (Extended Data Fig. 8b, and see Table 1 in accompanying paper[2]), such as

CNS microglia (e.g., *CX3CR1*)[39,40], liver Kupffer cells (*ID3*)[41], and lung alveolar macrophages (*PPARG*)[42–44]. Bone macrophages specifically expressed osteoclast markers (*CTSK, ATP6V0D2* and *NFATC1*)[45], but they did not form a separate cluster. Pancreas macrophages clustered separately due to batch effect. However, lemur macrophages cannot be distinguish into canonical M1 ("classically activated") vs M2 ("alternatively activated") macrophages based on commonly used markers[46,47] (Supplementary Fig. 4a), though this distinction in other systems has blurred[48].

Moreover, we identified previously uncharacterized macrophage subtypes. For example, we identified a population of liver and small intestine *MS4A7+* macrophages that co-clustered and were separated from other *MS4A7-* populations in these tissues, including the likely canonical intestinal resident macrophages (*CD163+* (*LOC105869074*), *TIMD4+*) and the majority of liver Kupffer populations that express canonical mouse macrophage marker *ADGRE1+* (*F4/80*) (Extended Data Fig. 8a-c, Supplementary Fig. 4b). We also identified a cluster of *MRC1+* macrophages present in multiple tissues (fat, bladder, tongue, kidney, limb muscle) whose transcriptomic profile did not resemble that of any known resident macrophages (Extended Data Fig. 8a-c, Supplementary Fig. 4b). These results exemplify additional instances of our expanding understanding of molecular diversity within macrophages[49–51].

A small fraction of monocytes and macrophages may be activated in response to inflammation, including *CXCL10+* alveolar and interstitial macrophages of L2 and L4's lungs (see above chemokine section). We also identified a population of likely inflammatory monocytes in L2's bladder (site of suppurative cystitis) and perigonadal fat (possibly affected by nearby uterine adenocarcinoma and infection) that clustered near macrophages from the same tissues and separately from all other monocytes (Extended Data Fig. 8a). These monocytes expressed inflammation-associated genes, including *CD274/PD-L1*, *IL23A*, *AREG*, *CSF3*, *IL1A*, possibly teaming up with the activated neutrophils in these tissues to battle the local infections (cystitis and cancer) (Extended Data Fig. 8d).

Supplementary Figures

Supplementary Figure 1. Gene sequence conservation between human and lemur versus human and rodents or higher monkeys.
Scatter plot comparing pairwise sequence identity of orthologous genes of human and lemur vs. human and mouse (**a**), human and rats (**b**), human and rhesus macaque (**c**), human and crab-eating macaque (**d**), and human and marmoset (**e**), extracted from Ensembl homology database. Red line, 1-1 relationship. Bottom right of each panel, percent (numerator/denominator) of orthologues with higher sequence identity between human and lemur than between human and the other species.

Supplementary Figure 2. Additional examples of alternatively spliced isoforms across atlas cell types.
Stacked bar graphs showing percent of indicated splice isoforms expressed across atlas cell types for example genes that are differentially spliced across compartments (*CAST* (**a**), also *MYL6* in Extended Data Fig. 2) or across germ cells (*MLF1* (**b**), *TPD52L2* (**c**), *FAM92A* (**d**), and *CHPT1* (**e**)), formatted as in Fig. 1j. Cell types shown are those with spliced transcripts of the gene in ≥300 reads across ≥10 cells (except for sperm cells with fewer reads/cells). Cell types are labeled by their tissue source and designation number[2], and colored by compartment. Top of each panel, transcript structure shown with splice isoforms labeled by corresponding NCBI Refseq ID and with exons affected by alternative splicing in red. Note for *CHPT1* (**e**), the two transcripts in dashed box are combined in the analysis given insufficient read coverage to distinguish them.

Supplementary Figure 3. Cell-cell interactions maps based on chemokine signaling.
a. Global cell-cell interaction map across atlas cell types (L1-L4, 10x) based on all expressed chemokine ligand-receptor pairs analyzed (gray arcs) with the pair CXCL12→CXCR4 highlighted (orange arcs). Cell types are arranged clockwise by designation number with colored bars indicating the tissue compartment (as in panel c). Ligand CXCL12-expressing cell types indicated with orange label and receptor CXCR4-expressing cell types with adjacent black bars.
b. Lung-specific cell-cell interaction maps based on chemokine signaling with lung and circulating blood cell types included (L1-L4, 10x). Each panel separately plots specific chemokine ligand-receptor pairs (indicated on the lower left) with arcs connecting ligand-expressing cells to the receptor-expressing cells (black bars). *, presumed activated immune cell types (see Immune Section and Supplementary Notes).
c. Dot plot of mean expression of all chemokine receptors and cognate ligands analyzed (Supplementary Table 7) across all atlas molecular cell types (10x, L1-L4). Cell types are ordered by compartment, then cell type designation number[2], then tissue. [], description of genes identified by NCBI as loci: *[CCL4], LOC105881712; [CCL8], LOC105885739; [CCL11], LOC105885804; [CCL13], LOC105859268; [CXCL4], LOC105860501.*

Supplementary Figure 4. FIRM-integrated UMAP of atlas monocytes and macrophages.
a. UMAPs of atlas monocyte, and macrophages, and their progenitors, integrated by FIRM as in Extended Data Fig. 8a, with heatmap showing lemur expression of classical and non-classical monocyte markers as well as M1 and M2 macrophage markers for human and mouse. Human classical monocyte (*CD14++ CD16-*) markers: *CD14*, *CCR1*, *CCR2*, *CXCR1*, *ITGAM/CD11B*,

SELL/CD62L, *CD163*; non-classical monocyte (*CD14+ CD16++*) markers: *CD16/FCGR3A*, *CD11C/ITGAX*, *CD64/FCGR1A*, *CX3CR1*; intermediate monocytes express intermediate levels of these markers[47,52]. Mouse pro-inflammatory monocyte (*Ly6c*^{hi}) markers: *Ly6C*, *Ly6G/Gr-1*, *Ccr2*, *Sell/Cd62l*; patrolling monocyte (*Ly6c*^{lo}) markers: *Cx3cr1* and *Spn/Cd43*. In both human and mouse, M1 macrophage markers: *IL12A/B*, *IL23A*, *IL1B*, *IL6*, *TNF*; M2 macrophage markers: *IL10* and *TGFB1*[46,47]. Note lemur monocytes and macrophages either showed no clear pattern of these markers (plotted) or the respective genes remain unannotated in the lemur genome (i.e., *CCR2*, *CD64/FCGR1A*, *CD11C/ITGAX, Ly6c1*, *Ly6c2*, *Ly6G/Gr-1*, *CD43/SPN*). *[CD14L1], LOC105862649; [CD14L2], LOC105862489; [CD163], LOC105869074; [CD16L], LOC105873562.*
b. UMAPs as in Extended Data Fig. 8a separately highlighting cells from each atlas tissue, with cells (dots) colored by tissue (left), lemur individual (center), and molecular cell type designation (right).

Supplementary Figure 5. Expression patterns of PS genes across lemur cell types.
a-f. Dot plots showing mean expression of compartment-enriched (or depleted) PS genes across all atlas cell types (10x, L1-L4). Genes are organized by enriched compartments: epithelial (**a**), endothelial (**b**), stromal (**c**), immune (**d**), neural (**e**), germ (**f**). Genes are shown in more than one panel if enriched in multiple compartments. See Supplementary Table 9 for a full list of the PS genes and their description.

Supplementary Figure 6. Comparison of expression patterns of PS genes across human and lemur cell types.
Extension of Fig. 4f with dot plot showing cross-species expression of all PS genes in human and lemur lung (Lu), skeletal muscle (M), liver, testes, bone marrow and spleen (B/S). Rows are orthologous genes, indicated with their human gene symbol (and respective lemur gene symbol in parenthesis if different). Columns are cell types, displayed as doublets of dots showing the respective expression in human and lemur.

Supplementary Fig. 1

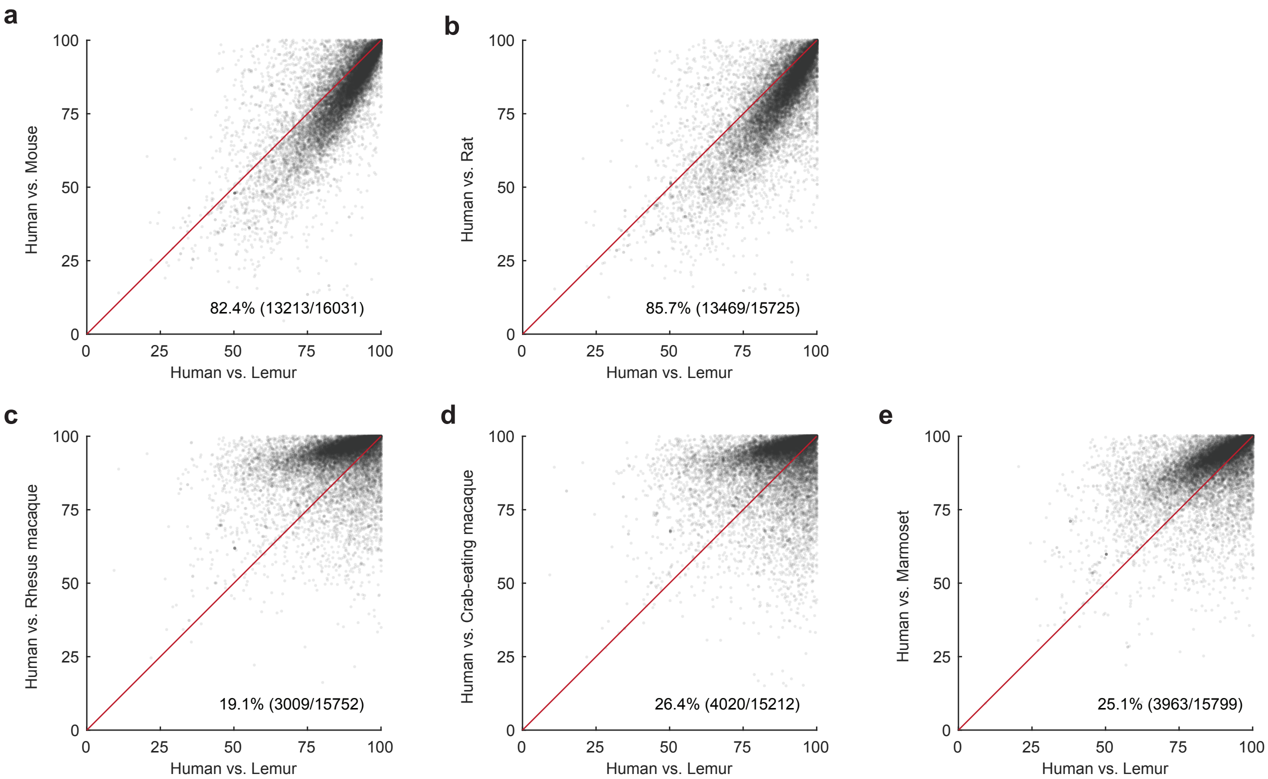

Supplementary Fig. 2

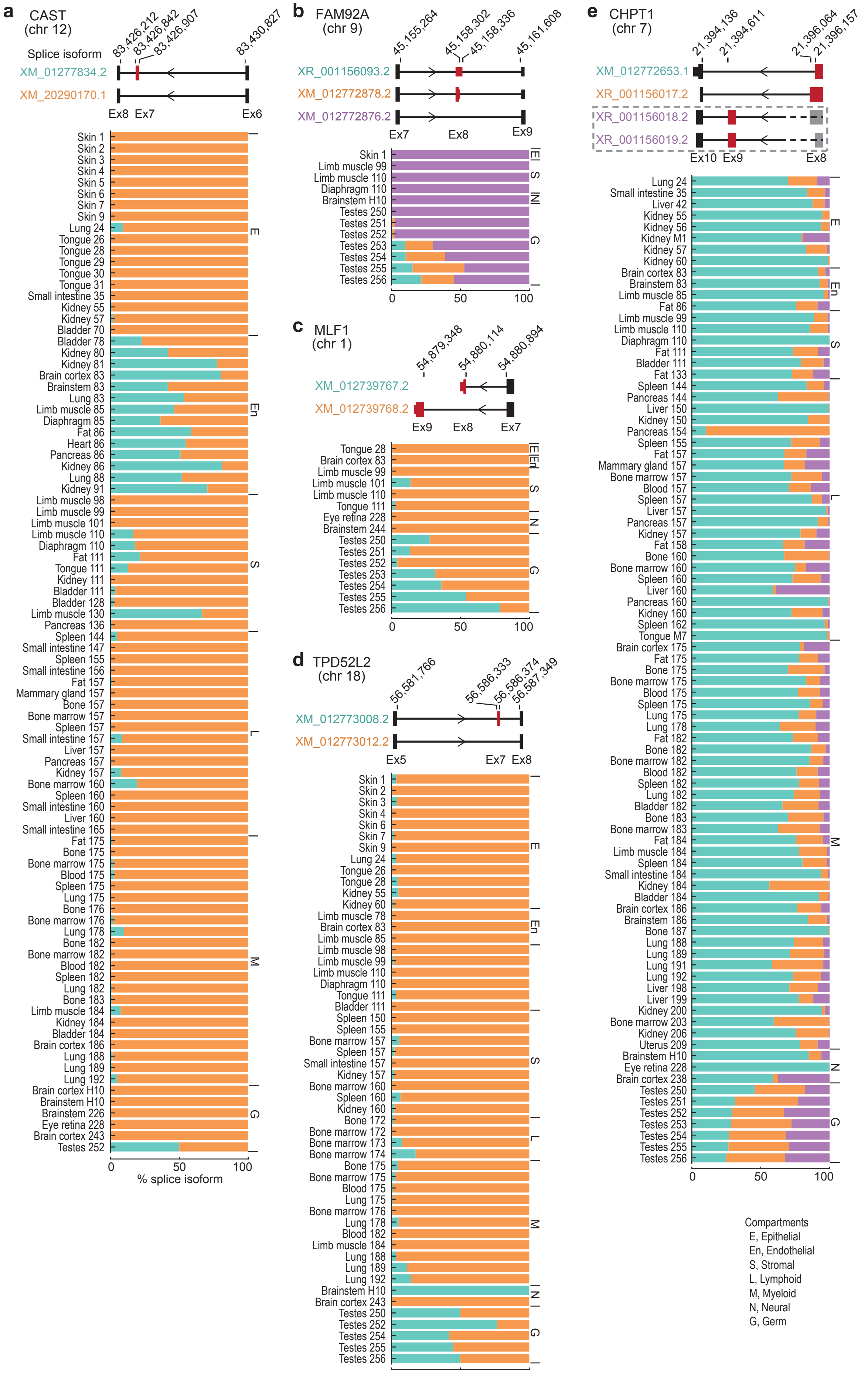

Supplementary Fig. 3

a

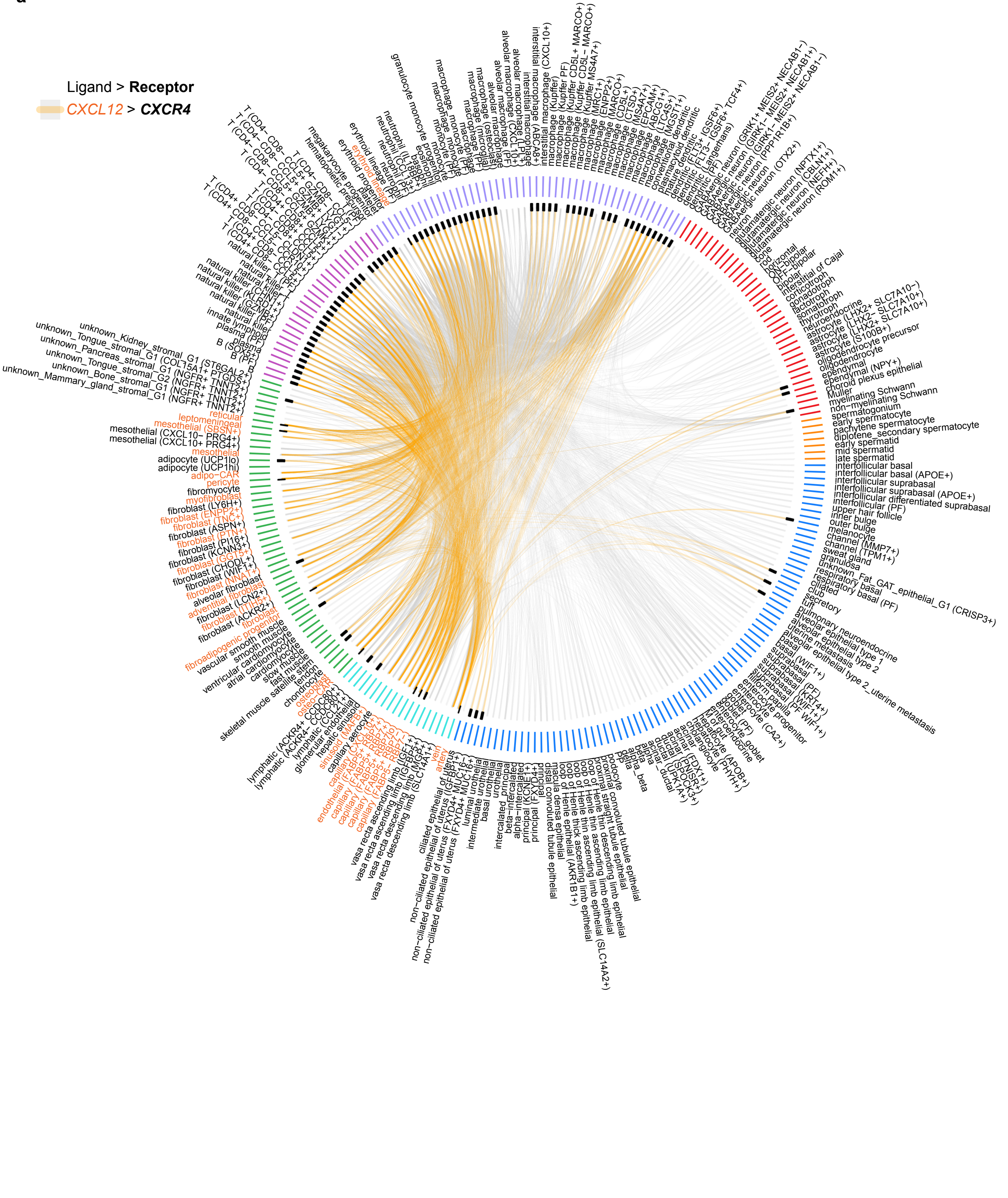

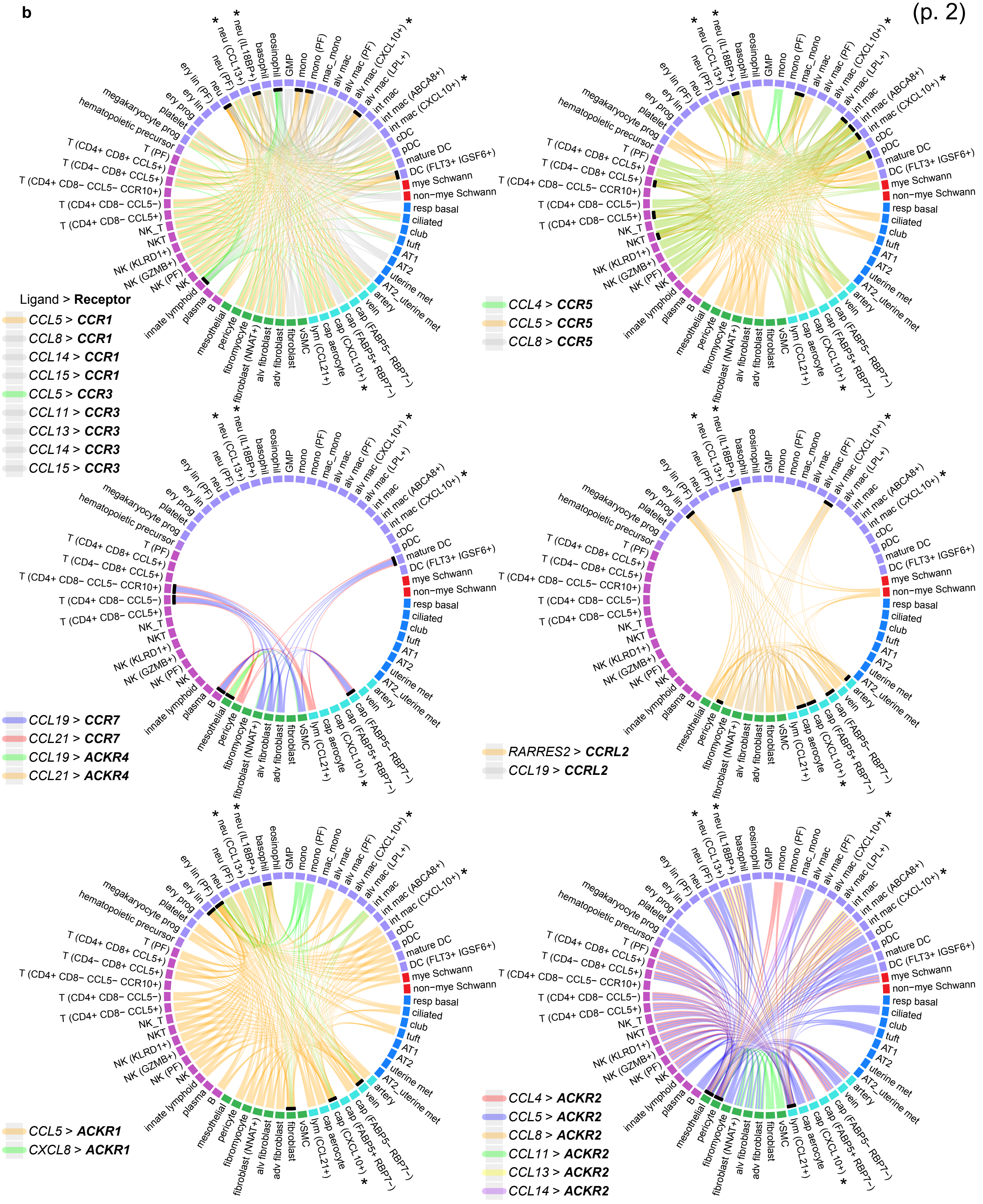
b
neu (CCL13+) *
neu (IL18BP+) *
basophil
eosinophil
GMP
mono
mono (PF)
mac_mono
alv mac
alv mac (PF)
alv mac (CXCL10+) *
alv mac (LPL+)
int mac
int mac (ABCA8+)
int mac (CXCL10+) *
cDC
pDC
mature DC
DC (FLT3+ IGSF6+)
mye Schwann
non−mye Schwann
resp basal
ciliated
club
tuft
AT1
AT2
uterine met
AT2_uterine met
artery
vein
cap (FABP5− RBP7−)
cap (FABP5+ RBP7−)
cap (CXCL10+) *
cap aerocyte
lym (CCL21+)
vSMC
fibroblast
adv fibroblast
alv fibroblast
fibroblast (NNAT+)
fibromyocyte
pericyte
mesothelial
B
plasma
innate lymphoid
NK
NK (PF)
NK (GZMB+)
NK (KLRD1+)
NKT
NK_T
T (CD4+ CD8− CCL5+)
T (CD4+ CD8− CCL5−)
T (CD4+ CD8− CCL5− CCR10+)
T (CD4− CD8+ CCL5+)
T (CD4+ CD8+ CCL5+)
T (PF)
hematopoietic precursor
megakaryocyte prog
platelet
ery prog
ery lin
ery lin (PF)
neu
neu (PF)
Ligand > Receptor
CCL5 > CCR1
CCL8 > CCR1
CCL14 > CCR1
CCL15 > CCR1
CCL5 > CCR3
CCL11 > CCR3
CCL13 > CCR3
CCL14 > CCR3
CCL15 > CCR3
CCL4 > CCR5
CCL5 > CCR5
CCL8 > CCR5
CCL19 > CCR7
CCL21 > CCR7
CCL19 > ACKR4
CCL21 > ACKR4
RARRES2 > CCRL2
CCL19 > CCRL2
CCL5 > ACKR1
CXCL8 > ACKR1
CCL4 > ACKR2
CCL5 > ACKR2
CCL8 > ACKR2
CCL11 > ACKR2
CCL13 > ACKR2
CCL14 > ACKR2

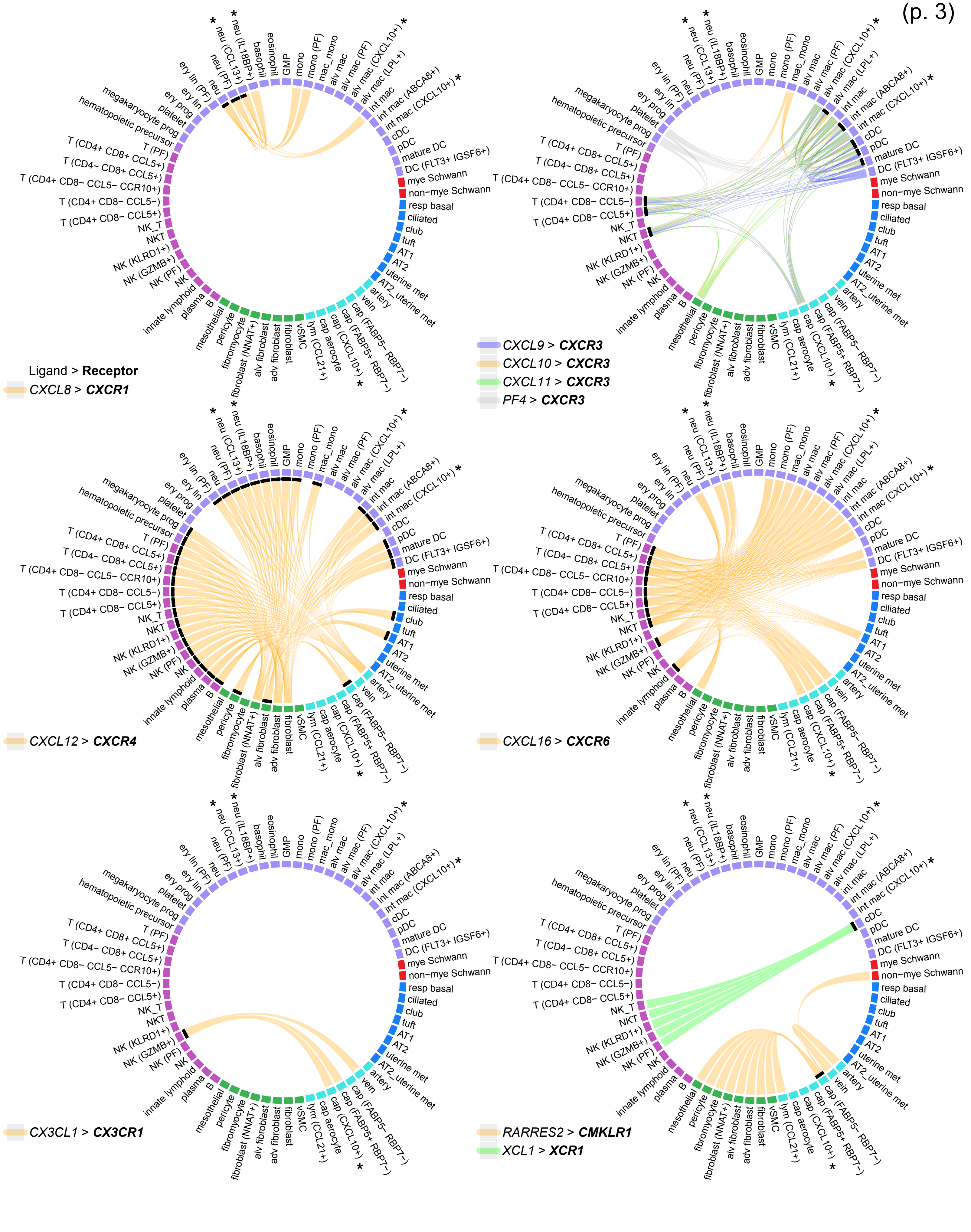
Ligand > Receptor
CXCL8 > CXCR1
CXCL9 > CXCR3
CXCL10 > CXCR3
CXCL11 > CXCR3
PF4 > CXCR3
CXCL12 > CXCR4
CXCL16 > CXCR6
CX3CL1 > CX3CR1
RARRES2 > CMKLR1
XCL1 > XCR1
GMP
mono
mono (PF)
mac_mono
alv mac
alv mac (PF)
alv mac (CXCL10+) *
alv mac (LPL+)
int mac
int mac (ABCA8+)
int mac (CXCL10+) *
cDC
pDC
mature DC
DC (FLT3+ IGSF6+)
mye Schwann
non−mye Schwann
resp basal
ciliated
club
tuft
AT1
AT2
uterine met
AT2_uterine met
artery
vein
cap (FABP5− RBP7−)
cap (FABP5+ RBP7−)
cap (CXCL10+) *
cap aerocyte
lym (CCL21+)
vSMC
fibroblast
adv fibroblast
alv fibroblast
fibroblast (NNAT+)
fibromyocyte
pericyte
mesothelial
B
plasma
innate lymphoid
NK
NK (PF)
NK (GZMB+)
NK (KLRD1+)
NKT
NK_T
T (CD4+ CD8− CCL5+)
T (CD4+ CD8− CCL5−)
T (CD4+ CD8− CCL5− CCR10+)
T (CD4− CD8+ CCL5+)
T (CD4+ CD8+ CCL5+)
T (PF)
hematopoietic precursor
megakaryocyte prog
platelet
ery prog
ery lin
ery lin (PF)
neu
neu (PF)
neu (CCL13+) *
neu (IL18BP+) *
basophil
eosinophil

Supplementary Fig. 3 (p. 4)

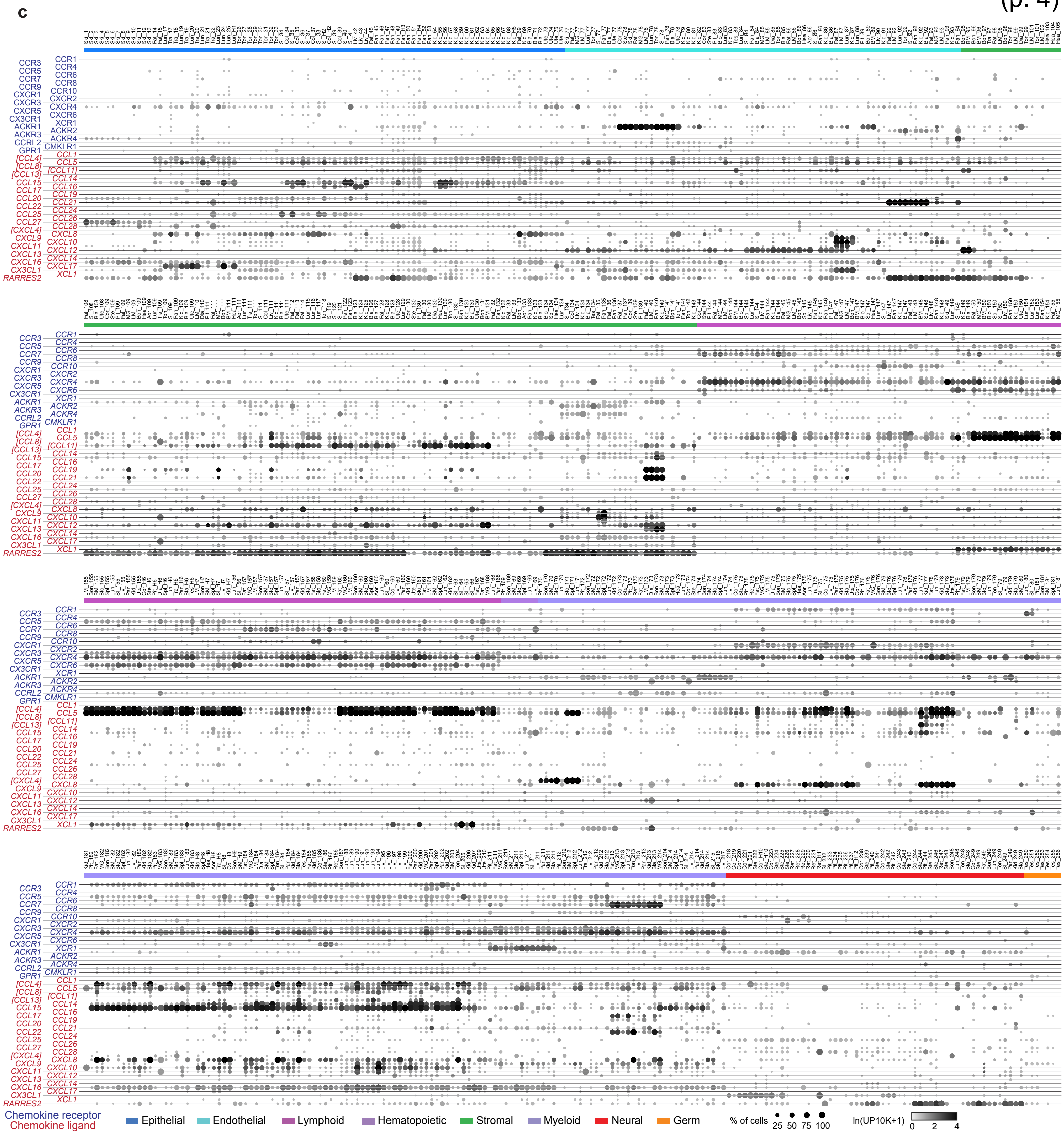

Supplementary Fig. 4

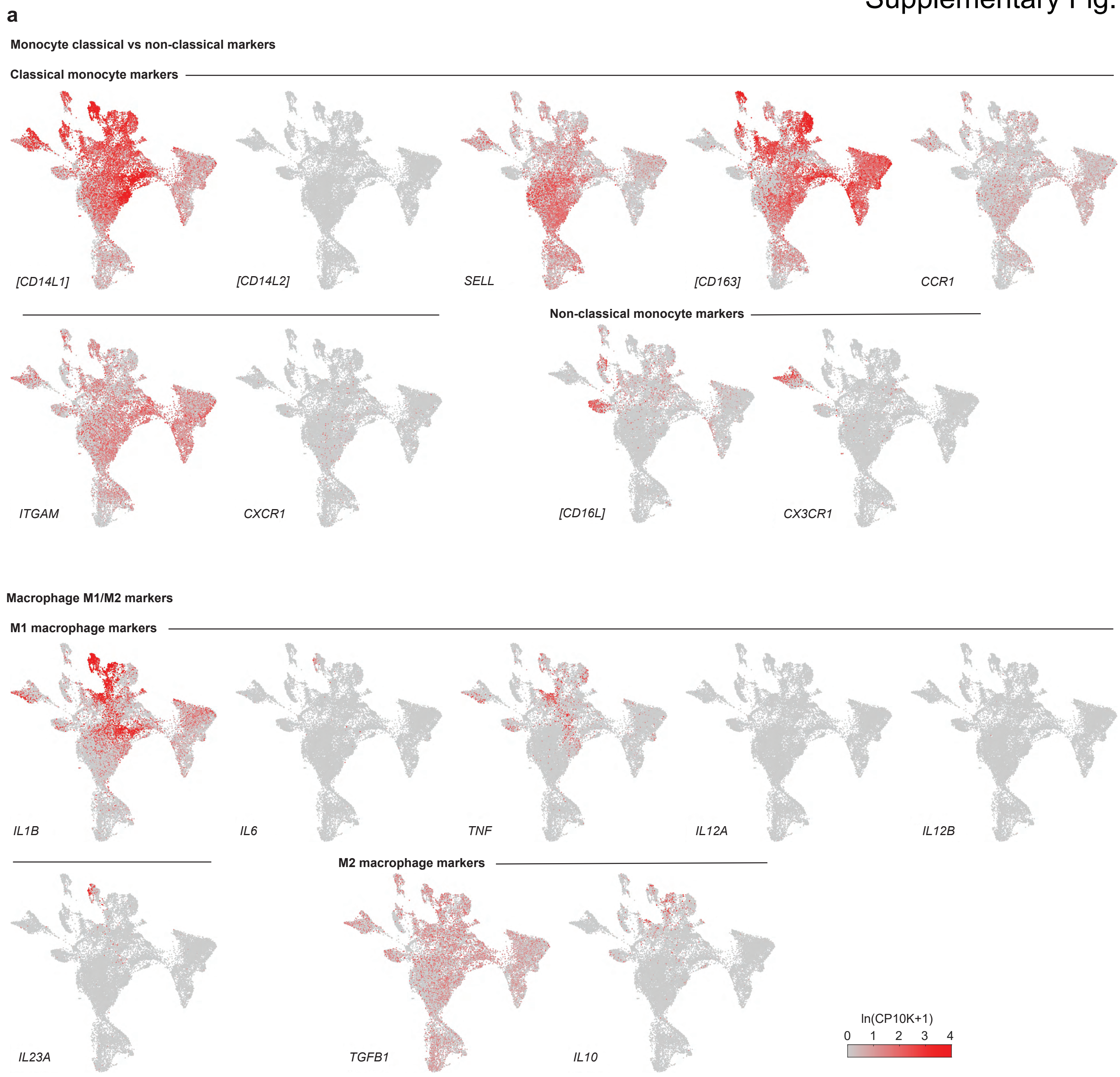

Supplementary Fig. 4
(p. 2)

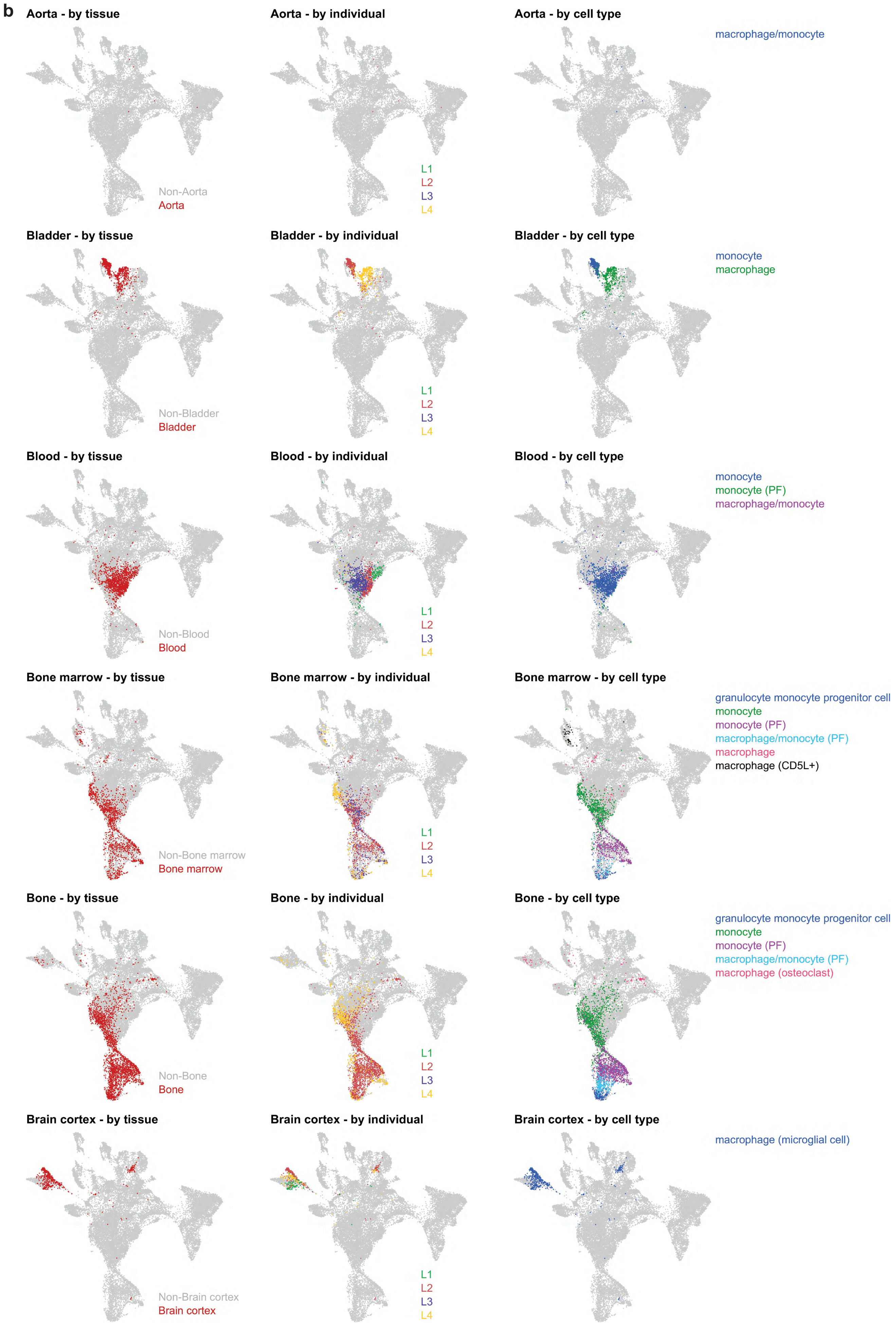

Supplementary Fig. 4
(p. 3)

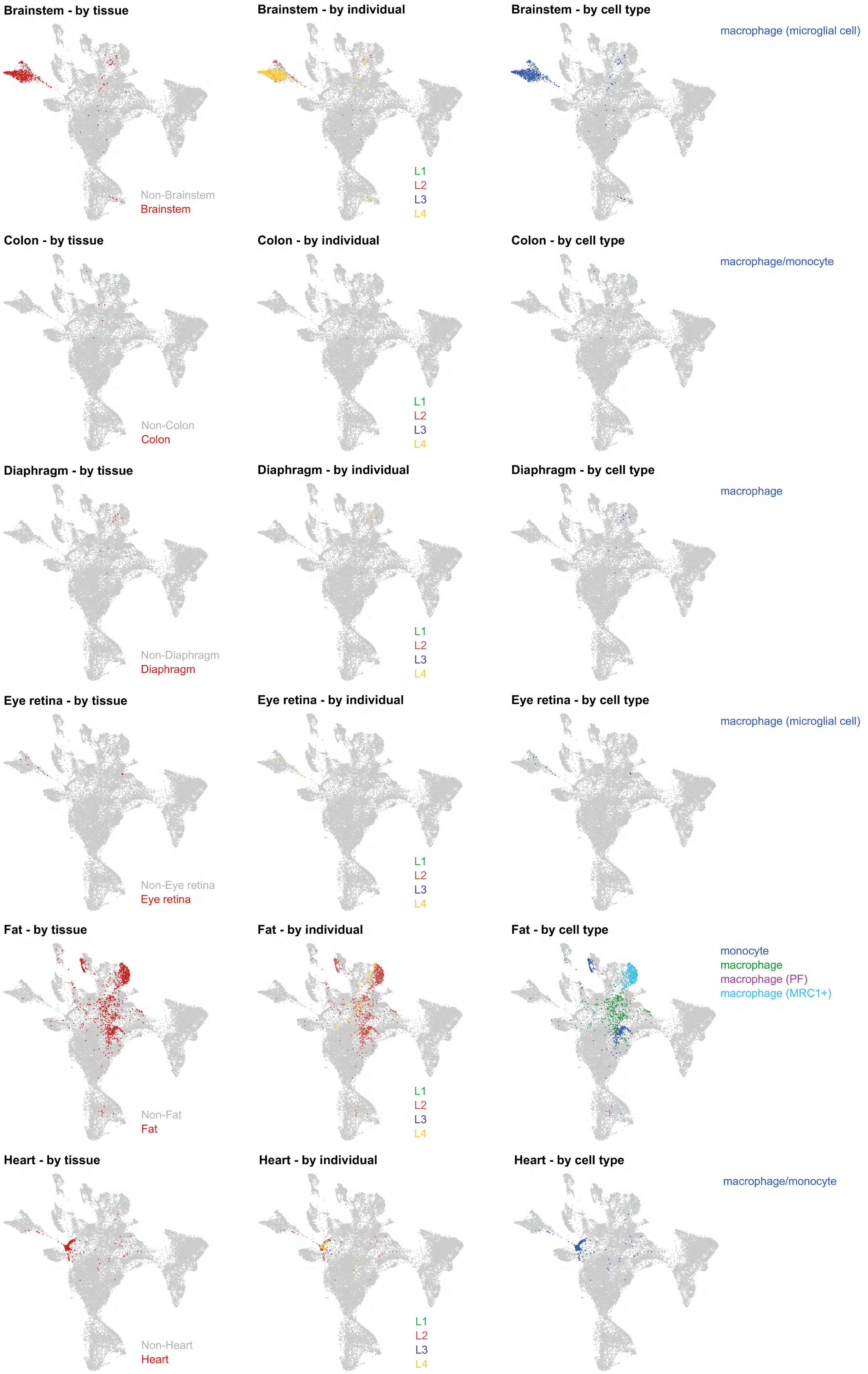

Supplementary Fig. 4
(p. 4)

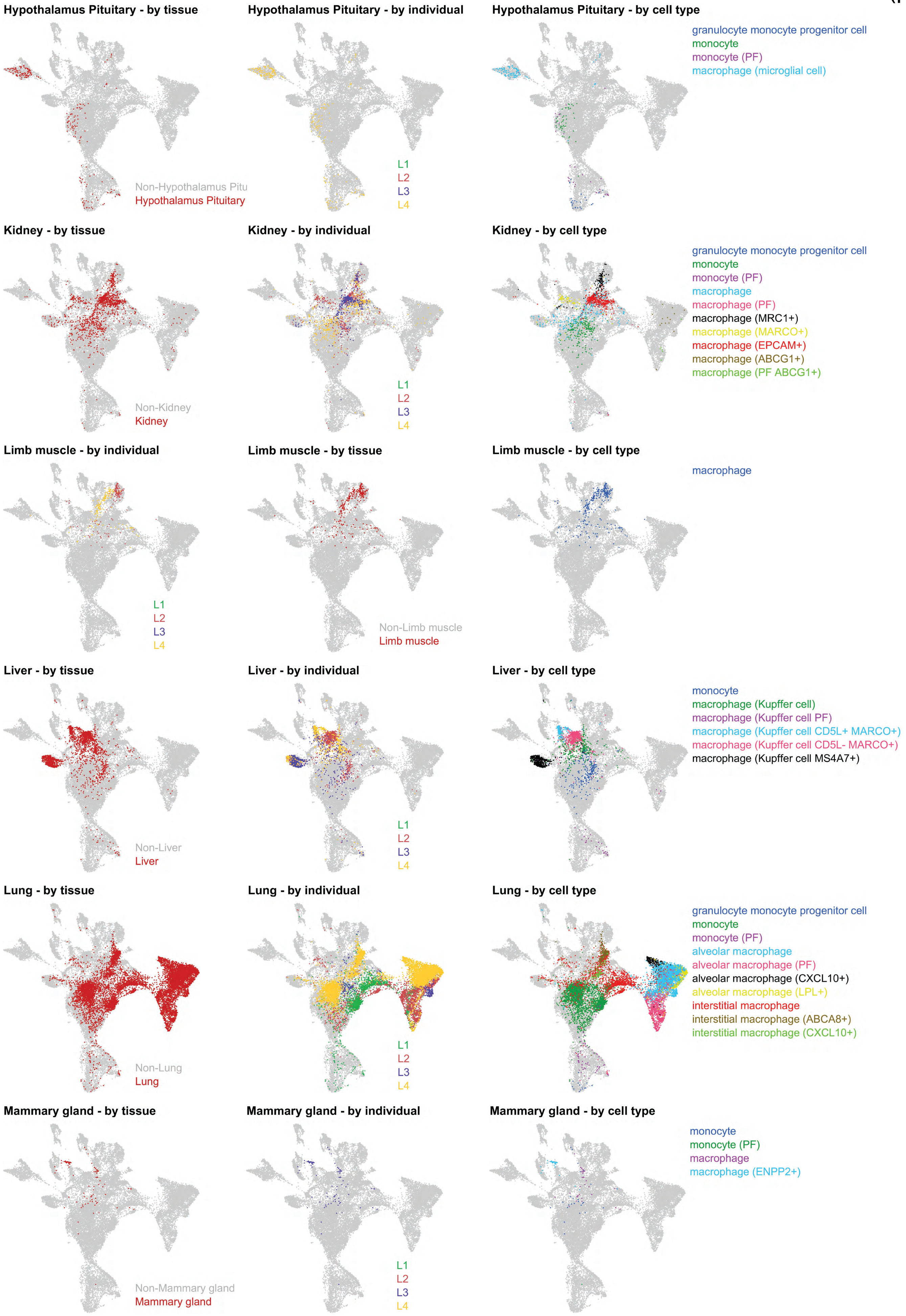

Supplementary Fig. 4
(p. 5)

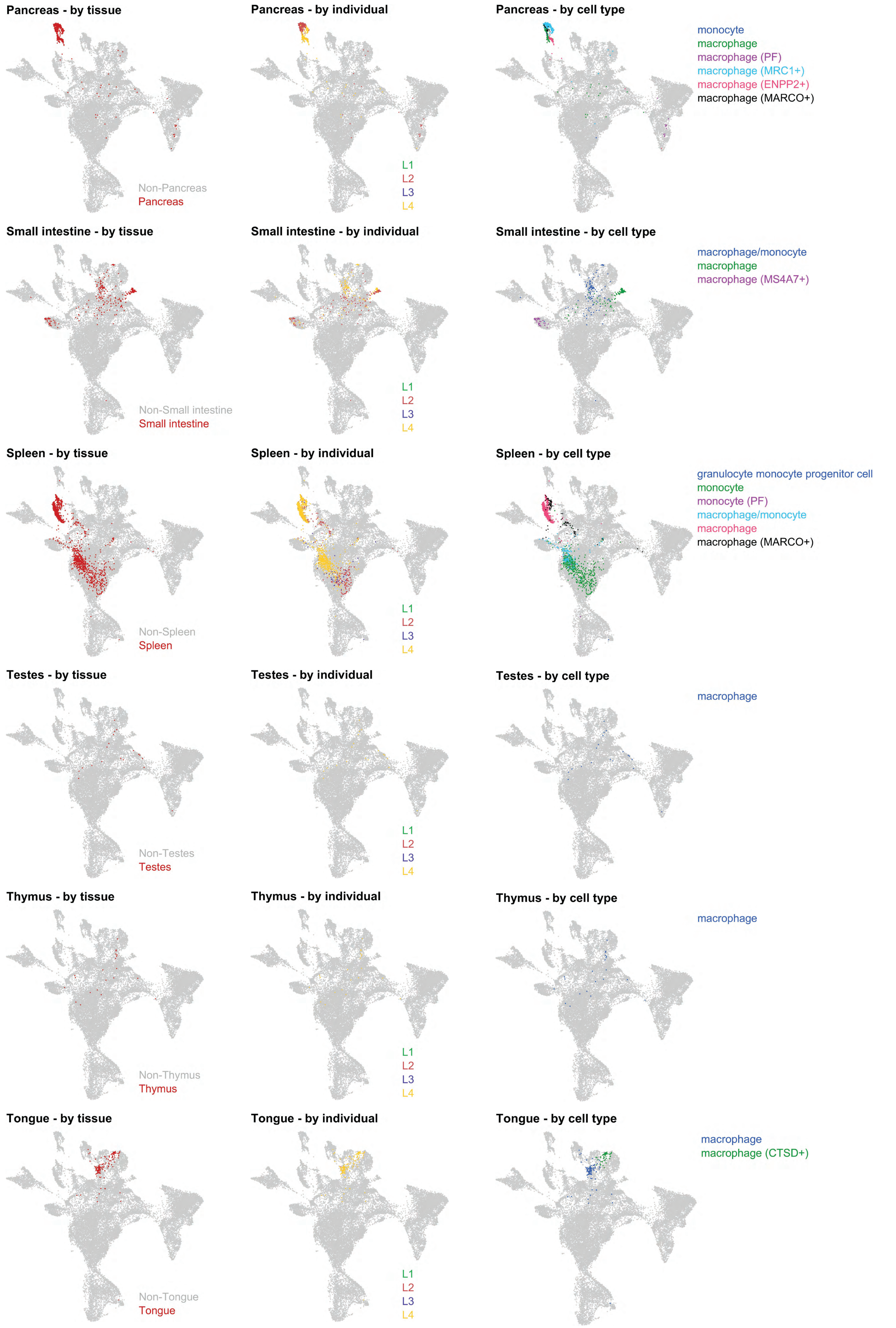

Supplementary Fig. 4
(p. 6)

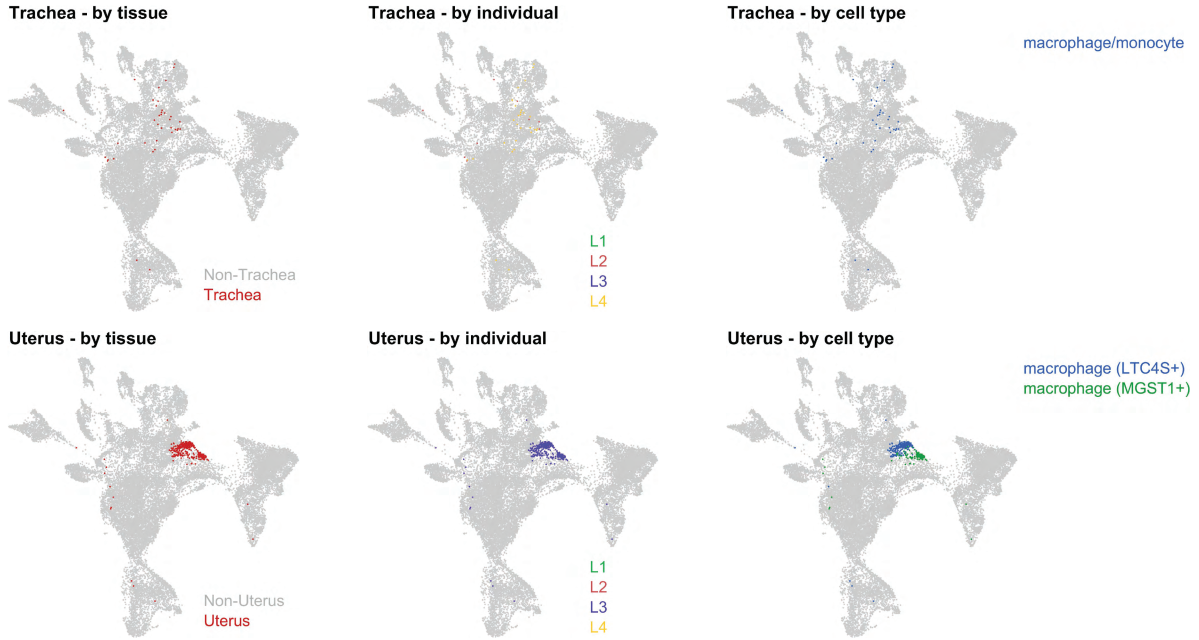

a PS genes enriched in epithelial compartment

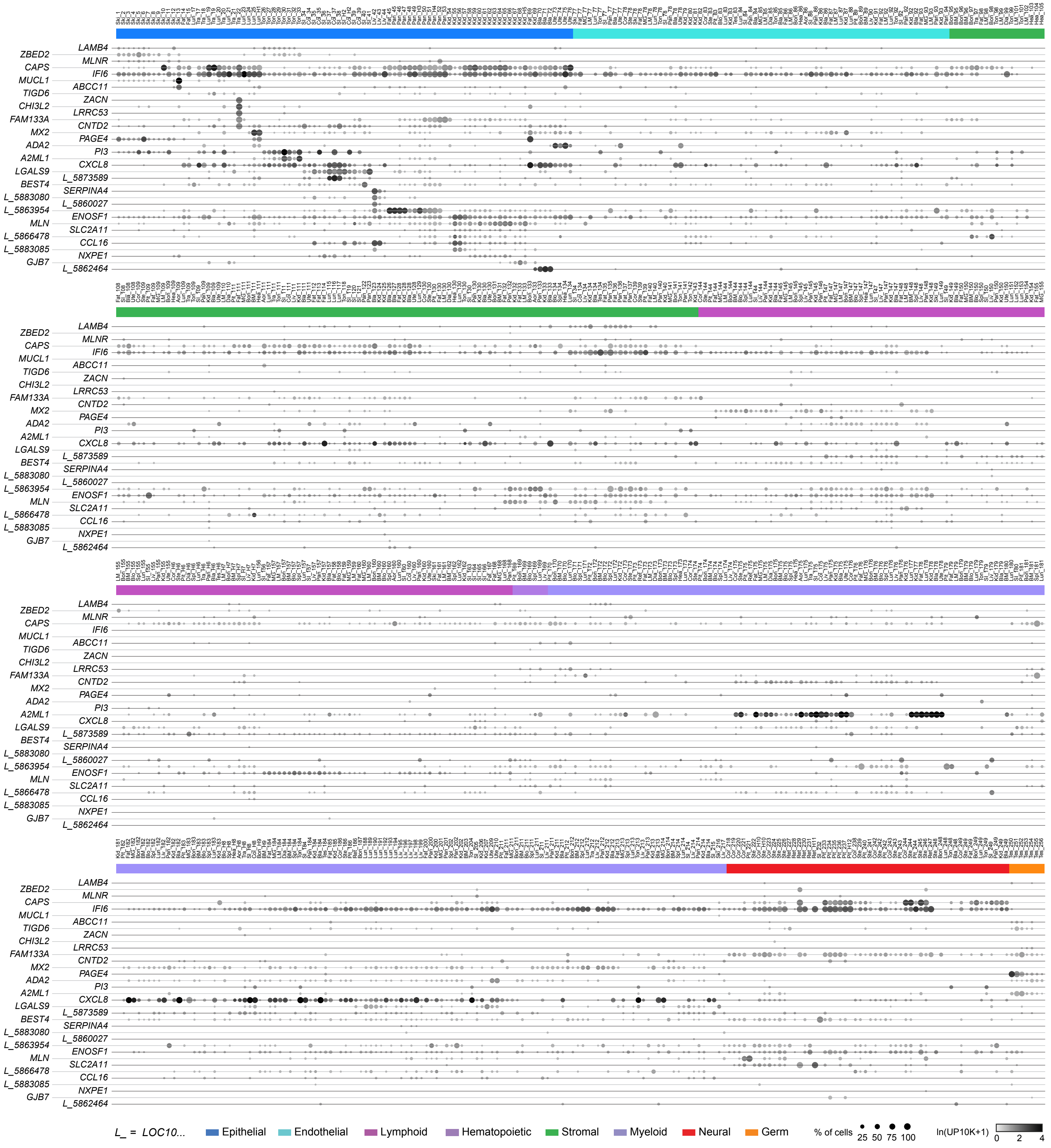

b PS genes enriched in endothelial compartment

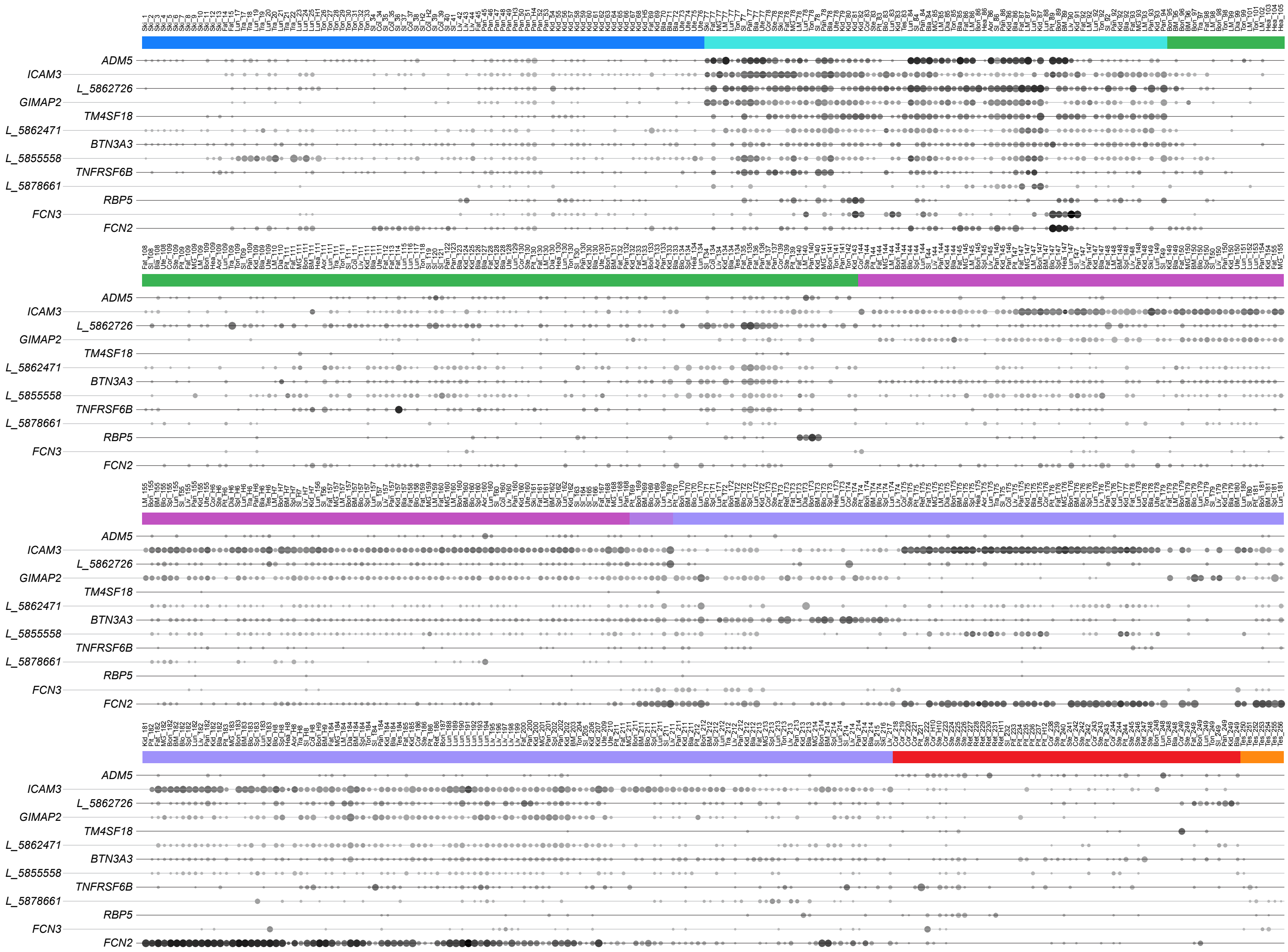

c PS genes enriched in stromal compartment

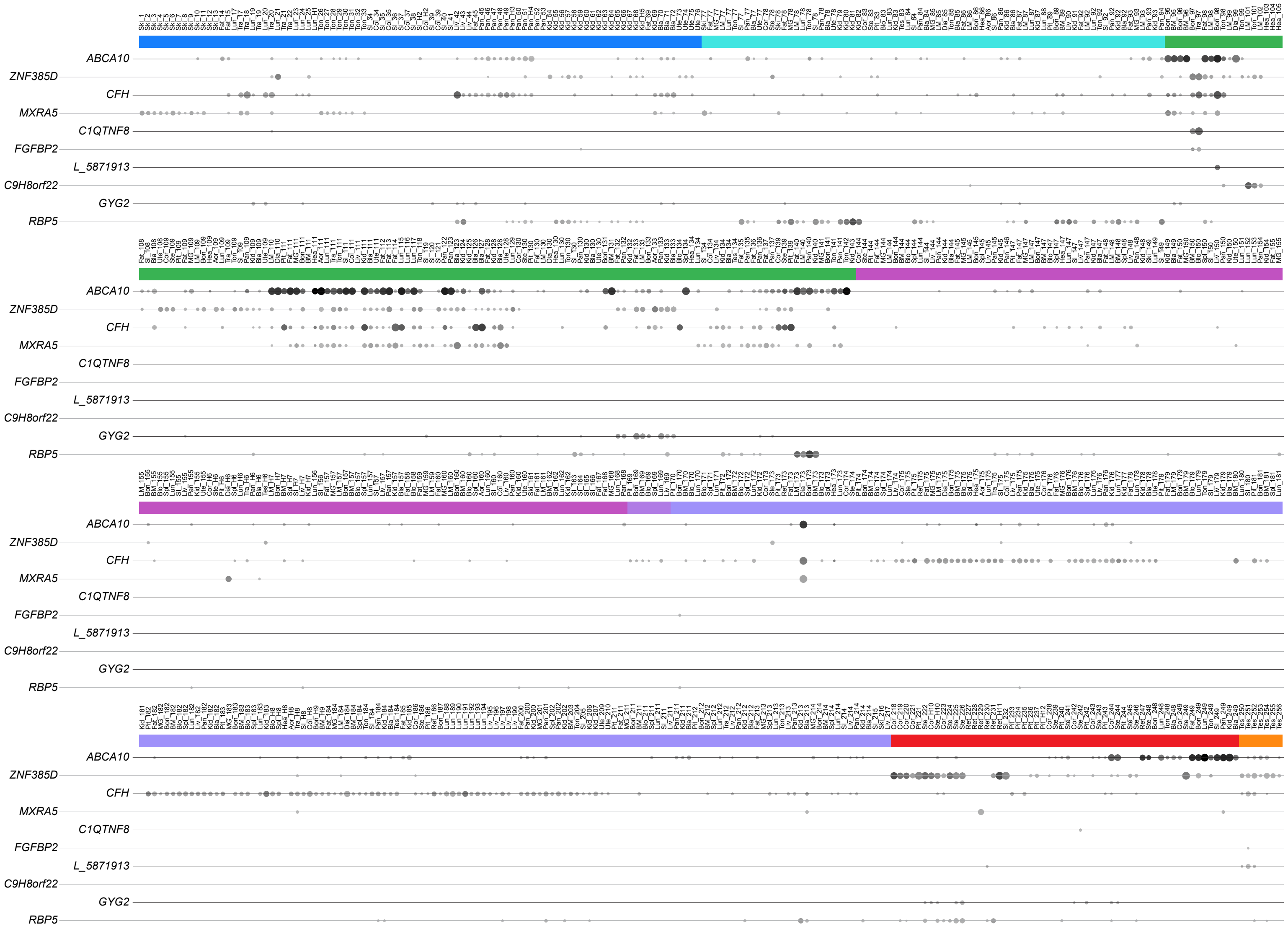

d PS genes enriched in immune compartment

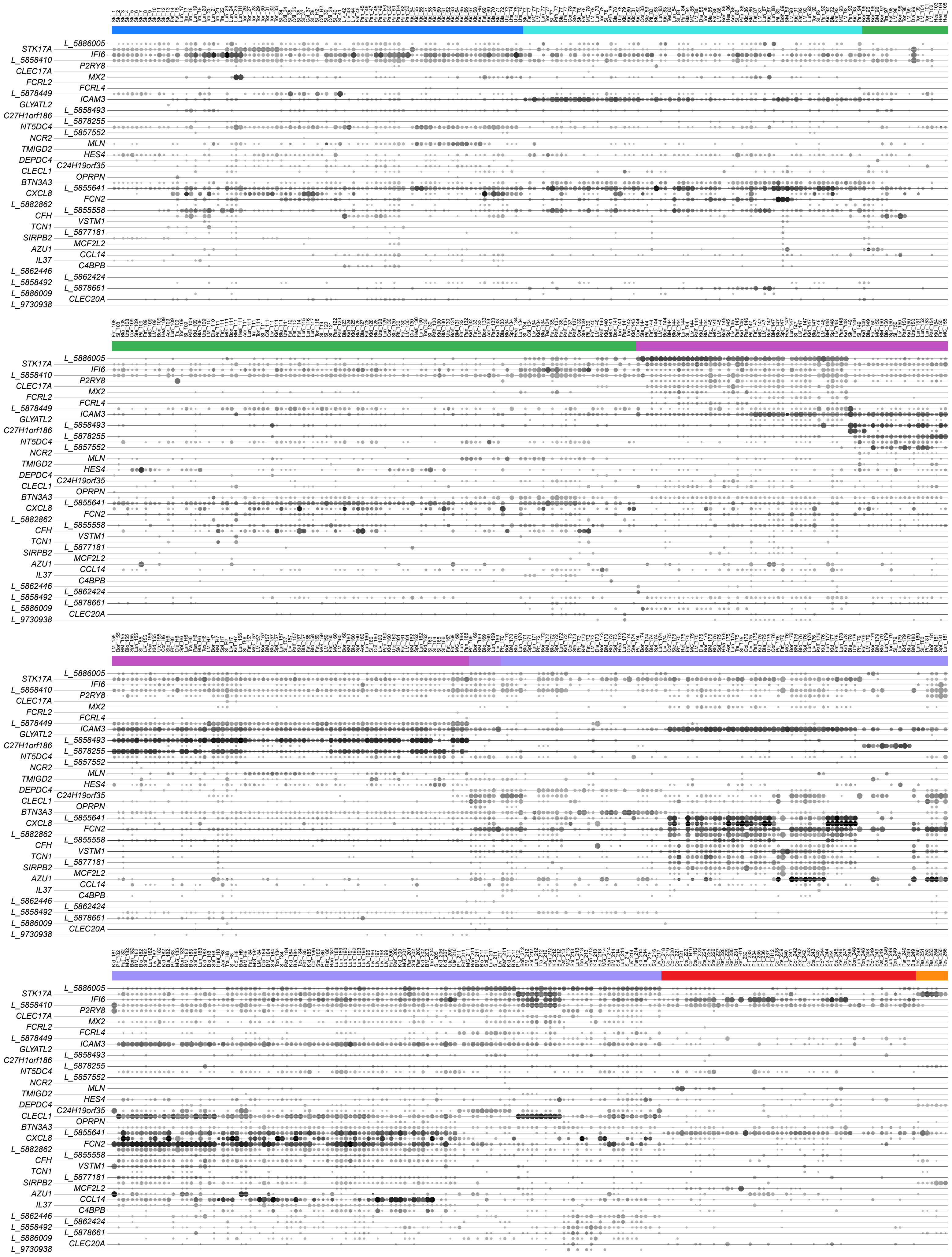

e PS genes enriched in neural compartment

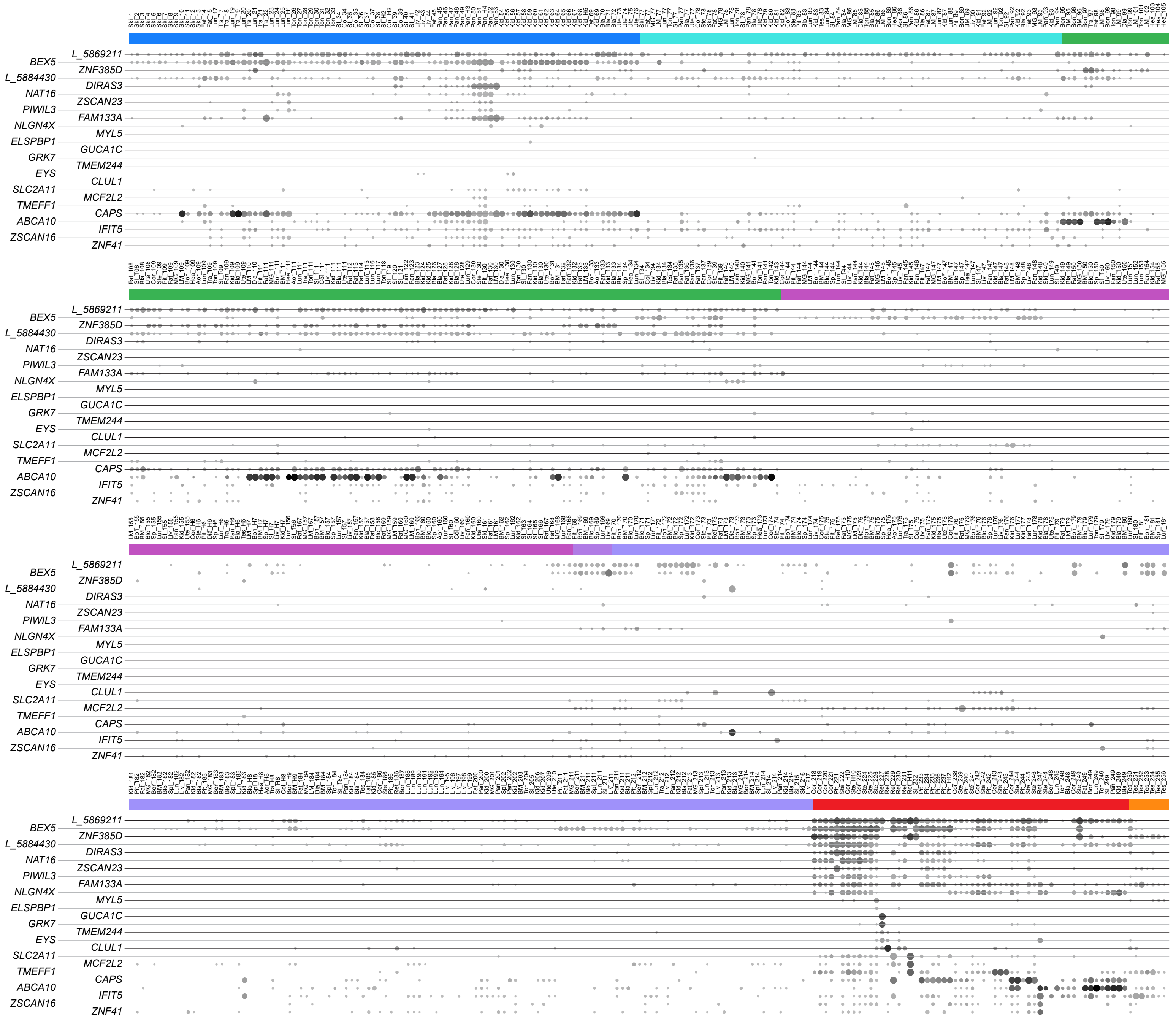

f PS genes enriched in germ compartment

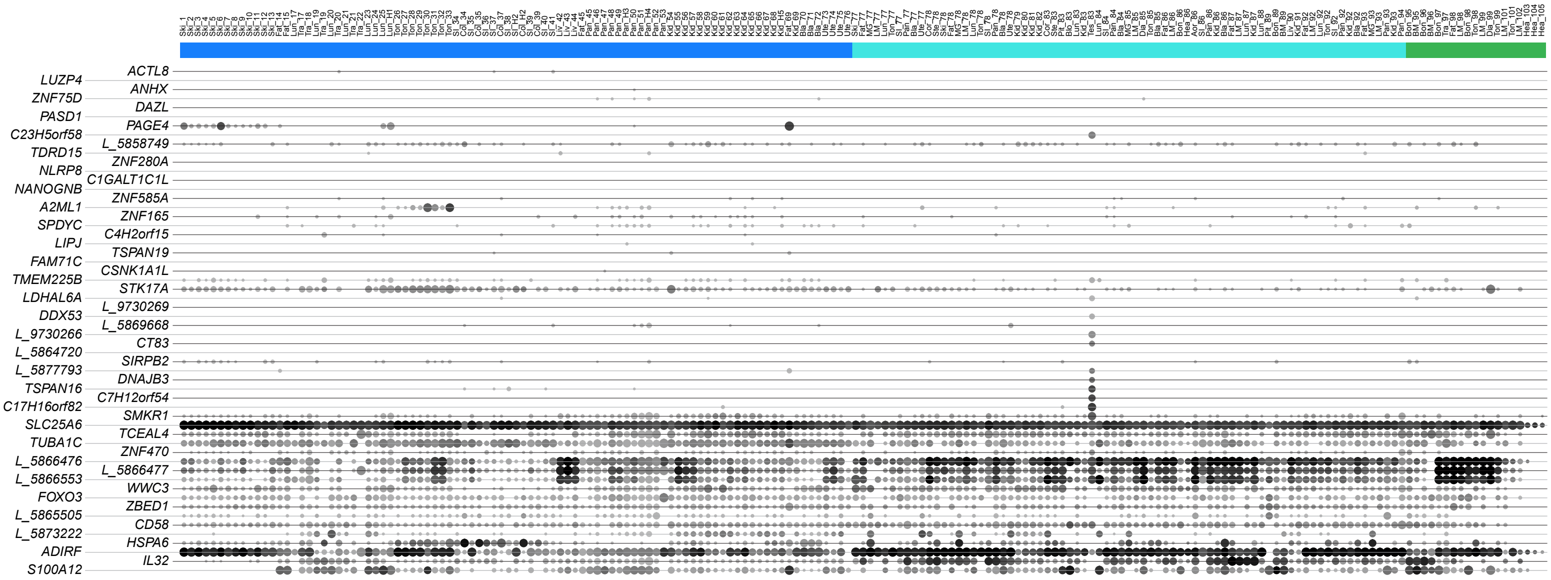

Supplementary Fig. 5 (p. 6)

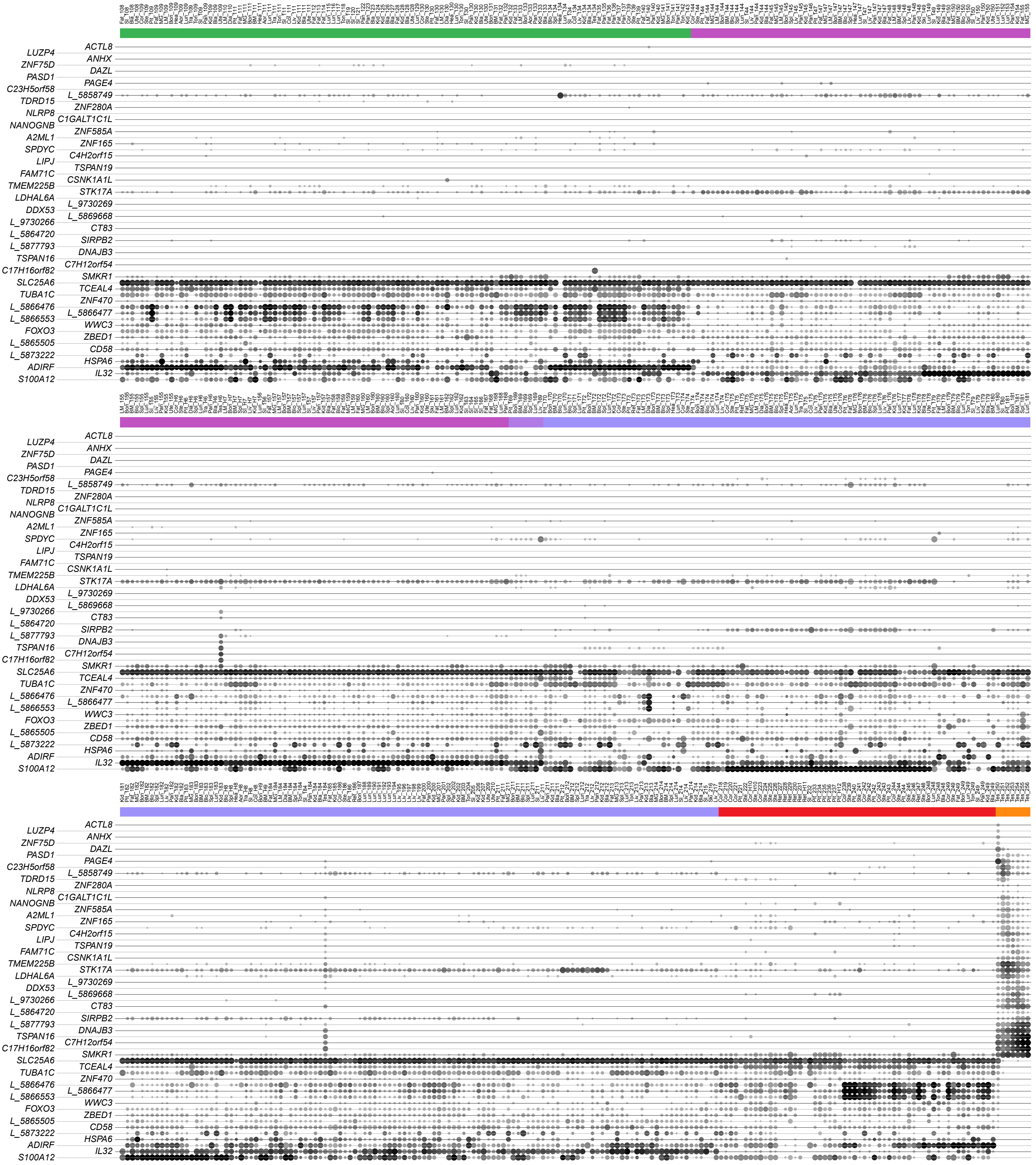

PSG gene expression in human vs. lemur

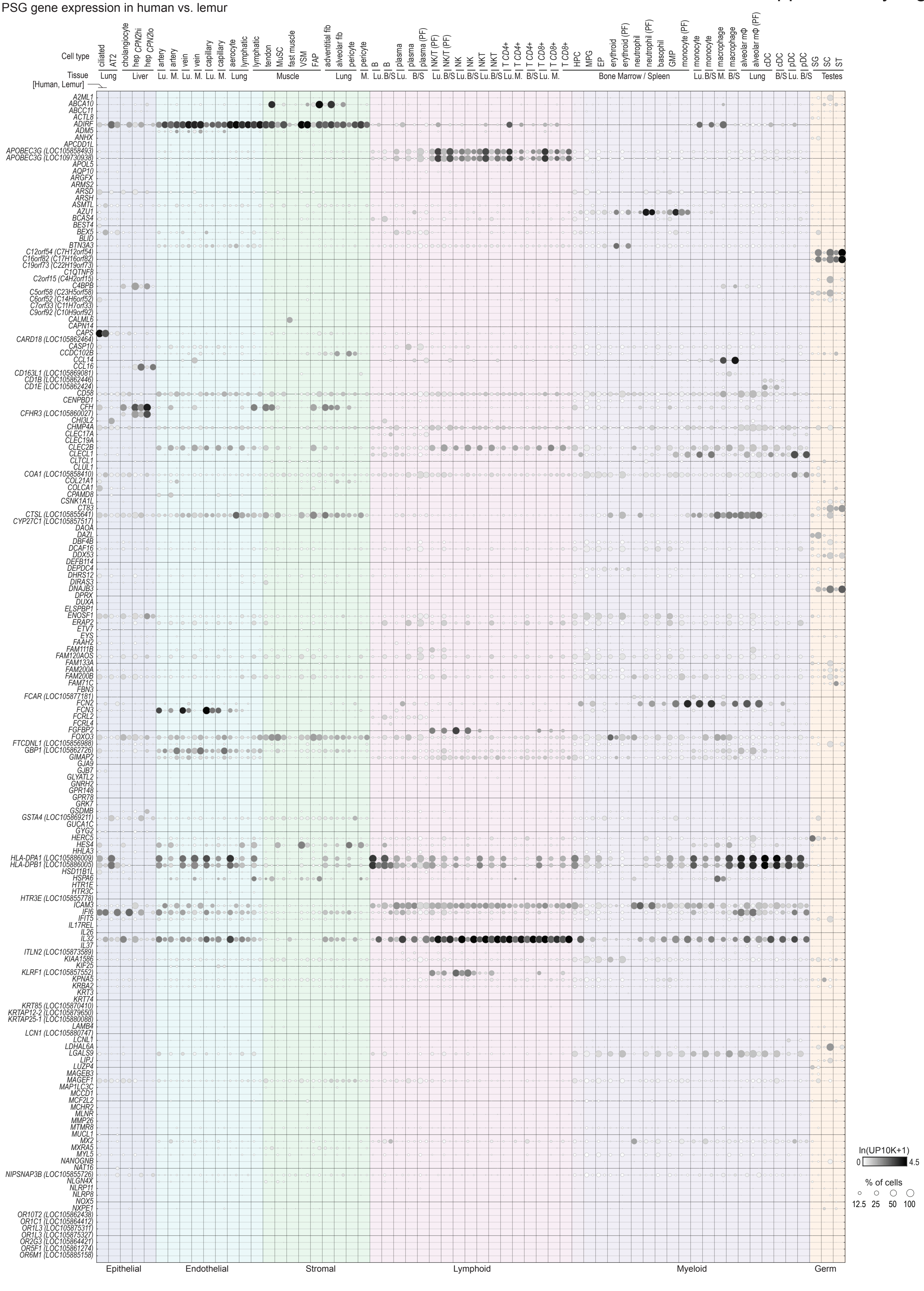

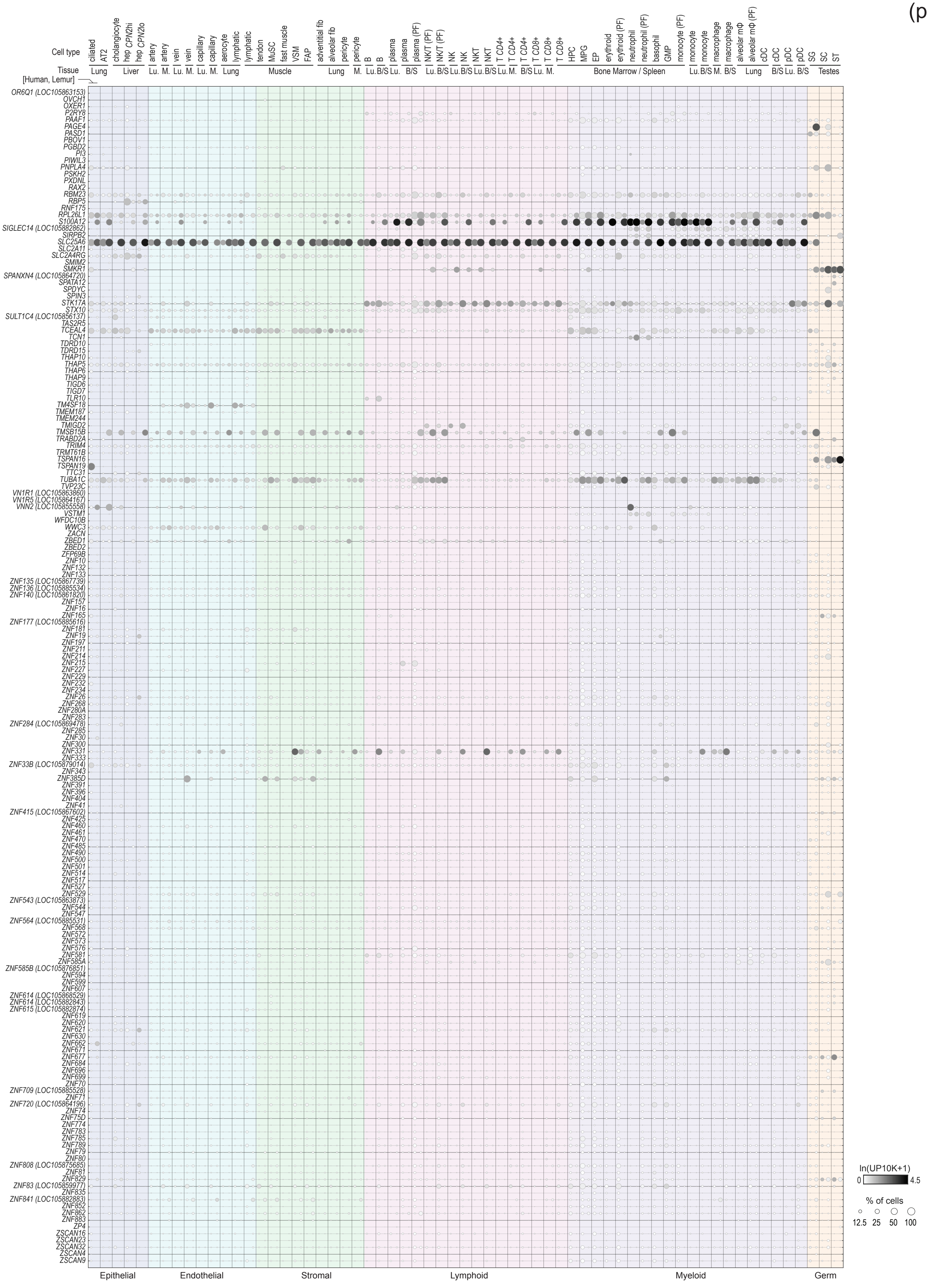
Cell type
Tissue
[Human, Lemur]
ciliated
AT2
cholangiocyte
hep CPN2hi
hep CPN2lo
artery
artery
vein
vein
capillary
capillary
aerocyte
lymphatic
lymphatic
tendon
MuSC
fast muscle
VSM
FAP
adventitial fib
alveolar fib
pericyte
pericyte
B
B
plasma
plasma (PF)
NK/T (PF)
NK/T (PF)
NK
NK
NKT
NKT
T CD4+
T CD4+
T CD4+
T CD8+
T CD8+
T CD8+
HPC
MPG
EP
erythroid
erythroid (PF)
neutrophil
neutrophil (PF)
basophil
GMP
monocyte (PF)
monocyte
monocyte
macrophage
macrophage
alveolar mΦ
alveolar mΦ (PF)
cDC
cDC
pDC
pDC
SG
SC
ST
Lung
Liver
Lu. M. Lu. M. Lu. M.
Lung
Muscle
Lung
M.
Lu. B/S Lu. B/S Lu. B/S Lu. B/S Lu. B/S Lu. M. B/S Lu. M.
Bone Marrow / Spleen
Lu. B/S M. B/S
Lung
B/S Lu. B/S
Testes
OR6Q1 (LOC105863153)
OVCH1
OXER1
P2RY8
PAAF1
PAGE4
PASD1
PBOV1
PGBD2
PI3
PIWIL3
PNPLA4
PSKH2
PXDNL
RAX2
RBM23
RBP5
RNF175
RPL26L1
S100A12
SIGLEC14 (LOC105882862)
SIRPB2
SLC25A6
SLC2A11
SLC2A4RG
SMIM2
SMKR1
SPANXN4 (LOC105864720)
SPATA12
SPDYC
SPIN3
STK17A
STX10
SULT1C4 (LOC105856137)
TAS2R5
TCEAL4
TCN1
TDRD10
TDRD15
THAP10
THAP5
THAP6
THAP9
TIGD6
TIGD7
TLR10
TM4SF18
TMEM187
TMEM244
TMIGD2
TMSB15B
TRABD2A
TRIM4
TRMT61B
TSPAN16
TSPAN19
TTC31
TUBA1C
TVP23C
VN1R1 (LOC105863860)
VN1R5 (LOC105864167)
VNN2 (LOC105855558)
VSTM1
WFDC10B
WWC3
ZACN
ZBED1
ZBED2
ZFP69B
ZNF10
ZNF132
ZNF133
ZNF135 (LOC105867739)
ZNF136 (LOC105885534)
ZNF140 (LOC105861820)
ZNF157
ZNF16
ZNF165
ZNF177 (LOC105885616)
ZNF181
ZNF19
ZNF197
ZNF211
ZNF214
ZNF215
ZNF227
ZNF229
ZNF232
ZNF234
ZNF26
ZNF268
ZNF280A
ZNF283
ZNF284 (LOC105869478)
ZNF285
ZNF30
ZNF300
ZNF331
ZNF333
ZNF33B (LOC105879014)
ZNF343
ZNF385D
ZNF391
ZNF396
ZNF404
ZNF41
ZNF415 (LOC105867602)
ZNF425
ZNF460
ZNF461
ZNF470
ZNF485
ZNF490
ZNF500
ZNF501
ZNF514
ZNF517
ZNF527
ZNF529
ZNF543 (LOC105863873)
ZNF544
ZNF547
ZNF564 (LOC105885531)
ZNF568
ZNF572
ZNF573
ZNF576
ZNF581
ZNF585A
ZNF585B (LOC105876851)
ZNF594
ZNF599
ZNF607
ZNF614 (LOC105868529)
ZNF614 (LOC105882843)
ZNF615 (LOC105882874)
ZNF619
ZNF620
ZNF621
ZNF630
ZNF662
ZNF671
ZNF677
ZNF684
ZNF696
ZNF699
ZNF70
ZNF709 (LOC105885528)
ZNF71
ZNF720 (LOC105864196)
ZNF74
ZNF75D
ZNF774
ZNF783
ZNF785
ZNF789
ZNF79
ZNF80
ZNF808 (LOC105875685)
ZNF81
ZNF829
ZNF83 (LOC105859977)
ZNF835
ZNF841 (LOC105882883)
ZNF852
ZNF862
ZNF883
ZP4
ZSCAN16
ZSCAN23
ZSCAN32
ZSCAN4
ZSCAN9
Epithelial
Endothelial
Stromal
Lymphoid
Myeloid
Germ
ln(UP10K+1)
0
4.5
% of cells
12.5 25 50 100

Supplementary Table Legends

Supplementary Table 1. Differentially-expressed-uTARs and their homologous genes and cellular expression levels.
Table 1a ("DE uTAR characteristics") lists the 4003 unique differentially-expressed (DE) uTARs detected in the atlas. Columns include: uTAR identifier (unique uTAR ID that appends genomic position, strandedness (+/- strand), and total count of aligned reads followed by a "-0", indicating the TAR region is a uTAR), fasta (uTAR location in the genome in the FASTA format, i.e., chromosome ID followed by the start and end locations), fastaPeak (full width at half maximum region around the absolute peak in coverage after Gaussian smoothing within the uTAR location), tissue_cell type_all (list of cell types that the uTAR is differentially-expressed in each atlas tissue), num_tissue_cell type (number of cell types the uTAR is differentially expressed in), tissue_cell type_top (the cell type with the most significant *p*-value in the differential expression test), avg_logFC (log(e) fold increase of the expression in the indicated top cell type compared to other 10x cell types in the same tissue), p_val_adj (*p*-value of enriched expression in the indicated top cell type, adjusted by Bonferroni correction), pct.1 (percent of cells in the indicated top cell type expressing the uTAR), pct.2 (proportion of other 10x cells in the same tissue expressing the uTAR), blastShort (most frequent significant BLASTn result for the entire length of the uTAR), blastShort_peak (most frequent significant BLASTn result for the peak region in the uTAR). The table indicates whether the DE-uTAR nucleotide sequence has homology with a human gene that has no annotated mouse or lemur orthologues (is_H=TRUE) suggesting it is a PS gene missed in the current lemur NCBI annotation; or with a human gene that has a mouse orthologue and no annotated lemur orthologues (is_HM=TRUE) suggesting it is a conserved gene across all three species missed in the current lemur annotation. The table also highlights DE-uTARs that overlap in genomic position with the mouse lemur Ensembl annotation but that is missing in NCBI (is_EnsemblOnly=TRUE).
Table 1b ("uTAR cellular expression") lists the identity of each cell (cell ID, tissue source, individual source, cell ontology, and free annotation, as described in https://tabula-microcebus.ds.czbiohub.org/whereisthedata) and its TAR characteristics including for each cell the percentages of reads (UMIs) detected by the TAR analysis that are annotated TARs (percent_aTAR) as well as unannotated TARs (percent_uTAR) separated by differentially-expressed uTARs (percent_uTAR_DE) and not differentially-expressed uTARs (percent_uTAR_nonDE).

Supplementary Table 2. uTAR expression counts across 10x dataset
TSV file with UMI count matrix of the DE-uTARs across all 10x cells, with each entry being a non-zero element of the matrix followed by the corresponding cell index (index_cell), uTAR index (index_uTAR), and number of UMI (n_reads). The uTAR and cell indexes are as indicated in Supplementary Table 1a and 1b, respectively.

Supplementary Table 3. Splice junctions and categories identified by the SICILIAN analysis.
Table lists all splice junctions detected by SICILIAN. Each row corresponds to a distinct junction. Columns include: junction_ID (unique name for each junction providing chromosome name, gene name, coordinate, and strand orientation for the 5' and 3' sides of the junction), num_reads_total (total number of reads mapped to the junction across all 10x and SS2 datasets),

chromosome_refseq_ID (RefSeq ID for the chromosome), chromosome_name (chromosome name), junction_5SS (5' splice site coordinate), junction_3SS (3' splice site coordinate), gene_5SS (gene name for the 5' side of the junctions), gene_3SS (gene name annotation for the 3' side of the junctions), strand (strand orientation), conservation (conservation status with human and/or mouse as defined in Fig. 1i), category (annotation category as defined in Fig. 1g).

Supplementary Table 4. Genes differentially spliced across cell types.
Table of genes with statistically significant splicing differences across cell types in the same tissue for at least two lemurs, detected by MANOVA test (two-tailed) using the 10x atlas dataset. Each row lists a unique combination of gene, tissue source, and lemur individual source. Additional columns include pval_zscore_adj (the p-value resulting from the MANOVA test) and num_cell_types (number of cell types analyzed with the MANOVA test in the given tissue and individual for this gene).

Supplementary Table 5. Expression homologue triads of human, lemur, and mouse detected by SAMap.
Table lists expression homologue triads detected by SAMap analysis separately on the lung and skeletal muscle cross-species datasets (listed in column "Dataset"). Each row represents an expression triad with its type ('Type_expression_homolog'), gene symbols in the three species ('huGene', 'leGene', 'msGene') including its species prefix, status of lemur gene annotation in NCBI ('is_leGene_Named'), number of gene pairs in the triads that are orthologues ('N_ortholog_pair') or non-orthologues ('N_non-ortholog_pair'), pairwise orthology relationship between species ('huleOrthology', 'humsOrthology', 'lemsOrthology'), expression pattern correlation score between gene pairs ('huleCorr', 'humsCorr', 'lemsCorr'), and the cell types with selective expression of the gene in respective species ('huCellType', 'leCellType', 'msCellType').

Supplementary Table 6. Expression of immunoglobulin isotypes in atlas B cells and plasma cells.
Table lists atlas B cells and plasma cells (SS2 dataset) with the immunoglobulin isotype identified by the scRNA-seq profile of each cell, including the expressed constant region isotype (heavy_constant_isotype, light_constant_isotype) and variable region family member (heavy_variable_family, light_variable_family) of heavy and light chains, as well as the complementarity-determining region (CDR3) amino acid sequence and length of the heavy (CDRH3_aa_sequence, CDRH3_aa_length) and light chains (CDRL3_aa_sequence, CDRL3_aa_length). Blank entries in these columns indicate cells for which the isotype could not be assigned (see Methods). Pairs or triplets of cells identified as part of the same clonal lineage, based on their CDRH3 and CDRL3 sequence similarity, are given the same number in column 'clone_number'.

Supplementary Table 7. Chemokine receptor and cognate ligand genes.
Table lists the analyzed lemur orthologues designated by NCBI ('receptor_lemur_ortholog', 'ligand_lemur_ortholog') of the 25 human chemokine receptors and their cognate ligands, retrieved from CellPhoneDB (March 2024). The source of each curated ligand-receptor interaction is given under 'cellphoneDB_annotation_strategy', 'cellphoneDB_curator', and 'cellphoneDB_source' (CellphoneDB output, 'curated' indicates it was annotated by

CellPhoneDB developers), and the type of signaling interaction is listed under 'cellphoneDB_classification'. For receptors with multiple ligands, the primary ligand(s) (when known) are noted under 'comments'.

Supplementary Table 8. Human genes and their lemur and mouse orthology assignments.
Table 8a ("Summary of human orthology assignments") summarizes the orthology status of all human protein-coding genes annotated in NCBI (19,966), beginning with the NCBI gene ID, synonym, gene description, and gene type. The presence of corresponding mouse lemur orthologues assigned by NCBI and/or Ensembl (as detailed in Methods) is indicated in columns 'HumanLemur_NCBI_HomologyType' and 'HumanLemur_Ensembl_HomologyType', respectively. Similar columns are provided for corresponding mouse orthologues assigned by NCBI, Ensembl, and/or MGI. For every human gene, column 'any_Lemur_homology' summarizes if there is at least one lemur orthologue assigned by NCBI and/or Ensembl and column 'any_Mouse_homology' summarizes if there is at least one mouse orthologue assigned by NCBI, Ensembl, and/or MGI. Human genes that are primate-selective/mouse-absent (539) are identified in the column 'HumanLemurOrthogs_NotMouse' (1: 'any_Lemur_homology' = yes and 'any_Mouse_homology' = no; 0: all other entries).
Table 8b ("All human orthology assignments") lists all human protein-coding genes annotated in NCBI (19,966) with corresponding NCBI gene ID, synonym, external database cross references (dbXrefs), chromosome location, map location, gene description, gene type, as well as corresponding Ensembl gene ID, gene name and OMIM associated phenotype description. The presence of corresponding mouse lemur orthologues assigned by NCBI and/or Ensembl (as detailed in Methods) is indicated in columns 'HumanLemur_NCBI_HomologyType' and 'HumanLemur_Ensembl_HomologyType', respectively, with the corresponding NCBI gene ID, gene name, and synonym as well as Ensembl gene ID and gene name listed for lemur genes with an assigned orthologue in either of these databases. Similar columns are provided for the corresponding mouse orthologues assigned by NCBI, Ensembl, and/or MGI. Rows are duplicated for each human NCBI gene with more than one corresponding Ensembl gene, or that is assigned to more than one lemur and/or mouse orthologue.

Supplementary Table 9. PS genes and their expression patterns in the mouse lemur and human.
List of mouse lemur PS genes annotated in NCBI (425), starting with the corresponding NCBI gene ID, synonym, external database cross references (dbXrefs), chromosome location, gene type, gene description, and, if present, the associate human OMIM genetic disorder phenotypes. For each gene, the compartments and tissues that have enriched expression of the gene are listed in columns 'Lemur_enriched_compartment' and 'Lemur_enriched_tissue', and summarized in column 'Lemur_expression_pattern_summary'. Cell types showing selective expression in the enriched compartments/tissues are noted in column 'Lemur_enriched_celltype'. Compartments and tissues that have selectively depleted expression are indicated by brackets. The number of lemur cell types (10x dataset, excluding cells labeled as mix, doublets or low quality) with mean expression of the gene greater than zero (column 'N_lemur_celltypes_wMeanExpGreaterThan0'), as well as the number of cell types with mean expression of the gene greater than zero and expressed in at least 1% of the cells in that cell type ('N_lemur_celltypes_wMeanExpGreaterThan0_ExpIn1PCTOfCells') are provided. The order of genes appearing in Supplementary Fig. 5 dot plots are provided in column 'Gene_order'. PS

genes (aTARs) found using the TAR analysis pipeline (see Methods - uTAR analysis) are indicated in the ‘Gene_inTARanalysis’ column. The next three columns indicate whether the PS gene has one-to-one orthology mapping (‘HL_Orthology_one2one’), and if yes the corresponding human orthologue gene ID and symbol (‘Human_NCBI_GeneID’, ‘Human_NCBI_GeneName’). Finally, the last two columns indicate whether the PS gene was also present in the human datasets used for the cross species analysis (‘scRNAseq_reported’) and the correlation coefficient of its human vs. lemur cell type expression patterns (‘HL_Expression_R’).

Supplementary Table 10. Gene sets enriched in PS genes.
Gene set enrichment analysis (gprofiler2[53] in R, Fisher's one-tailed test with multiple testing correction) for the 539 PS genes. Columns detailed in https://cran.r-project.org/web/packages/gprofiler2/vignettes/gprofiler2.html.

Supplementary References

1. Casey, K. M., Karanewsky, C. J., Pendleton, J. L., Krasnow, M. R. & Albertelli, M. A. Fibrous Osteodystrophy, Chronic Renal Disease, and Uterine Adenocarcinoma in Aged Gray Mouse Lemurs (Microcebus murinus). Comp. Med. 71, 256–266 (2021).
2. The Tabula Microcebus Consortium. A molecular cell atlas of mouse lemur, an emerging model primate.
3. Rock, K. L., Reits, E. & Neefjes, J. Present Yourself! By MHC Class I and MHC Class II Molecules. Trends Immunol. 37, 724–737 (2016).
4. Averdam, A. et al. Sequence analysis of the grey mouse lemur (Microcebus murinus) MHC class II DQ and DR region. Immunogenetics 63, 85–93 (2011).
5. Guethlein, L. A., Ezran, C., Liu, S., Krasnow, M. A. & Parham, P. Organism-wide mapping of MHC class I and II expression in mouse lemur cells and tissues. bioRxiv (2022) doi:10.1101/2022.02.28.482372.
6. Djaoud, Z. & Parham, P. HLAs, TCRs, and KIRs, a Triumvirate of Human Cell-Mediated Immunity. Annu. Rev. Biochem. 89, 717–739 (2020).
7. Averdam, A. et al. A novel system of polymorphic and diverse NK cell receptors in primates. PLoS Genet. 5, e1000688 (2009).
8. Norman, P. J. et al. Sequences of 95 human MHC haplotypes reveal extreme coding variation in genes other than highly polymorphic HLA class I and II. Genome Res. 27, 813–823 (2017).
9. Larsen, P. A. et al. Hybrid de novo genome assembly and centromere characterization of the gray mouse lemur (Microcebus murinus). BMC Biol. 15, 110 (2017).
10. Flügge, P., Zimmermann, E., Hughes, A. L., Günther, E. & Walter, L. Characterization and phylogenetic relationship of prosimian MHC class I genes. J. Mol. Evol. 55, 768–775 (2002).
11. Ito, T., Carson, W. F., 4th, Cavassani, K. A., Connett, J. M. & Kunkel, S. L. CCR6 as a mediator of immunity in the lung and gut. Exp. Cell Res. 317, 613–619 (2011).
12. Furue, K., Ito, T., Tsuji, G., Nakahara, T. & Furue, M. The CCL20 and CCR6 axis in psoriasis. Scand. J. Immunol. 91, e12846 (2020).
13. Barone, F. et al. Stromal Fibroblasts in Tertiary Lymphoid Structures: A Novel Target in Chronic Inflammation. Front. Immunol. 7, 477 (2016).
14. Farnsworth, R. H., Karnezis, T., Maciburko, S. J., Mueller, S. N. & Stacker, S. A. The Interplay Between Lymphatic Vessels and Chemokines. Front. Immunol. 10, 518 (2019).
15. Jalkanen, S. & Salmi, M. Lymphatic endothelial cells of the lymph node. Nat. Rev. Immunol. 20, 566–578 (2020).
16. Ulvmar, M. H. et al. The atypical chemokine receptor CCRL1 shapes functional CCL21 gradients in lymph nodes. Nat. Immunol. 15, 623–630 (2014).
17. Bryce, S. A. et al. ACKR4 on Stromal Cells Scavenges CCL19 To Enable CCR7-Dependent Trafficking of APCs from Inflamed Skin to Lymph Nodes. J. Immunol. 196, 3341–3353 (2016).
18. Nie, Y. et al. The role of CXCR4 in maintaining peripheral B cell compartments and humoral immunity. J. Exp. Med. 200, 1145–1156 (2004).
19. Fletcher, A. L., Acton, S. E. & Knoblich, K. Lymph node fibroblastic reticular cells in health and disease. Nat. Rev. Immunol. 15, 350–361 (2015).
20. Vermi, W. et al. Role of ChemR23 in directing the migration of myeloid and plasmacytoid dendritic cells to lymphoid organs and inflamed skin. J. Exp. Med. 201, 509–515 (2005).
21. Metzemaekers, M., Vanheule, V., Janssens, R., Struyf, S. & Proost, P. Overview of the Mechanisms that May Contribute to the Non-Redundant Activities of Interferon-Inducible CXC Chemokine Receptor 3 Ligands. Front. Immunol. 8, 1970 (2017).

22. Kochumon, S. et al. Adipose tissue gene expression of CXCL10 and CXCL11 modulates inflammatory markers in obesity: implications for metabolic inflammation and insulin resistance. Ther. Adv. Endocrinol. Metab. 11, 2042018820930902 (2020).
23. Capucetti, A., Albano, F. & Bonecchi, R. Multiple Roles for Chemokines in Neutrophil Biology. Front. Immunol. 11, 1259 (2020).
24. Shen, F., Huang, X., He, G. & Shi, Y. The emerging studies on mesenchymal progenitors in the long bone. Cell Biosci. 13, 105 (2023).
25. Ambrosi, T. H., Longaker, M. T. & Chan, C. K. F. A Revised Perspective of Skeletal Stem Cell Biology. Front Cell Dev Biol 7, 189 (2019).
26. Mercier, F. E., Ragu, C. & Scadden, D. T. The bone marrow at the crossroads of blood and immunity. Nat. Rev. Immunol. 12, 49–60 (2011).
27. Greenbaum, A. M. & Link, D. C. Mechanisms of G-CSF-mediated hematopoietic stem and progenitor mobilization. Leukemia 25, 211–217 (2011).
28. Adrover, J. M., Nicolás-Ávila, J. A. & Hidalgo, A. Aging: A Temporal Dimension for Neutrophils. Trends Immunol. 37, 334–345 (2016).
29. Casanova-Acebes, M. et al. Rhythmic modulation of the hematopoietic niche through neutrophil clearance. Cell 153, 1025–1035 (2013).
30. Hartlehnert, M. et al. Schwann cells promote post-traumatic nerve inflammation and neuropathic pain through MHC class II. Sci. Rep. 7, 12518 (2017).
31. Ivetic, A., Hoskins Green, H. L. & Hart, S. J. L-selectin: A Major Regulator of Leukocyte Adhesion, Migration and Signaling. Front. Immunol. 10, 1068 (2019).
32. Hong, C.-W. Current Understanding in Neutrophil Differentiation and Heterogeneity. Immune Netw. 17, 298–306 (2017).
33. Russo, R. C., Garcia, C. C., Teixeira, M. M. & Amaral, F. A. The CXCL8/IL-8 chemokine family and its receptors in inflammatory diseases. Expert Rev. Clin. Immunol. 10, 593–619 (2014).
34. Pan, Z. Z., Parkyn, L., Ray, A. & Ray, P. Inducible lung-specific expression of RANTES: preferential recruitment of neutrophils. Am. J. Physiol. Lung Cell. Mol. Physiol. 279, L658–66 (2000).
35. Metzemaekers, M., Gouwy, M. & Proost, P. Neutrophil chemoattractant receptors in health and disease: double-edged swords. Cell. Mol. Immunol. 17, 433–450 (2020).
36. Shimizu, Y. & Dobashi, K. CC-chemokine CCL15 expression and possible implications for the pathogenesis of IgE-related severe asthma. Mediators Inflamm. 2012, 475253 (2012).
37. Gschwandtner, M., Derler, R. & Midwood, K. S. More Than Just Attractive: How CCL2 Influences Myeloid Cell Behavior Beyond Chemotaxis. Front. Immunol. 10, 2759 (2019).
38. Mendez-Enriquez, E. & García-Zepeda, E. A. The multiple faces of CCL13 in immunity and inflammation. Inflammopharmacology 21, 397–406 (2013).
39. Hickman, S. E. et al. The microglial sensome revealed by direct RNA sequencing. Nat. Neurosci. 16, 1896–1905 (2013).
40. Jurga, A. M., Paleczna, M. & Kuter, K. Z. Overview of General and Discriminating Markers of Differential Microglia Phenotypes. Front. Cell. Neurosci. 14, 198 (2020).
41. Bonnardel, J. et al. Stellate Cells, Hepatocytes, and Endothelial Cells Imprint the Kupffer Cell Identity on Monocytes Colonizing the Liver Macrophage Niche. Immunity 51, 638–654.e9 (2019).
42. Evren, E. et al. Distinct developmental pathways from blood monocytes generate human lung macrophage diversity. Immunity 54, 259–275.e7 (2021).

43. Davies, L. C., Jenkins, S. J., Allen, J. E. & Taylor, P. R. Tissue-resident macrophages. Nat. Immunol. 14, 986–995 (2013).
44. Todd, E. M. et al. Alveolar macrophage development in mice requires L-plastin for cellular localization in alveoli. Blood 128, 2785–2796 (2016).
45. Tsukasaki, M. et al. Stepwise cell fate decision pathways during osteoclastogenesis at single-cell resolution. Nat Metab 2, 1382–1390 (2020).
46. Orekhov, A. N. et al. Monocyte differentiation and macrophage polarization. Vessel Plus 3, 10 (2019).
47. Italiani, P. & Boraschi, D. From Monocytes to M1/M2 Macrophages: Phenotypical vs. Functional Differentiation. Front. Immunol. 5, 514 (2014).
48. Martinez, F. O. & Gordon, S. The M1 and M2 paradigm of macrophage activation: time for reassessment. F1000Prime Rep. 6, 13 (2014).
49. Wu, X. et al. Human Liver Macrophage Subsets Defined by CD32. Front. Immunol. 11, 2108 (2020).
50. MacParland, S. A. et al. Single cell RNA sequencing of human liver reveals distinct intrahepatic macrophage populations. Nat. Commun. 9, 4383 (2018).
51. Aizarani, N. et al. A human liver cell atlas reveals heterogeneity and epithelial progenitors. Nature 572, 199–204 (2019).
52. Shi, C. & Pamer, E. G. Monocyte recruitment during infection and inflammation. Nat. Rev. Immunol. 11, 762–774 (2011).
53. Kolberg, L., Raudvere, U., Kuzmin, I., Vilo, J. & Peterson, H. gprofiler2 -- an R package for gene list functional enrichment analysis and namespace conversion toolset g:Profiler. F1000Res. 9, 709 (2020).
